# β-Hairpin Peptide Mimics Decrease Human Islet Amyloid Polypeptide (hIAPP) Aggregation

**DOI:** 10.3389/fcell.2021.729001

**Published:** 2021-09-16

**Authors:** Jacopo Lesma, Faustine Bizet, Corentin Berardet, Nicolo Tonali, Sara Pellegrino, Myriam Taverna, Lucie Khemtemourian, Jean-Louis Soulier, Carine van Heijenoort, Frédéric Halgand, Tâp Ha-Duong, Julia Kaffy, Sandrine Ongeri

**Affiliations:** ^1^BioCIS, CNRS, Université Paris-Saclay, Châtenay-Malabry, France; ^2^Institute Galien Paris-Saclay, CNRS, Université Paris-Saclay, Châtenay-Malabry, France; ^3^DISFARM, Sezione di Chimica Generale e Organica “A. Marchesini,” Università degli Studi di Milano, Milan, Italy; ^4^Institute of Chemistry and Biology of Membranes and Nanoobjects, Institut Polytechnique Bordeaux, CNRS UMR 5248, Université de Bordeaux, Pessac, France; ^5^ICSN, Equipe Biologie et Chimie Structurales, Département de Chimie et Biologie Structurales et Analytiques, CNRS, Université Paris-Saclay, Gif-sur-Yvette, France; ^6^Institut de Chimie Physique, Equipe Chimie Analytique Physicochimie Réactivité des Ions, CNRS, Université Paris-Saclay, Orsay, France

**Keywords:** amyloid, hIAPP, type 2 diabetes, peptidomimetics, β-hairpin, aza-peptide, aggregation

## Abstract

Amyloid diseases are degenerative pathologies, highly prevalent today because they are closely related to aging, that have in common the erroneous folding of intrinsically disordered proteins (IDPs) which aggregate and lead to cell death. Type 2 Diabetes involves a peptide called human islet amyloid polypeptide (hIAPP), which undergoes a conformational change, triggering the aggregation process leading to amyloid aggregates and fibers rich in β-sheets mainly found in the pancreas of all diabetic patients. Inhibiting the aggregation of amyloid proteins has emerged as a relevant therapeutic approach and we have recently developed the design of acyclic flexible hairpins based on peptidic recognition sequences of the amyloid β peptide (Aβ_1–42_) as a successful strategy to inhibit its aggregation involved in Alzheimer’s disease. The present work reports the extension of our strategy to hIAPP aggregation inhibitors. The design, synthesis, conformational analyses, and biophysical evaluations of dynamic β-hairpin like structures built on a piperidine-pyrrolidine β-turn inducer are described. By linking to this β-turn inducer three different arms (i) pentapeptide, (ii) tripeptide, and (iii) α/aza/aza/pseudotripeptide, we demonstrate that the careful selection of the peptide-based arms from the sequence of hIAPP allowed to selectively modulate its aggregation, while the peptide character can be decreased. Biophysical assays combining, Thioflavin-T fluorescence, transmission electronic microscopy, capillary electrophoresis, and mass spectrometry showed that the designed compounds inhibit both the oligomerization and the fibrillization of hIAPP. They are also capable to decrease the aggregation process in the presence of membrane models and to strongly delay the membrane-leakage induced by hIAPP. More generally, this work provides the proof of concept that our rational design is a versatile and relevant strategy for developing efficient and selective inhibitors of aggregation of amyloidogenic proteins.

## Introduction

Over the past decades, the field of amyloidosis research has gained increasing interest due to its relationship to at least 20 serious human diseases. Most of them are related to aging and they are all related to improper folding and assembly of proteins into various aggregate structures which can interact with membranes and lead to the death of cells (Ke et al., 2017, 2020; Nguyen et al., 2021). Among the pathologies, the most in the spotlight are neurodegenerative diseases like Alzheimer’s Disease (AD), Parkinson’s Disease (PD), Huntington’s Disease, Prion Disease, or Amyotrophic Lateral Sclerosis (ALS). Nevertheless, other organs than brain can be affected such as pancreas and heart in Type 2 Diabetes (T2D). T2D is a major public health issue that entered the TOP 10 causes of death in the world and affect nearly 422 million people in the world, a number that is expected to increase to 600 million in 2045 (Chaudhury et al., 2017). The actual treatments aim to reduce hyperglycemia, but the disease continues to progress, and the loss of function and the death of pancreatic β-cells requires a gradual transition to insulin therapy. Although the mechanism responsible for β-cell dysfunction and death is only partially understood, amyloid deposits of human islet amyloid polypeptide (hIAPP, also known as amylin) are found in more than 95% of T2D patients, and more and more findings suggest an important role in pancreatic damage of the accumulation of misfolded β-sheet rich aggregates of hIAPP in the Langerhans islets (Jeong and An, 2015; Akter et al., 2016; Kiriyama and Nochi, 2018). The cell toxicity is closely linked to membrane disruption characterized by pore formation and aggregate formation-induced detergent-like mechanism but also to other suspected but poorly understood phenomena (Brender et al., 2012; Bram et al., 2014; Terakawa et al., 2018). Cardiovascular complications affect around 65% of T2D patients despite medication and hIAPP aggregates mediates cardiotoxicity (Despa et al., 2014; Rodriguez Camargo et al., 2018). Furthermore, T2D has been recognized as a risk factor for Alzheimer’s disease (AD) and cross-amyloid interactions between hIAPP and amyloid-β peptide (Aβ_1–42_) have been shown to play a critical role in AD due to an increased toxicity of Aβ-IAPP hetero-oligomerization on neuronal cell membranes (Roriz-Filho et al., 2009; Bharadwaj et al., 2020; Ly et al., 2021). A cross interaction between hIAPP and synuclein has been also suspected to also explain why patients with T2D are more likely to get Parkinson’s disease (Horvath and Wittung-Stafshede, 2016).

Thus, inhibiting the aggregation of hIAPP in order to prevent the formation of both large aggregates and highly toxic soluble oligomers has proven to be a valuable approach to be further explored to find a viable therapeutic strategy against T2D (Brender et al., 2012; Bram et al., 2014; Abedini et al., 2016; Kiriyama and Nochi, 2018). It might also decrease the risk factor of cardiovascular complications and of AD or PD in T2D patients.

Much fewer compounds have been reported as efficient inhibitors of hIAPP aggregation compared to Aβ_1–42_ inhibitors for AD. Small molecules such as non-selective polyphenols (Pithadia et al., 2016; Milardi et al., 2021) or di-phenyl pyrazole derivatives also able to inhibit the aggregation of several amyloid proteins (Saravanan et al., 2019) have been reported. Only few peptide derivatives (Gilead and Gazit, 2004; Cheng et al., 2012; Yan et al., 2013; Paul et al., 2017; Armiento et al., 2020) have been designed.

We recently demonstrated that peptide derivatives mimicking acyclic β-hairpin structures were very efficient to decrease Aβ_1–42_ aggregation and to preserve Aβ_1–42_ monomers (Pellegrino et al., 2017; Tonali et al., 2018). These compounds were built on a piperidine-pyrrolidine β-turn inducer and due to the fact that their design was based on self-recognition elements (SRE), i.e., selected sequences of Aβ_1–42_, they were selective for Aβ_1–42_ and not active on hIAPP aggregation. The next step is to demonstrate that this strategy can be generalized and applied to the inhibition of the aggregation of other amyloid proteins. The present work presents the rational pattern, the synthesis and the biophysical evaluations of new β-hairpin mimics, built on the piperidine-pyrrolidine β-turn, and designed to inhibit hIAPP aggregation. SREs based on hIAPP were selected and new compounds bearing peptidic sequences were proposed in combination with a novel strategy to decrease the peptidic character using a peptidomimetic arm of the azapeptide family (Chingle et al., 2017). In particular, original α/aza/aza/pseudotripeptides constituted by one natural amino acid and two consecutive aza-amino acids [abbreviated aza, in which the α–CH(R) of amino-acid residue is substituted by a nitrogen atom] were introduced. Complementary techniques were used to evaluate the overall conformation of the new structures (NMR and molecular modeling) and to attest the effect of this new class of peptidomimetics on both the early oligomerization steps (capillary electrophoresis and MS), and the overall fibrillization process (Thioflavin-T fluorescence assays and TEM). The capacity of the compounds to delay membrane leakage is also investigated. Pharmacomodulations were performed in order to establish some structure-activity relationships guidelines.

## Materials and Methods

### Synthesis and Characterization of the Compounds by NMR, HPLC, HRMS

Usual solvents were purchased from commercial sources, dried, and distilled by standard procedures. Pure compounds were obtained after liquid chromatography using Merck silica gel 60 (40–63 μm). TLC analyses were performed on silica gel 60 F250 (0.26 mm thickness) plates. The plates were visualized with UV light (λ = 254 nm) or revealed with a 4% solution of phosphomolybdic acid or ninhydrin in EtOH.

NMR spectra were recorded on a Bruker AMX 200 (^1^H, 200 MHz; ^19^F, 188 MHz), an ultrafield Bruker AVANCE 300 (^1^H, 300 MHz, ^13^C, 75 MHz), a Bruker AVANCE 400 (^1^H, 400 MHz, ^13^C, 100 MHz, ^19^F, 376 MHz), a Bruker AVANCE I 600 MHz (^1^H, 600 MHz, ^13^C, 150 MHz, ^19^F, 564 MHz) equipped with a z-gradient TCI and ^19^F-QCI, or a Bruker AVANCE III 800 MHz (^1^H, 800 MHz, ^13^C, 200 MHz) spectrometers, equipped with a z-gradient TCI cryoprobe.

Chemical shifts (δ) are in ppm and downfield from Me_4_Si (δ = 0.0 ppm) with the solvent resonance as the internal standard (^1^H NMR, CDCl_3_: δ = 7.26 ppm, CD_3_OD and CD_3_OH: δ = 3.31 ppm; ^13^C NMR, CDCl3: δ = 77.16 ppm, CD_3_OD and CD_3_OH: δ = 49.00 ppm). the following abbreviations are used: singlet (s), doublet (d), doublet of doublet (dd), triplet (t), quadruplet (q), multiplet (m), and broad singlet (bs).

Melting points were determined on a Kofler melting point apparatus. High-resolution mass spectra (HRMS) were obtained using a TOF LCT Premier apparatus (Waters), with an electrospray ionization source. The purity of compounds was determined by HPLC using a WATERS gradient system (pump + controller E 600, UV detector PDA 2996, autosampler 717) on a XSelect column (C18, 2.1 mm × 150 mm–3.5 μm), mobile phase, MeCN/H_2_O + 0.1% formic acid (gradient 5–100% in 20 min), detection at 257 nm. Preparative HPLC were performed on Agilent Infinity II.

Compounds **3–4**, **8–9**, and **14** were subjected to conformational studies by NMR in CD_3_OH. When signals were sufficiently resolved and disperse, vicinal ^3^*J*_*HN–H*α_ coupling constants, H^α^-HN ROE correlations, temperature coefficient (Δδ_*HN*_/ΔT) of the amide protons and ^1^H_α_ and ^13^C_α_ chemical shift deviations (CSD), were examined to analyze backbone conformational propensities.

All the protocols of synthesis and characterization data are given in Supplementary Material.

### Computational Methods

**9a** and **9b** three-dimensional structures were generated using the program MarvinSketch 6.2.1 from ChemAxon^1^. In both initial conformers, the torsion angle between the piperidine and proline (Cα-N-C3-C2) has a value of −60°, so that the proline Hα points toward piperidine H2, as observed in NMR experiments. The torsion angle around the amide bond between Leu-3 and Pip-4 (Cα-C-N-C2) was manually adjusted to −170° and −5° in **9a** and **9b**, respectively. Each conformer was placed in a cubic simulation box so that the minimum distance between the solute and the cube sides was 1.4 nm. Afterward, both simulation boxes were filled with explicit methanol molecules. Molecular dynamics simulations were performed by using the GROMACS 2019.1 package (Abraham et al., 2015) with the Generalized AMBER Force Field (GAFF; Wang et al., 2004) for both solute and methanol solvent. The length of the covalent bonds involving hydrogens was kept constant using the LINCS procedure (Hess, 2008). Lennard-Jones potentials were cut-off at 1.2 nm and electrostatic interactions were treated using the smooth PME method (Essmann et al., 1995). After two short simulations of 1 ns each to bring the system temperature and pressure around *T* = 300 K and *P* = 1 bar, each system was simulated during 200 ns in the isothermal-isobaric ensemble using the Nose-Hoover (Nosé, 1984; Hoover, 1985) and Parrinello-Rahman (Parrinello and Rahman, 1981) coupling methods. Molecular coordinates were saved every 20 ps for subsequent analyses by GROMACS tools, including *gmx angle* for extracting torsion angle values.

### Preparation of Large Unilamellar Vesicles

The LUVs were composed of a mixture of DOPC/DOPS in a 7:3 molar ratio. Stock solutions of DOPC and DOPS in chloroform at concentrations of 20–30 mM were mixed in a glass tube. The solvent was evaporated with dry nitrogen gas yielding a lipid film that was subsequently kept in a vacuum desiccator for 1 h. Lipid films were hydrated during at least 30 min in the 10 mM Tris, 100 mM NaCl buffer at pH 7.4. The lipid suspensions were subjected to 10 freeze-thaw cycles, at temperatures of approximately −190 and 50°C, respectively, and subsequently extruded 19 times through a mini-extruder (Avanti Alabaster, AL, United States) equipped with polycarbonate membranes (at 200 nm cut-off). Calcein-containing vesicles were obtained by adding 70 mM calcein to the hydration buffer and free calcein were removed from the LUVs using size exclusion chromatography (Sephadex G50-fine), using the hydration buffer (10 mM Tris, 100 mM NaCl, pH 7.4) to as the mobile phase. The phospholipid content of lipid stock solutions and vesicle preparations was determined by assessing inorganic phosphate according to Rouser (Rouser et al., 1970).

### Fluorescence-Detected ThT Binding Assay (hIAPP)

Human islet amyloid polypeptide, purchased from Bachem, was dissolved in pure hexafluoro-isopropanol (HFIP) at a concentration of 1 mM and incubated for 1 h at room temperature to dissolve any preformed aggregates. Next, HFIP was evaporated with dry nitrogen gas followed by vacuum desiccation for at least 3 h. The resulting peptide film was then dissolved in DMSO to obtain stock solutions of hIAPP (0.2 mM) and stock solutions of compounds to test were dissolved in DMSO (10, 1, and 0.1 mM). The concentration of DMSO was kept constant at 3% (v/v) in the final volume of 200 μL. Thioflavin-T binding assays were used to measure the formation of fibrils in solution or in the presence of membranes over time. A plate reader (Fluostar Optima, BMG Labtech) and standard 96-wells flat-bottom black microtiter plates in combination with a 440 nm excitation and 480 nm emission filters were used. The ThT assay was started by adding 5 μL of a 0.2 mM hIAPP stock solution to a mixture of 10 μM ThT (obtained from Sigma) and 10 mM Tris/HCl, 100 mM NaCl at pH 7.4 containing 1 μL of stock solutions of compound to test. For the experiments in the presence of membranes, the fluorescence assays were started by adding 5 μL of a 0.2 mM hIAPP (5 μM peptide) to 195 μL of a mixture of 10 μM ThT, DOPC/DOPS (7:3) LUVs, with a peptide:lipid ratio of 1:20. The concentration of IAPP was held constant at 5 μM for all experiments and inhibitors were added to yield compound/IAPP ratios of 10/1, 1/1, and 0.1/1 (only at ratio 1/1 for the assays in membrane). The ThT assays were performed in triplicate and between 2 and 4 times on different days, with the same batch of peptide. The ability of compounds to inhibit IAPP aggregation was assessed considering the time of the half-aggregation (*t1/2*) and the intensity of the experimental fluorescence plateau (F), both values were obtained by fitting the obtained kinetic data to a Boltzmann sigmoidal curve using GraphPad Prism 5. The relative extension/reduction of *t1/2* is defined as the experimental *t1/2* in the presence of the tested compound relative to the one obtained without the compound and is evaluated as the following percentage: [*t1/2* (hIAPP + compound) − *t1/2* (hIAPP)]/*t1/2* (hIAPP) × 100. The relative extension/reduction of the experimental plateau is defined as the intensity of experimental fluorescence plateau observed with the tested compound relative to the value obtained without the compound and is evaluated as the following percentage: (FhIAPP + compound – FhIAPP)/FhIAPP × 100. The curves of the tested compounds are fitted to a Boltzmann sigmoidal model, normalized to the control experiment.

Representative curves of ThT fluorescence assays over time showing hIAPP aggregation (5 μM) in the absence and in the presence of compounds **1**–**15** at compound/hIAPP ratios of 10/1, 1/1, and 0.1/1 are shown in Supplementary Material (Supplementary Figure 34). Representative curves of ThT fluorescence assays over time showing hIAPP aggregation (5 μM) in the presence of membranes, in the absence and in the presence of hairpins **8**, **10**, **13,** and **14** at compound/hIAPP ratios of 1/1 are shown in Supplementary Material (Supplementary Figure 35).

### Fluorescence-Detected ThT Binding Assay (Aβ_1–42_)

Aβ_1–42_ was purchased from Bachem and ThT was obtained from Sigma. The peptide was dissolved in an aqueous 1% ammonia solution to a concentration of 1 mM and then, just prior to use, was diluted to 0.2 mM with 10 mM Tris–HCl and 100 mM NaCl buffer (pH 7.4). Stock solutions of compounds to test were dissolved in DMSO with the final concentration kept constant at 0.5% (v/v). ThT fluorescence was measured to evaluate the development of Aβ1-42 fibrils over time using a fluorescence plate reader (Fluostar Optima, BMG Labtech) with standard 96-well black microtiter plates (final volume in the wells of 200 μL). Experiments were started by adding the peptide (final Aβ1-42 concentration equal to 10 μM) into a mixture containing 40 μM ThT in 10 mM Tris–HCl and 100 mM NaCl buffer (pH 7.4) with and without the compounds at different concentrations (100, 10, 1 μM) at room temperature. The ThT fluorescence intensity of each sample (performed in triplicate) was recorded with 440/480 nm excitation/emission filters set for 42 h performing a double orbital shaking of 10 s before the first cycle. The fluorescence assays were performed between 2 and 4 times on different days, with the same batch of peptide. The ability of compounds to inhibit Aβ1-42 aggregation was assessed considering the time of the half-aggregation (*t1/2*) and the intensity of the experimental fluorescence plateau (F), both values were obtained by fitting the obtained kinetic data to a Boltzmann sigmoidal curve using GraphPad Prism 5. The relative extension/reduction of *t1/2* is defined as the experimental *t1/2* in the presence of the tested compound relative to the one obtained without the compound and is evaluated as the following percentage: [*t1/2* (Aβ + compound) − *t1/2* (Aβ)]/*t1/2* (Aβ) × 100. The relative extension/reduction of the experimental plateau is defined as the intensity of experimental fluorescence plateau observed with the tested compound relative to the value obtained without the compound and is evaluated as the following percentage: (FAβ + compound – FAβ)/FAβ × 100. Curves of the tested compounds are fitted to a Boltzmann sigmoidal model, normalized to the control experiment and represented in Supplementary Material.

Representative curves of ThT fluorescence assays over time showing Aβ_1–42_ (10 μM) aggregation in the absence and in the presence of **1–3, 8,** and **14** at compound/Aβ_1–42_ ratios of 10/1, 1/1, and 0.1/1 are shown in Supplementary Material (Supplementary Figure 36).

### Transmission Electron Microscopy

Samples were prepared under the same conditions as in the ThT-fluorescence assay. Aliquots of hIAPP (5 μM in 10 mM Tris–HCl, 100 mM NaCl, pH 7.4 in the presence and absence of hairpins **3**, **8**, and **14** were adsorbed onto 300-mesh carbon grids for 2 min, washed and dried). The samples were negatively stained for 45 s on 2% uranyl acetate in water. After draining off the excess of staining solution and drying, the grids were observed using a JEOL 2100HC TEM operating at 200 kV with a LaB6 filament. Images were recorded in zero-loss mode with a Gif Tridiem energy-filtered-CCD camera equipped with a 2 k × 2 k pixel-sized chip (Gatan, Inc., Warrendale, PA, United States). Acquisition was accomplished with the Digital Micrograph software (versions 1.83.842, Gatan, Inc., Warrendale, PA, United States).

### Membrane Permeability Assay

Leakage experiments were performed in standard 96-wells transparent microtiter plates using a plate reader (Spectrafluor, Tecan, Salzburg, Austria). Aliquots of 2.5 μL of molecules solution in DMSO at the desired concentration were added to 192.5 μL of 100 μM calcein-containing lipid vesicles in 10 mM Tris–HCl, 100 mM NaCl buffer at pH 7.4. The assay was then started by adding 5 μL of a 0.2 mM IAPP solution in DMSO or 2.5 μL DMSO only as control. Directly after addition of all components, the microtiter plate was shaken for 10 s. The plate was not shaken during the measurement. Fluorescence of calcein was measured from the top, every 5 min, using a 485 nm excitation filter and a 535 nm emission filter. The temperature during the measurement was 25 ± 3°C. The maximum leakage at the end of each measurement was determined by adding 2 μL of 10% Triton X-100 to a final concentration of 0.1% (v/v). The release of fluorescent dye was calculated according to the following equation:


(1) L(t)=(Ft--F0)/(F100--F0)

L(t) is the fraction of dye released (normalized membrane leakage) at time t, Ft is the measured fluorescence intensity at time t, and F0 and F100 are the fluorescence intensities at times *t* = 0 and after addition of Triton X-100, respectively. All membrane leakage assays were performed three times, each in triplicate, on different days, using different IAPP stock solutions.

### Capillary Electrophoresis

Sample preparation: commercial hIAPP was dissolved upon reception in pure HFIP at 1 mM and incubated for 1 h at room temperature, followed by an aliquoting and an immediate evaporation under nitrogen, then under vacuum and storage at −20°C. Just before performing real time kinetics, the dried peptide was reconstituted in 50 mM ammonium acetate buffer pH 3.7 at 100 μM. Compounds **3**, **8,** and **14** were dissolved in DMSO and added to the peptide solution to reach final concentrations 1 mM, 100 and 50 mM, corresponding, respectively, to ratios 10/1, 1/1, and 0.5/1 of compounds:hIAPP; the final concentration of DMSO in the ammonium acetate buffer being of 1% (v/v).

CE-UV experiments were carried out with a MDQ Instrument (SCIEX, Framingham, MA, United States) equipped with a UV detector. Detection was performed at 200 nm. Fused silica capillaries (50 μm id × 365 μm od) were purchased from Polymicro Technologies (Phoenix, AZ, United States). Silica capillaries were coated with a 0.2% polybrene solution. Briefly, solid polybrene was dissolved in water under heating at 50°C. The capillary was preconditioned with MeOH, NaOH 1 M, NaOH 0.1 M, and with H_2_O. All flushings were done at 20 psi for 15 min. The capillary was then coated with the 0.2% polybrene solution for 15 min at 20 psi. The coated capillary was then flushed with the background electrolyte (BGE) at 20 psi for 45 min and equilibrated under −25 kV for 2 h. Polybrene-coated capillaries were 60 cm total length (49.8 cm to the detector). The BGE was a 50 mM ammonium acetate buffer, pH 3.7. The separation was carried out under −25 kV at 25°C. Samples were injected from the inlet by hydrodynamic injection at 0.8 psi for 10 s. After each run, the capillary was rinsed for 5 min with 1 M NaOH, 5 min with water, recoated for 10 min with 0.2% polybrene, and finally equilibrated with the running buffer for 5 min at 20 psi.

Since the control hIAPP kinetics may slightly vary depending on external features (such as the use of different hIAPP aliquots, colder or warmer room temperature), the evaluation of each hairpin was carried out simultaneously with the hIAPP control experiment to avoid any bias.

#### ESI-MS Experiments

ESI-MS experiments were carried out with a Synapt G2-S^*i*^ Q-TOF instrument (Waters, Manchester, United Kingdom) equipped with an ESI interface. Positive ion mode was used. Samples of hIAPP (as previously described for the CE-UV experiments) were injected by direct infusion through a syringe at 5 μL/min. Main MS parameters are detailed the following: the capillary voltage was set at 2.4 kV, with the sampling cone and source offset at 70 V. Source and desolvation temperatures were, respectively, 40 and 75°C. Data were processed with MassLynx.

## Results

### Design

**FIGURE 1:**
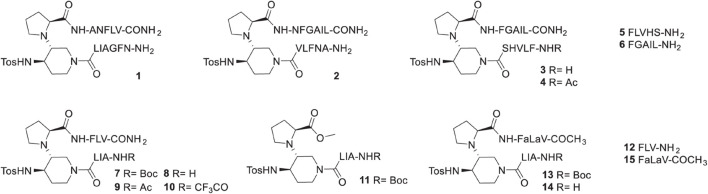
Structure of the synthesized compounds **1**–**15**.

As explained above, we designed peptides derivatives mimicking acyclic β-hairpin structures selective for hIAPP. Our choice for the first SRE to link to the piperidine-pyrrolidine β-turn inducer, turned on the amyloidogenic sequence N_22_FGAIL_27_ because this region is strongly involved in hIAPP aggregation process and in the folding of hIAPP described in experimental and *in silico* models of monomers and aggregates found in the literature (Sumner Makin and Serpell, 2004; Kajava et al., 2005; Luca et al., 2007; Wiltzius et al., 2008; Bedrood et al., 2012; Liang et al., 2013; Wu and Shea, 2013; Tran and Ha-Duong, 2016; Cao et al., 2020; Röder et al., 2020). Furthermore, this sequence has been reported to be able to promote hIAPP fibrillization (Porat et al., 2004) and to inhibit its aggregation when inserted in a macrocycle peptide derivative (Buchanan et al., 2013). More recently, a series of hIAPP-derived 21-residues peptides, based on this hot segment FGAIL sequence, was designed by the group of Kapurniotu and reported as nanomolar inhibitors of hIAPP and Aβ140 self-assembly (Andreetto et al., 2015; Niu et al., 2020). Selecting the second SRE was trickier because the sequences facing N_22_FGAIL_27_ vary from one model to another being slightly shifted within the sequence A_13_ to S_20_. The selection of the complementary SRE in compounds **1** and **2** was based on models reported by Kajava et al. (2005), Andreetto et al. (2015) and Mirecka et al. (2016), where A_13_NFLV_17_ is facing N_22_FGAIL_27_. The only difference lies in the inversion of the SRE position with NFGAIL at the *N*-terminus in **1** or at the *C*-terminus in **2** (Figure 1). Compounds **3** and **4** (Figure 1) were based on models reported by Wiltzius et al. (2008), Liang et al. (2013), and Wu and Shea (2013) where F_15_LVHSS_20_ is facing N_22_FGAIL_27_. We kept five residues in each sequence, as pentapeptide sequences were sufficient in our hairpin mimics inhibiting Aβ_1–42_ (Pellegrino et al., 2017). On the contrary to Aβ_1–42_, hIAPP does not present acidic residue in the amyloidogenic region with which the terminal amine of the inhibitor would establish interesting ionic interactions. Thus, we introduced in compound **4** an *N*-acetyl group. The isolated SREs F_15_LVHS_19_ **5** and F_23_GAIL_27_ **6** (Figure 1) were also prepared to demonstrate the interest of inserting the SREs in β-hairpin mimics. The next step was to decrease the peptidic character in the β-hairpin mimics. We kept the sequences A_25_IL_27_ and F_15_LV_17_ that were present in compounds **1–3**. The terminal amine was either free or protected by three different groups: Boc, acetyl or trifluoroacetyl (compounds **7**–**10**). The free amine should increase the water solubility but is probably not mandatory for the interaction with hIAPP, as described above for **4**. The trifluoroacetyl group was selected to examine how the presence of fluorine atoms could influence the activity. Fluorine possesses the unique capacity to increase the local hydrophobicity, to strengthen hydrogen bonds and to increase metabolic stability, that have been exploited in more than 300 approved drugs but not yet in peptide pharmaceuticals (Inoue et al., 2020). Fluorine has been also recently demonstrated as a useful probe for studying biological events by ^19^F-NMR spectroscopy (Dalvit and Vulpetti, 2019). The shorter compounds **11** and **12** were also evaluated to validate the interest of linking two peptide arms to the piperidine-pyrrolidine β-turn inducer. Finally, one α/aza/aza/pseudotripeptide, Phe-azaLys-azaVal-COCH_3_ (abbreviated as FaLaV-COCH_3_) mimicking the *C*-terminal arm FLV of **8**, was inserted in compounds **13** and **14** and evaluated alone (compound **15**). The introduction of one aza-amino acid in peptides has shown significant success in providing biologically active peptides (see the references cited in Tonali et al., 2020). However, to our knowledge, only scarce examples of biologically active peptide mimics having two consecutive aza-amino acids have been reported (Gante et al., 1995; Han et al., 1998). We ourselves introduced a diazaGly motif (Dufau et al., 2012) in inhibitors of HIV-1 protease dimerization. Here, we want to assess whether the diaza-amino acid motif can retain the selectivity of natural peptides due to side chains.

### Synthesis

**SCHEME 1:**
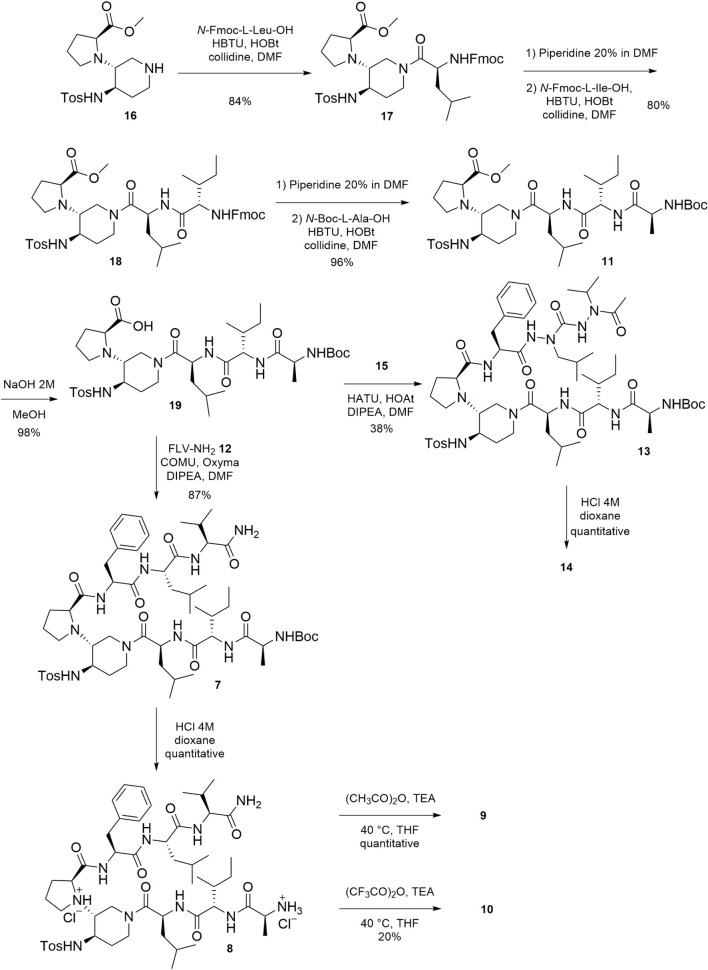
Synthesis of compounds **7**–**11**, **13**, **14**.

**SCHEME 2:**
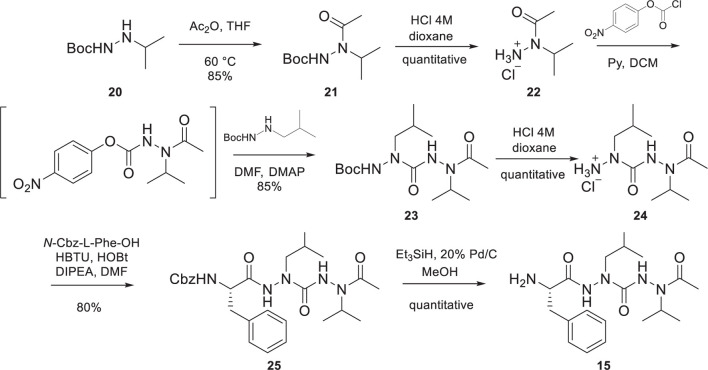
Synthesis of the peptidomimetic arm FaLaVCOCH_3_ **15**.

The preparation of the β-hairpin mimics **1**–**4** and of the SREs **5** and **6,** was performed by solid phase peptide synthesis, using the Fmoc strategy on a rink amide resin, as previously reported by some of us (Pellegrino et al., 2017). The solution synthesis, outlined in Scheme 1, allowed the preparation of the target compounds **7–14** and start from scaffold **16** that was prepared in accordance with our published procedure (Pellegrino et al., 2014, 2017). To the *N*-terminus of **16**, the three amino acids *N*-Fmoc-L-Leu-OH, *N*-Fmoc-L-Ile-OH, and *N*-Boc-L-Ala-OH were successively coupled to form the tripeptide AIL, in order to obtain the key intermediate **11**. The coupling reagents HBTU/HOBt in the presence of collidine in dry DMF allowed the couplings in very satisfactory yields (80–96%, Scheme 1). The saponification of the methyl ester of **11**, using NaOH 2 M in MeOH, afforded **19** in an excellent yield (96%). Its coupling with the tripeptide FLV-NH_2_ **12** (synthesized in good yield, see experimental section), using COMU/Oxyma as coupling agent, in the presence of DIPEA in dry DMF, led to **7** in a very good yield (87%). Then, a classical acidic cleavage of the Boc moiety of **7** gave **8** in quantitative yield. *N-*acylation of **8** using acetic anhydride or trifluoroacetic anhydride, provided **9** and **10,** respectively. The coupling between the intermediate **19** and the peptidomimetic arm FaLaVCOCH_3_ **15**, (whose synthesis is explained just below and in Scheme 2) resulted more difficult than in the case of the coupling with the peptide FLV-NH_2_ **12**. The desired compound **13** was obtained in satisfactory yield (59%), together with a compound resulting from the intramolecular cyclization between the carboxylic group and the NH of the sulphonamide of **19** (Supplementary Figure 28). The formation of the cyclized by-product only observed in the coupling with the di-aza-tripeptide could be due to its steric hindrance and/or lower reactivity. Finally, the acid cleavage of the Boc moiety of **13** afforded the final compound **14** in quantitative yield.

The preparation of the di-aza-tripeptide FaLaVCOCH_3_ **15**, reported in Scheme 2, required the combination of hydrazine chemistry and peptide synthesis. As recently reported by our group (Bizet et al., 2018), the introduction of two sequential aza-amino acids can be challenging because of the capricious reactivity of the alkyl-hydrazine. In our case, it was possible to prepare the di-aza-tripeptide arm **15** by elongating the peptidomimetic from the *C-*terminus to the *N-*terminus. Acetylation of Boc-2-isopropyl-hydrazine (**20**) prepared according to our reported procedure (Bizet et al., 2018), afforded **21** in a good yield (85%). The classical acidic cleavage of the Boc moiety of **21** gave **22**. The activation of **22** using 4-nitrophenyl chloroformate, followed by the reaction with Boc-2-isobutyl-hydrazine led to the Boc-protected intermediate **23** in a very satisfactory yield (85%). The acidic cleavage of the Boc moiety of **23** followed by the coupling reaction of the intermediate **24** with *N*-Cbz-L-Phe-OH gave **25** in very good yield (80%). The cleavage of the Cbz moiety of **25** to afford the desired FaLaVCOCH_3_ **15** was achieved employing McMurray’s hydrogenolysis (triethylsilane in the presence of Pd/C in MeOH (Mandal and McMurray, 2007).

### Conformational Studies

In order to check if the conformational behavior of our new peptidomimetics was similar to the one of our previously reported acyclic β-hairpin structures bearing SREs based on Aβ_1–42_ (Pellegrino et al., 2017; Tonali et al., 2018), we performed conformational studies by NMR on the most active inhibitors of hIAPP aggregation **3–4**, **8–9**, and **14**, as assessed by ThT fluorescence assay (see below). The polar and protic solvent CD_3_OH was used instead of water because of the low solubility in water of some compounds. Another important advantage of CD_3_OH is the possibility to record NMR spectra at low temperatures (248–273 K range), thus allowing to better characterize slowly interconverting conformers. When signals were sufficiently resolved and disperse, vicinal ^3^*J*_*HN–H*α_ coupling constants, H^α^-HN ROE (Rotating-frame Overhauser Effect) correlations, temperature coefficient (Δδ_*HN*_/ΔT) of the amide protons and ^1^H_α_ and ^13^C_α_ chemical shift deviations (CSD), were examined to analyze backbone conformational propensities (see Supplementary Material for detailed data).

Compounds **3** and **4** bearing five residues sequences adopt similar β-hairpin like structure observed in our similar peptide compounds (Pellegrino et al., 2017) as confirmed by a good dispersion of the NH chemical shifts, sequential CHα_*i*_/NH_*i+*1_ ROEs for the extended peptide arms and characteristic ROE proximities in the beta-turn region and between the two strands (see Supplementary Material).

**FIGURE 2:**
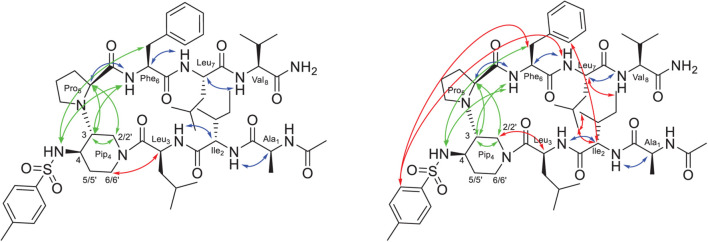
Structure of conformers **9a (left)** and **9b (right)** showing the assigned ROEs: in blue the sequential CHα_*i*_/NH_*i+1*_ ROEs, in green the common ROEs, and in red the differences.

Conversely, compounds **8** and **9**, bearing two shorter tripeptide arms, displayed the presence of at least two major conformers in dynamic equilibrium. Complete assignment of the ^1^H and ^13^C signals was performed for the two conformers of compound **9** at 278 K (Supplementary Tables 6, 7). At this temperature, an equilibrium between two major conformers (ratio 1:1, **9a**/**9b**) was observed. Large vicinal ^3^JNH-H_α_ constants (>8.0 Hz) were observed in both cases, suggesting the presence of dihedral angles typical of peptide segments in extended conformation. Several sequential CHα_*i*_/NH_*i+*1_ ROEs, indicating β-conformations, were found for both **9a** and **9b** conformers (Figure 2). The extended conformation of the two peptide arms was assumed by the positive difference between experimental Hα and HN chemical shift values and “random ones” (CSDs) (Supplementary Tables 8, 9). The very slight differences in values of ^3^JNH-Hα and of CSD let consider that the peptide arms were similarly extended in both conformers **9a** and **9b**. ROESY experiments confirmed the presence of the β-turn in both conformers (for details, see Supplementary Material). However, we detected inter-strand diagnostic ROEs only for **9b** isomer (Figure 2), confirming spatial proximity between the two peptide arms. In particular, ROE contacts were visible between the Hα of Ile-2 and the aromatic ring of Phe-6 and the CH_3_ of Leu-7, between the Hα of Leu-7 and the CH_3_ of Ile-2 and, finally, between the side chains of Ile-2 and Leu-7 (Figure 2 right and Supplementary Figure 23). This difference in ROEs let us hypothesize a dynamic equilibrium between two different β-hairpin architectures, with only one of them allowing the spatial proximity between the peptide arms. This difference between the two conformers also results in a different ROE contact between the Hα of Leu-3 and the diastereotopic protons H2 or H6 of Pip-4. In **9a**, Leu-3 Hα is closed to H6, while in **9b** Leu-3 Hα provides a ROE with H2 (Figure 2 and Supplementary Figure 21).

We decided to perform a molecular dynamics (MD) study to investigate if a different torsion angle of the amide bond between Leu-3 and Pip-4 could explain the two different β-hairpin architectures observed experimentally.

**FIGURE 3:**
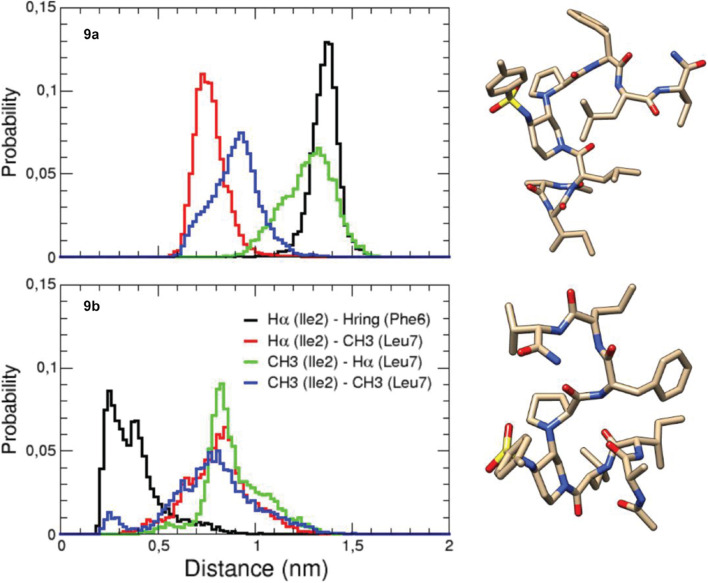
MD-derived probability distribution of distances between protons in each of the two peptide arms. On the right are displayed representative structures of the most populated clusters of **9a** and **9b**.

200 ns-MD simulations of both conformers **9a** and **9b** were performed in explicit MeOH solvent at 280 K in order to be as close as possible to experimental conditions. In both initial structures, the torsion angle between the piperidine and proline rings was identical with proline Hα pointing toward piperidine H2 (torsion angle Cα-N-C3-C2 around −60°), as found in NMR analysis. The main difference between the two initial structures was the torsion angle around the amide bond between Leu-3 and Pip-4 (Figure 2): the Cα-C-N-C2 angle was equal to −170° and −5° in the initial structures **9a** and **9b**, respectively. The piperidine-proline local structure was maintained and no conformational transition of the corresponding initial torsion angle Cα-C-N-C2 between Leu-3 and Pip-4 occurred in MD simulations, for both conformers **9a** and **9b** (see section “Discussion” and Supplementary Figure 24). The computed ^3^J_*HN–H*α_ coupling constants had lower values than NMR data, indicating that the two peptide arms were slightly less extended in MD simulations than in NMR experiments (see section “Discussion” and Supplementary Table 14). Interestingly, as observed in our NMR studies, significant differences in the spatial proximity of the two peptide arms were observed between modeled conformers **9a** and **9b**. Indeed, when analyzing the probability distribution of the distances between protons for which ROEs were detected (Figure 3), the distance between Ile-2 Hα and Phe-6 aromatic ring as well as that one between Ile-2 side chain CH_3_ and Leu-7 Hα were clearly lower in **9b** than in **9a**. This indicates that the two peptide arms are closer in conformer **9b** than in **9a** (Figure 3). These results confirm that the conformation of the torsion angle Cα-C-N-C2 between Leu-3 and Pip-4 impacts the spatial proximity of the two peptide arms and the global β-hairpin architecture. When Leu-3 Hα is oriented toward piperidine H6 (**9a**), this promotes a separation of the two peptide arms, whereas when it is oriented toward piperidine H2 (**9b**), the two arms can approach one to the other.

This conformational analysis by NMR and molecular dynamics, performed on the acetylated hairpin mimic **9**, allowed us to establish some general guidelines, useful for the structural characterization of similar compounds based on our β-turn inducer when NMR assignment was not as precise as for compound **9**. In the case of the free *N*-terminal compound **8**, it was not possible to perform a complete assignment of the two major conformers in equilibrium, even by lowering the temperature at 278 K. By increasing the temperature at 313 K, we could approach an approximately complete coalescence of the two conformers, allowing the attribution of an average conformation with a complete ^1^H and ^13^C assignment (Supplementary Table 3). The good dispersion of the NH chemical shifts indicated the presence of a single and major conformation whose turn structure was confirmed by ROESY experiments. Several sequential CHα_*i*_/NH_*i+*1_ ROEs, large ^3^*J*_*NH–H*α_ coupling constants and positive ^1^H CSD values (Supplementary Table 4) for most of the amino acids (less remarkable for the more dynamic *N*-terminal Ala and *C*-terminal Val), suggested the extended conformation of the two peptide arms. Diagnostic inter-strand ROEs were observed between the NH of Leu-3 and the Hα and Hβ of Phe-6 and were in favor of a β-hairpin architecture (Supplementary Figures 13, 14). No one of the amide protons showed a temperature coefficient lower than −4.5 ppb K^–1^ (Supplementary Table 5). However, Ile-2, Leu-3, and Leu-7 values fell within intermediate values, suggesting their partial involvement in hydrogen-bond. Taking together all these experimental results, it is possible to conclude that at 313 K, the average conformation of **8** adopts a partial β-hairpin conformation, with the *N*-terminal Ala residue not in contact with the C-terminal acetyl amide group, contact that would have allowed to form a more extended β-hairpin.

The ^1^H and ^13^C assignment of compound **14** was tricky even performed on a spectrometer operating at 800 MHz and equipped with a TCI cryoprobe. A dispersion of NH chemical shifts and poor resolution of the signals indicated the presence of different conformers whatever the temperature from 278 to 313 K. Some signals and ^3^*J*_*NH–H*α_ coupling constants of the natural amino acids could not be determined with certainty (Supplementary Table 15). The temperature dependence of amide proton chemical shifts of Ile-2 was intermediate (−4.5 ppb K^–1^, Supplementary Table 16), suggesting its partial involvement in hydrogen-bond. Several sequential CHα_*i*_/NH_*i+*1_ ROEs and large and positive ^1^H CSD values (Supplementary Table 17) for Ile, Leu, and Phe confirmed the extended conformation of the natural amino acids in the two arms (Supplementary Figure 30). No typical spatial proximities were found between H_α_ Pro-5 and Pip-4 and between the Pip-4 and NH Phe-6 to confirm the turn structure. Moreover, it can be affirmed that an equilibrium between two different β-hairpin architectures, one of which more “open” than the other, occurs even for compound **14**, reflecting the same behavior as conformers **9a** and **9b**. A very weak inter-strand ROE was observed and attributed to Phe-6 Hδ and Leu-3 Hδ or Ile-2 Hγ whose chemical shifts were identical (Supplementary Figure 31). Overall, **14** seems to adopt a more flexible β-hairpin structure than the peptide analogs **8** and **9**.

### Evaluation of the Effect of the Synthesized Compounds on hIAPP Aggregation

#### Inhibition of hIAPP Fibrillization by Thioflavin-T (ThT) Fluorescence Assay

**FIGURE 4:**
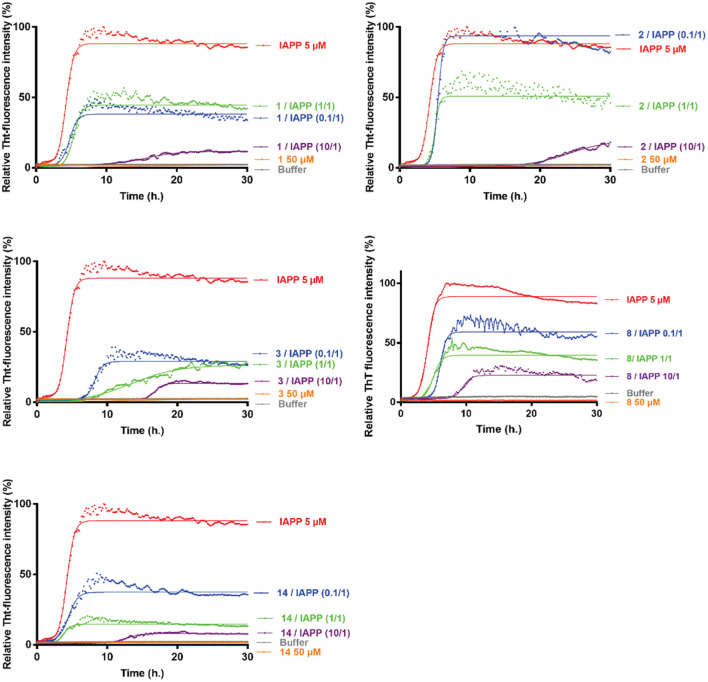
Kinetics of amyloid fibrils formation. Representative curves of ThT fluorescence assays over time showing hIAPP aggregation (5 μM) in the absence (red curve) and in the presence of compounds **1**–**3**, **8,** and **14** at compound/hIAPP ratios of 10/1 (purple curves), 1/1 (green curves) and 0.1/1 (blue curves). The control curves are represented in orange lines and buffer in gray.

The evaluation on hIAPP fibrillization was performed for the hairpin **1**–**4**, **7**–**10, 13**–**14,** the intermediate **11** and the arms **5**–**6**, **12**, **15** employing Thioflavin-T (ThT) fluorescence assay (representative curves are shown in Figure 4 and Supplementary Figure 34). ThT is a dye able to fluoresce upon binding to β-sheet rich structures (LeVine, 1999) and it is the standard dye used to study the aggregation kinetics of amyloidogenic proteins (Gade Malmos et al., 2017). The aggregation curve of hIAPP alone displays the classical sigmoidal pattern, characterized by three different portions: an initial lag phase of about 1 h, followed by a rapid elongation phase which ends to a final plateau reached after about 5 h (red curves, Figure 4). From the curve it is possible to collect two parameters: (1) F, maximal fluorescence measured at the plateau which is related to the highest amount of the formed fibrils (2) *t_1/2_*, the time at which the fluorescence has reached the half of F. The inhibitory activity was evaluated at three different compound/hIAPP ratios: 10/1, 1/1, and 0.1/1; the calculated F and *t*_1/2_ are summarized in Table 1.

**TABLE 1:** Effects of compounds **1**–**15** on hIAPP fibrillization assessed by ThT-fluorescence spectroscopy at 10/1, 1/1, and 0.1/1 ratios of compound/hIAPP.

Compound	Compound/hIAPP ratio^[a]^	*t*_1/2_ extension/reduction (%)^[b]^	F extension/reduction (%)^[c]^
**1**	(10/1)	+230 ± 20%	−89 ± 2%
	(1/1)	+27 ± 14%	−49 ± 28%
	(0.1/1)	+15 ± 8%	−57 ± 18%
**2**	(10/1)	+441 ± 64%	−76 ± 5%
	(1/1)	+27 ± 14%	−44 ± 8%
	(0.1/1)	+37 ± 9%	+22 ± 13%
**3**	(10/1)	+280 ± 65%	−87 ± 2%
	(1/1)	+166 ± 32%	−72 ± 3%
	(0.1/1)	+88 ± 47%	−78 ± 8%
**4**	(10/1)^[d]^	+72 ± 16%	−73 ± 3%
	(1/1)	+82 ± 4%	−54 ± 7%
	(0.1/1)	+56 ± 25%	−46 ± 5%
**5**	(10/1)^[d]^	ne^[e]^	−59 ± 10%
	(1/1) 5	ne	−44 ± 31%
	(0.1/1) 0.5	ne	−48 ± 9%
**6**	(10/1)	ne	−46 ± 10%
	(1/1)	ne	ne
	(0.1/1)	ne	ne
**7**	(10/1) ^[d]^	−10 ± 3%	−57 ± 12%
	(1/1)	−13 ± 1%	−70 ± 5%
	(0.1/1)	ne	−37 ± 9%
**8**	(10/1)	+147 ± 24%	−72 ± 8%
	(1/1)	+45 ± 6%	−62 ± 6%
	(0.1/1)	+64 ± 2%	−36 ± 2%
**9**	(10/1) ^[d]^	ne	−65 ± 6%
	(1/1)	ne	−67 ± 14%
	(0.1/1)	ne	−51 ± 3%
**10**	(10/1) ^[d]^	ne	−67 ± 5%
	(1/1)	ne	−60 ± 10%
	(0.1/1)	ne	ne
**11**	(10/1)	+55 ± 6%	−52 ± 8%
	(1/1)	ne	−44 ± 1%
	(0.1/1)	nd^[f]^	nd
**12**	(10/1)	+25 ± 9%	−19 ± 4%
	(1/1)	ne	−58 ± 9%
	(0.1/1)	−13 ± 2%	+27 ± 10%
**13**	(10/1) ^[d]^	ne	−88 ± 1%
	(1/1)	ne	−76 ± 4%
	(0.1/1)	ne	−48 ± 7%
**14**	(10/1)	+186 ± 11%	−93 ± 2%
	(1/1)	ne	−85 ± 1%
	(0.1/1)	ne	−60 ± 2%
**15**	(10/1)	ne	ne
	(1/1)	ne	ne
	(0.1/1)	ne	ne

*Parameters are expressed as mean ± SE, n = 3. ^[a]^Compounds were dissolved in DMSO; the final concentration kept constant at 3% (v). The concentration of hIAPP was 5 μM. ^[b]^See Supplementary Material for the calculation of the t_1/2_ extension/reduction. ^[c]^See Supplementary Material for the calculation of the F extension/reduction. ^[d]^Compounds **4, 5, 7, 9, 10,** and **13** self-aggregate at 50 μM, ^[e]^ne, no effect, ^ [f]^nd, not determined.*

The longer hairpins **1–4** displayed significant and similar inhibitory effect on hIAPP fibrillization at the three tested ratios with a dose-dependent effect. Hairpin **1** showed only a slightly more pronounced activity on the fluorescence plateau compared to **2** (ΔF = −89, −49, −57% vs. −76, −44, +22% for **1** and **2,** respectively, at 10/1, 1/1, and 0.1/1 ratios), however, hairpin **2** exhibited a significant higher activity in delaying aggregation, in particular at the highest ratio 10/1 (Δ *t*_1/2_ = +441% for **2** vs. +230% for **1**). For both hairpins **1** and **2**, the activity on *t*_1/2_ was more modest at 1/1 and 0.1/1 ratios (+15% <Δ *t*_1/2_< +37%). Hairpin **3**, bearing the two pentapeptidic arms F_15_LVHS_19_ facing F_23_GAIL_27_, showed a comparable activity to **1** at a 10-fold excess (Δ *t*_1/2_ = +280% vs. +230% and ΔF = −87% vs. −89% for **3** and **1,** respectively). Remarkably, hairpin **3** has proven to be the most potent inhibitor in this series since its activity on fibrillization kinetics was still significative at the low 1/1 and 0.1/1 ratios (Δ *t*_1/2_ = +166, +88% and ΔF = −72, −78% at 1/1 and 0.1/1 ratios, respectively). Hairpin **4** which is the *N*- acetylated analog of **3**, was significantly less active in particular in delaying the fibrillization (+72% <Δ *t*_1/2_< +56% for **4** compared to +280% <Δ *t*_1/2_< +88% for **3** at the three tested ratios). These results suggested that the position of the sequence F_23_GAIL_27_ at the *C*-terminus, its alignment with the sequence F_15_LVHS_19_, and the free *N*-terminus in hairpin **3** are three beneficial elements to reach a good effect on delaying the aggregation kinetics and decreasing the amount of hIAPP fibrils, even at low 1/1 and 0.1/1 (**3**/hIAPP) ratios. On the contrary, minimal to no activity was observed for the isolated SREs, FGAIL (**5**) and FLVHS (**6**), demonstrating that these SREs are effective only when they are inserted in the whole β-hairpin structure.

Pleasantly, the hairpin **8**, bearing two shorter tripeptidic arms, retained most of the activity. Indeed, the free amine **8** significantly delayed the fibrillization at 10/1 ratio (Δ *t*_1/2_ = +147%, ΔF = −72%) but to a lesser extent than hairpins **1**–**3**. Nevertheless, at the low 1/1 and 0.1/1 ratios, the activity on hIAPP fibrillization was maintained and comparable to slightly better than for hairpins **1** and **2** (Δ *t*_1/2_ = +45, +27, +27% for **8**, **1,** and **2,** respectively; ΔF = −62, −49, −44% for **8**, **1,** and **2,** respectively, at 1/1 ratio). Here also the protection of the *N*-terminal amine was deleterious. Even if Boc-protected (**7**), acetylated (**9**), and trifluoroacetylated (**10**) compounds retained some activity on the fluorescence plateau comparable to the free amine hairpin **8** (−57% <ΔF<−67% at 10/1 ratio and −60% <ΔF<−70% at 1/1 ratio), however, they were totally inefficient on the kinetics of hIAPP fibrillization. The Boc protected analog **7** induced a very slight acceleration of the aggregation process (Δ *t*_1/2_ = −10 and −13%, respectively, at 10/1 and 1/1 ratios). The lower efficiency of the three protected hairpins **7**, **9,** and **10** could be linked to their tendency to self-aggregate.

Interestingly, the hairpin of lower peptidic character **14**, bearing one diaza-tripeptide arm exceeded the delay activity of its peptide analog **8** at the highest ratio 10/1 (Δ *t*_1/2_ = +186% vs. +160% for, respectively, **14** and **8**) but no effect was detected on the fibrillization rate at 1/1 and 0.1/1 ratios. Nevertheless, an important reduction of the fluorescence plateau was observed whatever the concentration tested (ΔF = −93, −85, −60% for 10/1, 1/1, and 0.1/1 ratios), this decrease being more pronounced compared to that observed for the peptide analog **8** (ΔF = −72, −62, −36% for 10/1, 1/1, and 0.1/1 ratios), and being the most effective among all the evaluated compounds. As observed for the peptide analogs **7** and **8**, the activity of the *N*-terminal Boc-protected hairpin **13** was lower; no effect was observed on the *t*_1/2_ while the effect on the fluorescence plateau was maintained but slightly lower than for **14** at each ratio (ΔF = −88, −76, −48% vs. −93, −85, −60% for respectively, **13** and **14** at 10/1, 1/1, and 0.1/1 ratios). Finally, to confirm that the entire β-hairpin structure was essential to modulate hIAPP fibrillization, the truncated molecule **11**, the peptide arm FLV **12** and the diaza-pseudopeptide arm **15** were also evaluated by the ThT-fluorescence assay. The intermediate **11**, composed of the scaffold linked to only one tripeptide arm AIL, retained some activity (Δ *t*_1/2_ = +55%, ne and ΔF = −52, −44% for 10/1 and 1/1 ratios), but nevertheless much weaker than the full peptide (**8**) and peptidomimetic (**14**) hairpins. The peptide (**12**) and the diaza-tripeptide (**15**) arms were almost to completely inactive, respectively.

These results clearly indicate that the peptidic character can be lowered by decreasing the length of the peptide arms and by inserting an α/aza/aza/pseudotripeptide without compromising the inhibition of hIAPP fibrillization.

#### Evaluation of the Selectivity Toward hIAPP Versus Aβ_1–42_ Fibrillization

In order to evaluate the relative selectivity toward hIAPP peptide, the ability of the most active inhibitors of hIAPP **1–3**, **8,** and **14** to interact with Aβ_1–42_, an amyloid protein involved in Alzheimer’s disease presenting about 50% of similarity of sequences but characterized by different amyloidogenic sequences, was also tested by the ThT-fluorescence assay (representative curves are shown in SupplementaryFigure 35). The longer peptide hairpins **1**–**3** were much less active on Aβ_1–42_ than on hIAPP fibrillization (Table 2), and much less active than our reported hairpins **G1b** and **G2b** based on specific SREs from Aβ_1–42_ (Pellegrino et al., 2017). No significant effect on the fibrillization kinetics of Aβ_1–42_ was observed for the shorter tripeptidic hairpin **8**. On the contrary to its activity on hIAPP, this hairpin displayed no effect on Aβ_1–42_ fluorescence plateau at 1/1 and 0.1/1 ratios, however, it showed a comparable effect at high concentration. The diaza-pseudopeptide analog **14** displayed no activity on the fluorescence plateau of Aβ_1–42_ even at 10/1 ratio while it was dramatically reduced in the case of IAPP (ΔF = −93%) but slightly delayed the kinetics of fibrillization of Aβ_1–42_ at 10/1 ratio (Δ *t*_1/2_ = +51% on Aβ_1–42_ vs. +186% on hIAPP). These results confirm that our design based on specific SREs of hIAPP is relevant, and also confirm that shortening the peptide or inserting diaza-pseudopeptide maintain the selectivity for hIAPP versus Aβ_1–42_.

**TABLE 2:** Effects of compounds **1**–**3**, **8,** and **14** on Aβ_1–42_ fibrillization assessed by ThT-fluorescence spectroscopy at 10/1, 1/1, and 0.1/1 ratios of compound/Aβ_1–42_.

Compound	Compound/hIAPP ratio^[a]^	*t*_1/2_ extension/reduction (%)^[b]^	F extension/reduction (%)^[c]^
**G1b**	(10/1)	NA^[d]^	−97 ± 1%
Pellegrino et al., 2017	(1/1)	NA	−90 ± 1%
**G2b**	(10/1)	NA	−95 ± 1%
Pellegrino et al., 2017	(1/1)	>+356 ± 12%	−73 ± 3%
**1**	(10/1)^[e]^	+99 ± 32%	−29 ± 3%
	(1/1)	+28 ± 4%	ne
	(0.1/1)	ne^[f]^	ne
**2**	(10/1)^[e]^	+102 ± 22%	+84 ± 23%
	(1/1)	ne	ne
	(0.1/1)	ne	−35 ± 6%
**3**	(10/1)^[e]^	+75 ± 21%	−14 ± 5%
	(1/1)	+43 ± 13%	−27 ± 8%
	(0.1/1)	ne	ne
**8**	(10/1)	−13 ± 4%	−61 ± 7%
	(1/1)	+13 ± 7%	ne
	(0.1/1)	ne	ne
**14**	(10/1)	+51 ± 19%	+8 ± 2%
	(1/1)	ne	ne
	(0.1/1)	ne	ne

*Parameters are expressed as mean ± SE, n = 3. ^[a]^Compounds were dissolved in DMSO; the final concentration kept constant at 0.5% (v). The concentration of Aβ_1–42_ was 10 μM. ^[b]^See Supplementary Material for the calculation of the t_1/2_ extension/reduction. ^[c]^See Supplementary Material for the calculation of the F extension/reduction. ^[d]^NA, no aggregation. ^[e]^Compounds **1, 2,** and **3** self-aggregate at 100 μM, ^[f]^ne, no effect.*

#### Morphology of hIAPP Fibrils by Transmission Electron Microscopy (TEM) Imaging

**FIGURE 5:**
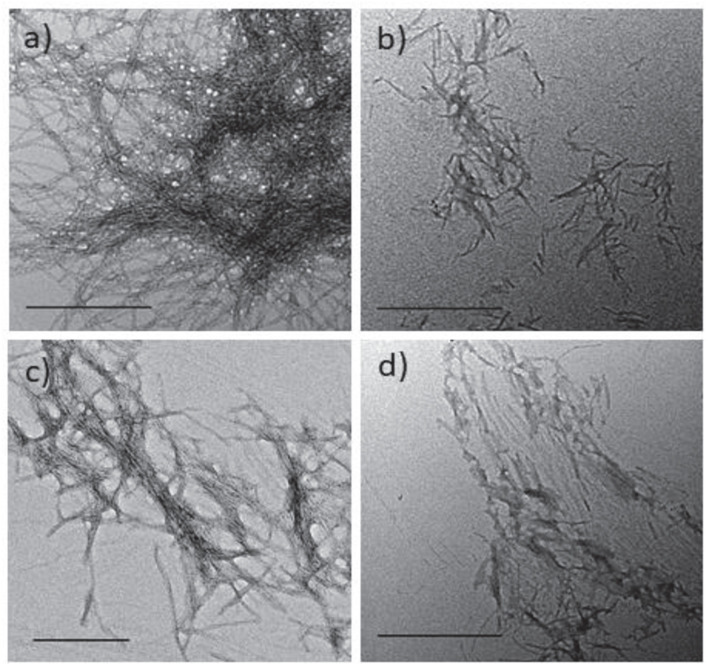
Effect of hairpins on the fibril formation of hIAPP visualized by TEM (scale bars 500 nm). Negatively stained images recorded after 17 h of incubation of a 5 μM solution (in 10 mM Tris–HCl, 100 mM NaCl, pH 7.4) of **(a)** hIAPP alone or in the presence of a 50 μM solution of hairpins: **(b) 3**, **(c) 8**, and **(d) 14**.

To evaluate the impact of the hairpins on the morphology of the hIAPP fibrillar material, transmission electron microscopy (TEM) images were acquired for hairpins **3**, **8,** and **14** which were the three most promising inhibitors of IAPP fibrillization in the ThT assays. These analyses were performed after 17 h of incubation, corresponding to the time needed to reach the complete conversion of hIAPP monomer into fibrils in the ThT assay. The hIAPP control sample showed the usual extremely dense network of long and tangled fibrils (Figure 5a). The compounds were tested at the hairpin/hIAPP ratio of 10/1. In the presence of hairpin **3** bearing pentapeptide sequences, a drastic reduction in the density of network formed by very shorter fibrils was observed compared to the control sample (Figures 5b vs. 5a). For both compounds **8** and **14** (Figures 5c,d) bearing two tripeptide sequences and one diaza-tripeptide sequence, respectively, the amount of fibers remained significantly less important and shorter than in the control grid but longer than for the sample **3**. The comparable density of the fibrils in samples **3**, **8,** and **14** was in accordance with the similar reduction of the fluorescence plateau in the ThT experiment. The fibers of the grid of **14** appeared less tangled compared to the other grids. Thus, these results were consistent with the ThT-fluorescence data and confirmed the efficiency of hairpins **3**, **8,** and **14** in disrupting and reducing the fibrillization process of hIAPP amyloid peptide.

#### Effect of the Hairpins on hIAPP-Membrane Fibril Formation and on Membrane Permeability

**FIGURE 6:**
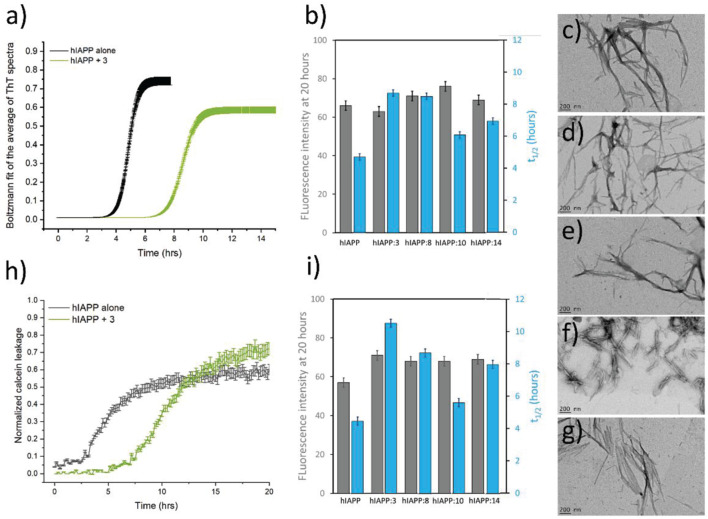
**(a)** Kinetics of hIAPP amyloid fibril formation in the absence (black) and in the presence (green) of the hairpin **3** (ratio 1/1) in 10 mM Tris–HCl, 100 mM NaCl buffer at pH 7.4. **(b)** The value of the intensity of the final plateau (gray) and the average half-time (t_1/2_, blue) are reported for each hairpin tested in the same condition (5 μM hIAPP, 5 μM hairpin, and 100 μM LUVs). Negatively stained microscopy images of mature hIAPP alone **(c)**, and in the presence of the hairpins **3 (d)**, **8 (e)**, **10 (f)**, and **14 (g)** at a ratio 1/1 after 24 h of incubation with vesicles composed of DOPC/DOPS (7/3) (scale bars 200 nm). **(h)** Kinetics of membrane disruption induced by 5 μM hIAPP in the absence (gray) and in the presence (green) of the hairpin **3** (ratio 1/1) in 10 mM Tris–HCl, 100 mM NaCl buffer at pH 7.4. hIAPP and the hairpin were added to the calcein-containing DOPC/DOPS LUVs (100 μM) at time zero. **(i)** The value of the intensity of the final plateau (gray) and the average half-time (t_1/2_, blue) are reported for each hairpin tested in the same condition (5 μM hIAPP, 5 μM hairpin, and 100 μM LUVs). The error bars shown were calculated as mean ± SE of three independent measurements in triplicate.

Several studies have demonstrated a clear interaction between hIAPP and lipid membrane and have shown that membrane can modify both the peptide structure and the kinetics of fibril formation (Caillon et al., 2014; Milardi et al., 2021). hIAPP is also known to modify the properties of membranes, by modulating the membrane structure and by altering the permeability. Therefore, the ability of the hairpins to reduce in the presence of membrane models (DOPC/DOPS LUVs) both hIAPP fibrillization and hIAPP-induced membrane damage was evaluated. Figure 6a (gray) showed typical S-shaped curves characteristic of hIAPP fibril formation in the presence of membranes. The presence of the hairpins **3** (Figure 6a) (green), **8**, **10,** and **14** at a 1/1 ratio, displayed the same S-shaped curve but we observed a significant decrease of the rate of hIAPP fibrillization from 4.7 h for hIAPP alone to 6.1–8.8 h with the compounds (Figure 6b and Supplementary Figure 35). This effect was even more pronounced with the compound **3**. However, the final plateau of fluorescence was not affected by the presence of hairpins. Afterward, we decided to study the morphology of hIAPP incubated with vesicles composed of DOPC/DOPS (7/3), in the presence of the hairpins using transmission electron microscopy. The hIAPP fibrils exhibited the typical morphology of long and twisted amyloid fibrils (Figure 6c). Similar fibrils were observed in the presence of hairpins **3**, **8**, **10,** and **14**, however, the network of the fibrils was less dense and had a lower frequency compared to the fibrils formed by hIAPP alone (Figures 6c–g). It is interesting to note that for the hairpins **3**, **8,** and **10**, a few vesicles were surrounded by the fibrils and that those vesicles in contact with the fibrils somehow showed distorted shapes.

The membrane leakage was then determined using the fluorescence signal of the encapsulated calcein, a highly fluorescent molecule whose fluorescence is self-quenched at high concentrations. High concentrations of calcein were thus encapsulated into LUVs, and upon membrane disruption by hIAPP, the dye was released, and the subsequent dilution leads to enhanced fluorescence. The fluorescence in the presence of the hairpins was compared to the one in the absence of the hairpins to validate their efficacy.

The leakage kinetics is shown in Figure 6 for hIAPP alone (gray) and in the presence of the hairpin **3** (green) in DOPC/DOPS vesicles at 25°C. The spontaneous calcein leakage through lipid membranes in the absence of hIAPP was found to be negligible. Our data indicate that 5 μM of hIAPP induced significant leakage in DOPC/DOPS vesicles to about 57% of the total vesicle content, in agreement with previous report (Hoffmann et al., 2018). The process of hIAPP-induced membrane leakage was characterized by an S-shaped curve with a t_1/2_ of approximately 4.5 h. The same experiments were done for hIAPP in the presence of the three most active hairpins **3**, **8,** and **14** and compared with an experiment in the presence of the slightly less active hairpin **10**. The compounds were evaluated at 1/1 ratio and similar sigmoidal curves were obtained but were more or less shifted. The kinetics data obtained from the sigmoidal curved are given in Figure 6h and indicate that the presence of **10** only slightly delay the kinetics of membrane damage from 4.5 h for hIAPP alone to 5.6 h while hairpins **3**, **8** and **14** have a more drastic effect in the t_1/2_ increase. Indeed, the t_1/2_ increases from 4.5 h for hIAPP alone to 10.5 h, 8.7 h, and 8.0 h for **3**, **8,** and **14,** respectively (Figure 6i).

Altogether, the ThT assays, TEM images and the membrane leakage assays indicated that the hairpins interacted strongly with hIAPP in the presence of membrane but are not able to totally inhibit hIAPP-fibril formation. Nevertheless, hairpins **3**, **8,** and **14** are able to decrease the fibrillization kinetics and thus delay hIAPP-induced membrane damage even at a low hairpin/hIAPP ratio of 1/1.

#### Effect on hIAPP Oligomerization Process by Capillary Electrophoresis (CE)

**FIGURE 7:**
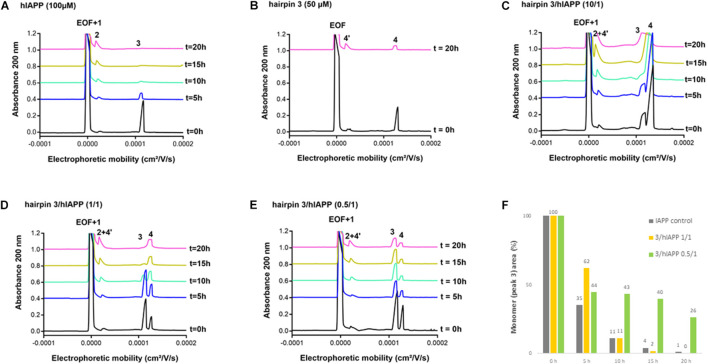
Electrophoretic profiles over time (0–20 h) of control samples of **(A)** hIAPP (100 μM) and **(B)** hairpin **3** (50 μM). EOF, electroosmotic flow; Peaks 1 and 2: large hIAPP oligomer species; Peak 3: monomer of hIAPP; Peak 4: hairpin **3**; Peak 4′: self-aggregated species of hairpin **3**. Electrophoretic profile over time (0–20 h) of hIAPP peptide (100 μM) incubated with **3** at **3**/hIAPP ratio of: **(C)** 10/1, **(D)** 1/1, **(E)** 0.5/1. **(F)** Evolution of the hIAPP monomer area (peak 3) over time in the absence or in the presence of **3** (at 1/1 and 0.5/1 ratios) relative to hIAPP monomer peak area at *t* = 0 h.

**FIGURE 8:**
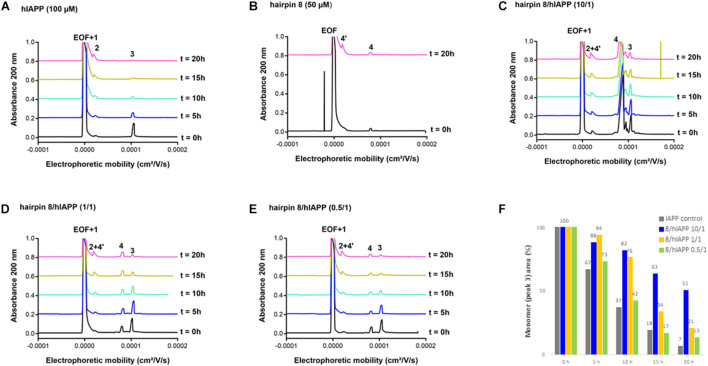
Electrophoretic profiles over time (0–20 h) of control samples of **(A)** hIAPP (100 μM) and **(B)** hairpin **8** (50 μM). EOF, electroosmotic flow peak; Peaks 1 and 2: large oligomer species; Peak 3: monomer of hIAPP; Peak 4: hairpin **8**; Peak 4′: self-aggregated species of hairpin **8**. Electrophoretic profile over time (0–20 h) of hIAPP peptide (100 μM) incubated with **8** at compound/hIAPP ratio of: **(C)** 10/1, **(D)** 1/1, **(E)** 0.5/1. **(F)** Evolution of hIAPP monomer area (peak 3) over time in the absence or in the presence of **8** (at 10/1, 1/1, and 0.5/1 ratios) relative to hIAPP monomer peak area at *t* = 0 h.

**FIGURE 9:**
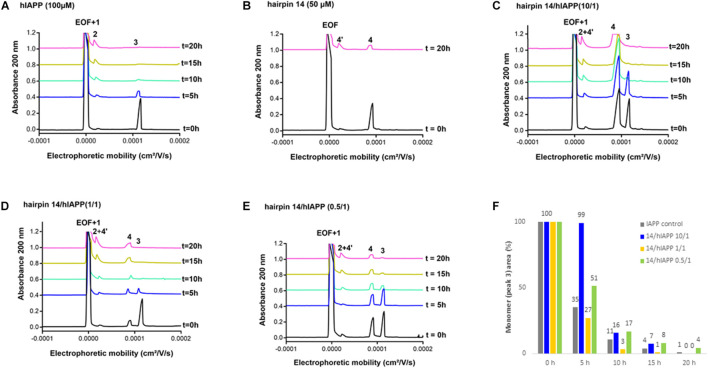
Electrophoretic profiles over time (0–20 h) of control samples of **(A)** hIAPP (100 μM) and **(B)** hairpin **14** (50 μM). EOF, electroosmotic flow; Peaks 1 and 2: large oligomer species; Peak 3: monomer of hIAPP; Peak 4: hairpin **14**; Peak 4′: self-aggregated species of hairpin **14**. Electrophoretic profile over time (0–20 h) of hIAPP peptide (100 μM) incubated with **14** at compound/hIAPP ratio of: **(C)** 10/1, **(D)** 1/1, **(E)** 0.5/1. **(F)** Evolution of the hIAPP monomer area (peak 3) over time in the absence or in the presence of **14** (at 10/1, 1/1, and 0.5/1 ratios) relative to hIAPP monomer peak area at *t* = 0 h.

We then evaluated the effect of the three hairpins **3**, **8,** and **14** on the oligomerization process of hIAPP peptide using our recently developed CE method able to monitor the soluble oligomeric species formed during the early hIAPP aggregation process (Berardet et al., 2018, 2020). The control electropherogram of hIAPP, shown in Figure 7A, displayed over time the typical profile, showing three groups of soluble species: peak 3 is mainly composed by monomers with possibly a small amount of dimers and trimers whereas peaks 1 and 2 are mainly assigned to oligomeric aggregates larger than 25-mers (peak 1 co-migrates with the electroosmotic flow EOF) (Berardet et al., 2018). Over time, the monomer peak 3 decreased steadily to almost disappear at 10 h and in the meantime, the oligomeric peak 2 regularly increased (Figure 7A). The CE evaluations were conducted at three different compound/hIAPP ratios: 10/1, 1/1, and 0.5/1. In the presence of hairpin **3** bearing two pentapeptide arms, at 1/1 ratio, the quantity of monomeric species (peak 3) was significantly preserved until 5 h while the two thirds of it disappeared in the control sample (62% of monomer maintained in the presence of **3** vs. 35% in the control) (Figures 7A,D,F). After 5 h, the kinetics of disappearance of the monomer was almost similar to the one observed for the control sample. Hairpin **3** was also tested at a 10-fold excess, but at this high concentration, the peak 4 corresponding to **3** itself (see control of **3**, Figure 7B) was very intense and partially (until *t* = 10 h) to totally (from *t* = 15 h) merged with the monomer peak 3, preventing its quantification (Figure 7C). This electrophoretic profile could suggest that one proportion of hairpin **3** self-assembles (peak 4) and another one binds to the monomeric species of hIAPP (peak 3); both association phenomena being in equilibrium and in competition. Surprisingly, in contrast with the ThT results, the hairpin **3**, displayed the stronger activity on hIAPP oligomerization at the substoichiometric ratio of 0.5/1 (**3**/hIAPP, Figures 7E,F); the monomer being maintained up to 26% until 20 h while it almost disappeared in the control hIAPP experiment at 10 h (Figure 7A). Concerning the control electrophoretic profile of hairpin **3** at 50 μM, a decrease and an enlargement of its peak 4 was observed during the kinetics while an additional peak 4′ migrating close to the EOF was observed and increased over the time (Figure 7B). This peak was attributed to self-aggregated species that might explain the lack of activity of hairpin **3** at higher ratio (we also suspected self-aggregation of hairpin **3**, as peak 4 was more intense at t0 in the experiment at 0.5/1 ratio than at the 1/1 ratio). In the case of the shorter peptidic hairpin **8**, the quantity of monomers was significantly maintained at both 10/1 and 1/1 ratios in a dose dependant manner. At a 10-fold excess, hairpin **8** (control of 8, Figure 8B) strongly maintained the monomer species during the whole kinetics; half remaining after 20 h (Figures 8C,F), while it almost totally disappeared in the control electropherogram (51 vs. 7% for respectively, **8**/hIAPP at 10/1 and hIAPP control, Figure 8A). At 1/1 ratio, the activity was still significant; 21% of the monomer was still visible at 20 h (Figures 8D,F), while at the substoichiometric ratio of 0.5/1 and unlike hairpin **3**, hairpin **8** was found inactive (Figures 8E,F). Like hairpin **8**, the azapeptidomimetic analog **14** displayed a strong activity at 10-fold excess (Figures 9C,F), but only at the beginning of the kinetics: the monomeric species being fully maintained while two thirds were no longer present in the control electropherogram at 5 h (99 vs. 35% for respectively, **14**/hIAPP at 10/1 and hIAPP control, Figure 9A). The hairpin **14** displayed no activity at 1/1 ratio (Figure 9D) and showed a very small activity at the substoichiometric ratio of 0.5/1 (**14**/hIAPP) (Figures 9E,F) until 5 h (51 vs. 35% for respectively, **14**/hIAPP at 0.5/1 and hIAPP control). Like hairpin **3**, **14** was suspected to self-aggregate due to the appearance of peak 4′ and the decrease of peak 4 in the control experiment (Figure 9B). In conclusion, hairpins **3**, **8,** and **14** are able to delay the early hIAPP oligomerization by preserving the monomers, described as non-toxic species (Abedini et al., 2016; Akter et al., 2016). However, the shorter hairpin **8** was very active at 1/1 and 10/1 ratio while the longer hairpin **3** was efficient at the lowest 0.5/1 ratio. In conclusion, these results show that the ability of these three hairpins to inhibit the oligomerization process is stronger when their tendency to self-aggregate is lower.

#### Effect on hIAPP Oligomerization Process by Native Mass Spectrometry Experiments

Over the last years, mass spectrometry (MS) and ion mobility coupling (IMS-MS) have been reported as a pertinent tool for the identification of the oligomeric species formed during the aggregation process. This was exemplified by the detection of prion oligomers using SEC and ion mobility coupled with mass spectrometry under “native” detection for the first time (Van der Rest and Halgand, 2017). A similar approach was used to characterize beta-amyloid oligomer-aggregates properties (Iuraşcu et al., 2009). In addition, they also have been reported as powerful methods for studying the mechanism of hIAPP aggregation inhibitors (Young et al., 2014, 2015, 2016; Li et al., 2015a; Riba et al., 2015; Berardet et al., 2020). Our reported method, using the same buffer as for CE experiments (Berardet et al., 2020; Kaffy et al., 2020), allowed us to study the effect of the hairpins **3**, **8,** and **14** on the oligomerization process of hIAPP at three different hairpin/hIAPP ratios (10/1, 5/1, and 1/1).

**FIGURE 10:**
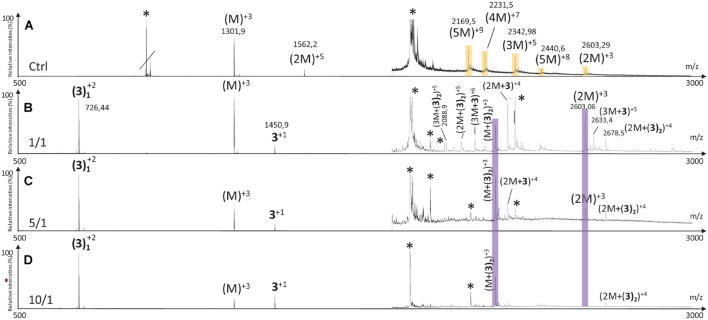
Mass spectra for the 500–3000 *m/z* range of **(A)** hIAPP control (hIAPP oligomers are labeled in orange) and hIAPP in the presence of hairpin **3** at three different ratios of **3**/hIAPP: **(B)** 1/1, **(C)** 5/1, and **(D)** 10/1. The different species are labeled with the number of hIAPP monomer, the addition of n molecules of hairpin **3** and the charge state of the ion corresponding to this complex {e.g., [2M + (3)_2_]^+5^, is the dimer complexed with two molecules of hairpin **3** and refers to the ion of *m/z* 2142.9 having a + 5 charge state}. Peaks labeled with an asterisk belong to contaminants. Regions labeled in purple illustrate relevant changes.

**FIGURE 11:**
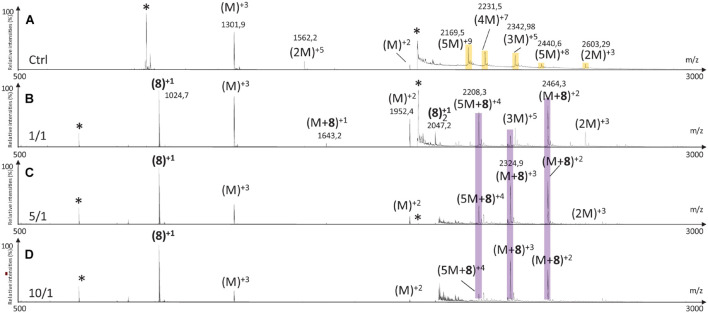
Mass spectra for the 500–3000 *m/z* range of **(A)** hIAPP control (hIAPP oligomers are labeled in orange) and hIAPP in the presence of hairpin **8** at three different ratios of **8**/hIAPP: **(B)** 1/1, **(C)** 5/1, and **(D)** 10/1. The different species are labeled with the number of hIAPP monomer, the addition of n molecules of hairpin **8** and the charge state of the ion corresponding to this complex [e.g., (5M + 8)^+4^]. Peaks labeled with an asterisk belong to contaminants. Regions labeled in purple illustrate relevant changes.

**FIGURE 12:**
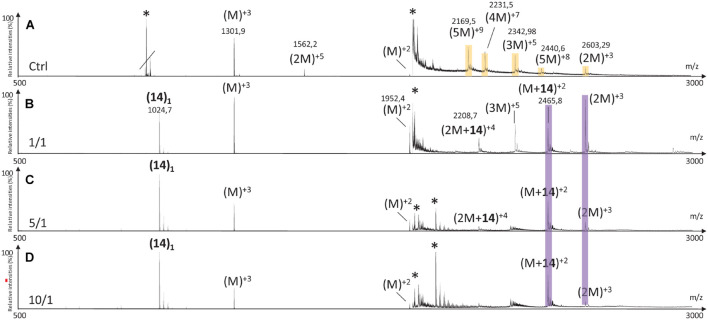
Mass spectra for the 500–3000 *m/z* range of **(A)** hIAPP control (IAPP oligomers are labeled in orange) and hIAPP in the presence of hairpin **14** at three different ratios of **14**/hIAPP: **(B)** 1/1, **(C)** 5/1 and **(D)** 10/1. The different species are labeled with the number of hIAPP monomer, the addition of n molecules of hairpin **14** and the charge state of the ion corresponding to this complex {e.g., [2M + (**14**)_2_]^+4^}. Peaks labeled with an asterisk belong to contaminants. Regions labeled in purple illustrate relevant changes.

The MS spectrum of hIAPP control presented the classical sets of multicharged monomeric and oligomeric species; dimers to pentamers were visible on the mass spectrum (Figures 10A, 11A, 12A). In the presence of hairpin **3** at the three different ratios, high mass oligomer species such as tetramers and pentamers were not any more visible. Dimers and trimers have decreased to finally almost completely disappear at ratio 10/1 (Figure 10D). At ratio 5/1 (Figure 10C) the formation of dimers and trimers was also strongly inhibited with only traces of dimers remaining. Finally, at the ratio 1/1 (Figure 10B), dimers and trimers were the only observed oligomers. Thus hairpin **3** has an anti-oligomerization activity on hIAPP preventing the formation of small oligomeric species, dimers up to pentamers. Noticeably, non-covalent complexes of **3** with hIAPP monomers and dimers [(M + **3**) and (2M + **3**)] were visible at the three ratios and stable under the ionization conditions, indicating that **3** preferentially interacts with the monomeric and the dimeric species of hIAPP. However, we cannot completely exclude that monomers and dimers could also result from minor higher order oligomers dissociation.

Addition of tripeptidic hairpin **8** at 10/1 ratio led to the inhibition of all oligomeric species (Figure 11D). Furthermore, the monomeric hIAPP complexed with **8** was the main visible species [(M + **8**)^+3^, (M + **8**)^+2^] with a very small quantity of a complex with the pentamer [(5M + **8**)^+4^]. The inhibition was also efficient at ratio 5/1 with the disappearance of trimers to pentamers and the presence of very small quantity of hIAPP dimer (2M)^+3^. Similarly, monomers in complex with **8** remained the main visible species (Figure 11C). At 1/1 ratio, trimer of hIAPP (3M)^+5^ were still present and a higher proportion of complexes of **8** with the pentamer were noticed (Figure 11B). Thus hairpin **8** has an efficient anti-oligomerization activity on hIAPP preventing the formation of oligomeric species. Moreover, it interacts preferentially with monomers and pentamers of hIAPP, by forming non-covalent complexes [(M + **8**) and (5M + **8**)]; the pentameric complex being mainly visible at the lowest 1/1 ratio and decreasing when the concentration of **8** increased.

Like in the case of hairpins **3** and **8**, the mass spectra in the presence of **14** (Figure 12) showed the disappearance of the higher mass oligomer species observed on hIAPP control. The effect on the formation of small oligomers (dimer and trimer) was dose dependent: almost none formed at the ratio 10/1 (Figure 12D), a very small amount of dimers at the ratio 5/1 (Figure 12C) and the presence of dimers and trimers for the ratio 1/1 (Figure 12B). Non-covalent complexes formed between hairpin **14** and monomers (M + **14**)^+2^ and dimers (2M + **14**)^+4^ of hIAPP at 1/1 and 5/1 ratios were also detected; the only complex detected at 10 fold excess being with the monomeric form of hIAPP (M + **14**)^+2^.

**FIGURE 13:**
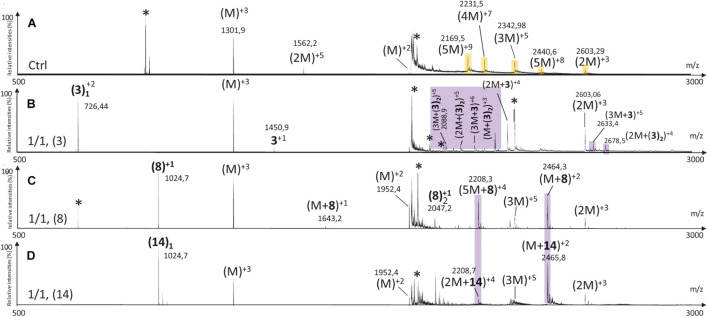
Mass spectra for the 500–3000 *m/z* range of hIAPP control **(A)** (IAPP oligomers are labeled in orange) and hIAPP in the presence of hairpin **3 (B)**, **8 (C)**, and **14 (D)** for 1/1 ratio. The different species are labeled with the number of IAPP monomer, the addition of n molecules of dedicated hairpin and the charge state of the ion corresponding to this complex {e.g., [2M + (hairpin)_2_]^+4^}. Peaks labeled with an asterisk belong to contaminants. Highlighted regions show regions of interest. Regions labeled in purple illustrate relevant changes.

By comparing the effect of the three hairpins at the lower ratio of 1/1, we observed that, among the oligomeric species of hIAPP formed in the control experiment, the pentamers and tetramers have disappeared in presence of the three hairpins at 1/1 ratio (Figure 13). The trimer (3M^+5^) was not detectable when incubated with the longer hairpin **3** but still visible in the presence of **8** and **14**. The dimer (2M^+3^) remained visible in the presence of the three hairpins. The main difference is that compound **3** formed non-covalent complexes mainly with dimers and trimers [(2M + **3**), (3M + **3**)] and in a smaller amount with the monomer of hIAPP [M + (**3**)_2_], whereas compounds **8** and **14** formed the same main non-covalent complex with monomeric hIAPP [(M + **8**/**14**)], and in a smaller amount one complex with pentamers and dimers for **8** and **14,** respectively [(5M + **8**) and (2M + **14**)]. These observations allowed us to conclude that hairpins **3**, **8,** and **14** exhibit an efficient anti-oligomeric activity and that the shorter hairpins **8** and **14** bind more preferentially to the monomeric species of hIAPP than the longer hairpin **3**.

## Discussion

Nowadays, the reduction of blood glycemic level is still the standard therapy in T2D, although it does not represent an etiological treatment. Over the past decades, increasing evidences on the link between hIAPP peptide deposits and loss of pancreatic β-cells has made the hIAPP peptide a promising target in the development of new treatments for T2D. Only few inhibitors of hIAPP aggregation and mainly natural compounds such as polyphenols (Pithadia et al., 2016), have been reported to date. Furthermore, they are generally described as non-selective because they have been reported as able to target other amyloid proteins such as Aβ_1–42_ involved in AD, but also to exert various other biological activities.

The recent dramatic increased of peptides approved or in active development in various therapeutic areas corroborates that peptides are reasonable alternatives to small molecule pharmaceuticals and to biologics (Henninot et al., 2018; Lau and Dunn, 2018). Greater efficacy, selectivity, and a reduced risk of unforeseen side-reactions compared to small organic molecules, combined with strategies to improve their pharmacokinetics properties explain this revival of interest of peptides in pharmaceutical science. Only few peptide or pseudopeptide derivatives (Gilead and Gazit, 2004; Cheng et al., 2012; Yan et al., 2013; Paul et al., 2017; Armiento et al., 2020) have been reported as hIAPP aggregation inhibitors.

We previously reported that designing specific acyclic β-hairpin peptidomimetics built on a piperidine-pyrrolidine β-turn inducer and on self-recognition elements (SRE), selected from Aβ_1–42_, were able to decrease its aggregation and to preserve Aβ_1–42_ monomers (Pellegrino et al., 2017; Tonali et al., 2018). These compounds were selective for Aβ_1–42_ and not active on hIAPP aggregation. In the present work we hypothesized that this strategy could be extended and applied to the selective inhibition of the aggregation of hIAPP.

By carefully selecting SREs derived from hIAPP amyloidogenic sequence and by modifying the length of the peptide sequences, the *N*-terminus or by incorporating a diaza-peptide motif, this present study allowed us to explore the inhibitory behavior in relation with the structural modifications introduced in our compounds.

The selection of the most active compounds was performed by ThT fluorescence spectroscopy, that is a technique exploring the kinetics of aggregation but that is more particularly informative on the late fibrillization process. Hairpins **3** and **4** based on models reported by Wiltzius et al. (2008), Liang et al. (2013) and Wu and Shea (2013) are bearing two SREs: the amyloidogenic sequence F_23_GAIL_27_ at their *C*-terminus and F_15_LVHSS_19_ at their *N*-terminus. They were more active than compounds **1** and **2** which were designed based on models reported by Kajava et al. (2005), Andreetto et al. (2015), and Mirecka et al. (2016), where the amyloidogenic sequence N_22_FGAIL_27_ faces A_13_NFLV_17_. This effect was even more pronounced at lower ratios 1/1 and 0.1/1. As **3** was slightly more active than **4**, we suggest that a free terminal amine is beneficial for both increasing the solubility and the activity.

By shortening the peptide sequence, we observed that three amino acids per arm in compounds **7**–**10** were sufficient to maintain the activity against hIAPP aggregation, although this activity was slightly lower than that of compound **3** bearing pentapeptides. Also in this series, the free terminal amine in **8** was more beneficial for the activity than its protection by respectively, Boc, acetyl and trifluoroacetyl in **7**, **9,** and **10** which tend to self-aggregate. Finally, the replacement of the *C*-terminal tripeptide sequence FLV of **8** by one α/aza/aza/pseudotripeptide (Phe-azaLys-azaVal-COCH_3_) led to compound **14** whose fibrillization inhibitory activity was even better than that of its peptide analog **8**. The evaluation of truncated molecule **11** and arms **5**, **6**, **12,** and **15**, corroborates the necessity of a whole hairpin to inhibit hIAPP aggregation. The ability of the best compounds **3**, **8,** and **14** to disrupt and reduce the late fibrillization of hIAPP was confirmed by TEM imaging. The lack of activity of the best compounds **3**, **8,** and **14** on Aβ_1–42_, compared with their effectiveness on hIAPP fibrillization, and compared to our reported **G1b** and **G2b** hairpins, designed for Aβ_1–42_ (Pellegrino et al., 2017), was next proven. These results validate our selection of SREs from hIAPP sequences.

The ability of compounds **3**, **8,** and **14** to modulate hIAPP aggregation reflects a strong delay of membrane damage induced by the presence of hIAPP peptide, as evaluated by calcein leakage through lipid membranes mimicked by DOPC/DOPS vesicles. However, in the presence of membranes, these three compounds were not able to totally prevent the fibrils formation.

The next step was to evaluate the ability of our compounds to decrease the presence of toxic oligomers and to maintain the presence of non-toxic monomers, as it is now well established that oligomers of hIAPP are toxic species (Bram et al., 2014; Abedini et al., 2016; Akter et al., 2016; Kiriyama and Nochi, 2018; Rodriguez Camargo et al., 2018). The very early steps of the oligomerization process overtime and the analysis of the effect of our hairpins on these challenging first stages was thus performed by our reported CE and MS methods (Berardet et al., 2018, 2020). The most promising compounds **3**, **8,** and **14** as inhibitors of hIAPP fibrillization had also a strong anti-oligomeric activity, and were able to stabilize the non-toxic monomeric species. The longer hairpin **3** was efficient in maintaining the monomer species in the CE and MS assays at 1/1 ratio and even at a lower ratio of 0.5/1 in the CE assay. While the shorter compounds **8** and **14** were more active at 10/1 ratio. Interestingly, we observed by MS that the three compounds **3**, **8,** and **14** dramatically decreased the presence of oligomers (from dimers to pentamers), however, compound **3** formed non-covalent complexes mainly with hIAPP oligomers (dimers and trimers), whereas compounds **8** and **14** formed non-covalent complexes essentially with monomeric hIAPP and in a smaller amount with pentamers for **8** and dimers for **14**.

The NMR conformational studies performed for the most active compounds of the series **3**, **8,** and **14**, as well as for the two acetyl derivatives **4** and **9** allowed us to confirm their β-hairpin like structure. However, a similar dynamic equilibrium between two different β-hairpin architectures was observed and was explained through MD simulations. These studies suggest that the conformation of Cα-C-N-C2 torsion angle between Leu-3 and Pip-4 impacts the spatial proximity of the two peptide arms. The presence of the semi-rigid piperidine-pyrrolidine scaffold allows flexibility that might be important for the activity. Indeed, this has been already suggested in our previous observations that dynamic hairpins based on this piperidine-pyrrolidine scaffold (Pellegrino et al., 2017) were much more efficient to inhibit Aβ_1–42_ than very stable hairpins based on diketopiperazine scaffold (Vahdati et al., 2017).

## Conclusion

The work reported in this paper constitutes the proof of concept that our strategy using flexible β-hairpin mimics represents a versatile approach for the development of compounds able to inhibit the aggregation of different amyloidogenic peptides. Our compounds rationally based on a semi-rigid piperidine-pyrrolidine β-turn mimic, and on specific small peptide arms selected from hIAPP, validate that they can be selective drug candidates to inhibit hIAPP aggregation involved in T2D. We also demonstrated that our hairpins are able not only to delay hIAPP fibrillization process but also to delay the early oligomerization and the membrane damage induced by hIAPP. With a view to developing a new future treatment for T2D, we demonstrated that it is possible to prepare a more druggable compound with lower peptide character while maintaining the selective inhibitory activity toward hIAPP versus another amyloid protein. The good inhibitory activities observed even at the low 1/1 ratio, were similar to those reported for related longer peptide compounds (Gilead and Gazit, 2004; Cheng et al., 2012; Yan et al., 2013; Andreetto et al., 2015; Paul et al., 2017; Armiento et al., 2020). Furthermore, the deeper structural analysis that we performed allowed us to draw some guidelines to better characterize the dynamic equilibrium between conformers of the hairpin compounds. Flexible β-hairpin mimics might adapt to different conformations and to different monomer or oligomer species of amyloid proteins to prevent the presence of toxic oligomers and to impact the fibrils formation. These small acyclic β-hairpins also represent valuable bio-chemical tools to further elucidate the interaction of amyloid proteins with membranes and lipids (Scollo and La Rosa, 2020) and with other components of cells, and to better understand the mechanism of toxicity of these proteins on membranes and on cells. These new results also support the hypothesis that compounds possessing several kinetically and thermodynamically accessible local minima representing conformations might be more powerful inhibitors compared to rigid ones in modulating protein-protein interactions (Ko et al., 2013; Li et al., 2015b). So, we believe that the use of these flexible small β-hairpin like compounds could be extended to study and combat other diseases associated with protein-protein interactions involving β-sheet structures, which are far from being understood or even discovered, as in cancer, in neurodegenerative diseases or infections (Laxio et al., 2019).

## Data Availability Statement

The original contributions presented in the study are included in the article/Supplementary Material, further inquiries can be directed to the corresponding authors.

## Author Contributions

SO designed the compounds, supervised the studies, and wrote the manuscript in collaboration with all authors. JL, FB, and SP performed the chemistry and conformational studies under the supervision of NT, SP, and CH. TH-D performed the simulation part. JK performed the ThT-fluorescence and TEM experiments. CB and JK performed the CE assays under the supervision of MT. LK was responsible for ThT-fluorescence and TEM in the presence of membrane as well as membrane permeability experiments. FH and CB performed the MS experiments. J-LS corrected the bibliographic and experimental parts. All authors contributed to the manuscript writing and gave approval to the final version of the manuscript.

## Conflict of Interest

The authors declare that the research was conducted in the absence of any commercial or financial relationships that could be construed as a potential conflict of interest.

## Publisher’s Note

All claims expressed in this article are solely those of the authors and do not necessarily represent those of their affiliated organizations, or those of the publisher, the editors and the reviewers. Any product that may be evaluated in this article, or claim that may be made by its manufacturer, is not guaranteed or endorsed by the publisher.

## Footnotes

1
https://www.chemaxon.com/
