# Supplementary material for: β-Hairpin Peptide Mimics Decrease Human Islet Amyloid Polypeptide (hIAPP) Aggregation
Source: Front Cell Dev Biol. 2021 Sep 16;9:729001. doi: 10.3389/fcell.2021.729001 (PMC8481668; doi:10.3389/fcell.2021.729001)
Supplement: Supplementary file 1 [file Data_Sheet_1.pdf]

β-Hairpin peptide mimics decrease human Islet Amyloid Polypeptide (hIAPP) Aggregation

Jacopo Lesma,[1] Faustine Bizet,[1] Corentin Berardet,[1,2] Nicolo Tonali,[1] Sara Pellegrino,[3] Myriam Taverna,[2] Lucie Khemtemourian,[4] Jean-Louis Soulier,[1] Carine van Heijenoort,[5] Frédéric Halgand,[6] Tâp Ha-Duong,[1] Julia Kaffy,*[1] and Sandrine Ongeri *[1]

[1] BioCIS, Université Paris-Saclay, CNRS, 92290 Châtenay-Malabry, France.
[2] Université Paris Saclay, CNRS, Institut Galien de Paris Saclay, 92290 Châtenay-Malabry, France
[3] DISFARM-Sez. Chimica Generale e Organica "A. Marchesini", Università degli Studi di Milano, via Venezian 21, 20133 Milano, Italy
[4] Institute of Chemistry & Biology of Membranes & Nanoobjects (CBMN), CNRS UMR 5248, Université de Bordeaux, Institut Polytechnique Bordeaux, 33600 Pessac, France.
[5] Université Paris Saclay, CNRS, ICSN, Equipe Biologie et Chimie Structurales, Dept Chimie et Biologie Structurales et Analytiques, 91190 Gif sur Yvette France.
[6] Institut de Chimie Physique, Equipe Chimie Analytique Physicochimie Réactivité des Ions, Université Paris-Saclay, CNRS, Institut de Chimie Physique, 91405, Orsay, France.

Table of content

Page S2 General experimental methods for the preparation of the compounds

Page S3-S39 Synthesis, characterization and conformational analyses of protected and deprotected hairpins **1-4**, **7-11** and **13-14**.

Page S40-S49 Synthesis and characterization of the intermediates

Pages S49 Protocol for the preparation of large unilamellar vesicles

Pages S49-S58 Protocol for Fluorescence-Detected ThT Binding Assay of hIAPP in solution and in the presence of DOPC/DOPS LUVs and Representative curves of Tht fluorescence assays over time showing hIAPP aggregation in presence of compounds **1-15**

Pages S58-S61 Protocol for Fluorescence-Detected ThT Binding Assay of $A\beta_{1-42}$ and Representative curves of Tht fluorescence assays over time showing $A\beta_{1-42}$ aggregation in presence of compounds **1-3, 8, 14**

Pages S61 Representative curves of the kinetics of membrane disruption induced by 5 µM hIAPP in the absence and in the presence of the compounds **3**, **8**, **10** and **14**

Pages S62 Protocol for transmission electron microscopy

Page S62 Protocol for membrane permeability assay

General experimental methods for the preparation of the compounds

Synthesis, NMR, HPLC, Mass

Usual solvents were purchased from commercial sources, dried and distilled by standard procedures. Pure compounds were obtained after liquid chromatography using Merch silical gel 60 (40–63 µm). TLC analyses were performed on silica gel 60 F250 (0.26 mm thickness) plates. The plates were visualized with UV light (λ= 254 nm) or revealed with a 4 % solution of phosphomolybdic acid or ninhydrin in EtOH.

NMR spectra were recorded on a Bruker AMX 200 (^{1}H, 200MHz; 19F, 188 MHz), an ultrafield Bruker AVANCE 300 (^{1}H, 300 MHz, ^{13}C, 75 MHz), a Bruker AVANCE 400 (^{1}H, 400 MHz, ^{13}C, 100 MHz, ^{19}F, 376 MHz), a Bruker AVANCE I 600MHz (^{1}H, 600 MHz, ^{13}C, 150 MHz, ^{19}F, 564 MHz) equipped with a z-gradient TCI and 19F-QCI, or a Bruker AVANCE III 800MHz (^{1}H, 800 MHz, ^{13}C, 200 MHz) spectrometers, equipped with a z-gradient TCI cryprobe.

Chemical shifts (δ) are in ppm and downfield from Me_4Si (δ = 0.0 ppm) with the solvent resonance as the internal standard (^{1}H NMR, CDCl3: δ = 7.26 ppm, CD_3OD and CD_3OH: δ = 3.31 ppm; ^{13}C NMR, CDCl3: δ = 77.16 ppm, CD_3OD and CD_3OH: δ = 49.00 ppm). the following abbreviations are used: singlet (s), doublet (d), doublet of doublet (dd), triplet (t), quadruplet (q), multiplet (m) and broad singlet (bs).

Melting points were determined on a Kofler melting point apparatus. High-resolution mass spectra (HRMS) were obtained using a TOF LCT Premier apparatus (Waters), with an electrospray ionization source. The purity of compounds was determined by HPLC using a WATERS gradient system (pump + controller E 600, UV detector PDA 2996, autosampler 717) on a Xselect column (C18, 2.1 x150mm-3.5µm), mobile phase, $MeCN/H_2O$ + 0.1% formic acid (gradient 5-100% in 20 min), detection at 257 nm.

Preparative HPLC were performed on Agilent Infinity II.

The most active inhibitors of hIAPP aggregation in the ThT fluorescence assays **3-4**, **8-9** and **14** were subjected to conformational studies by NMR in CD_3OH. When signals were sufficiently resolved and disperse, vicinal $^3J_{HN\text{-}H\alpha}$ coupling constants, H^{α}-HN ROE correlations, temperature coefficient ($\Delta\delta_{HN}/\Delta T$) of the amide protons and $^1H_{\alpha}$ and $^{13}C_{\alpha}$ chemical shift deviations (CSD), were examined to analyze backbone conformational propensities.

Synthesis and characterization of the final hairpins:
Synthesis of compounds 1-4.

Compounds **1**, **2**, **3** and **4** were synthesized on Rink-amide resin (250 mg, 0.72 loading) using standard conditions (AA/HOBT/HBTU/DIPEA, 5:5:5:10, 1 h coupling and then 20% piperidine in DMF for Fmoc deprotection). (S. Pellegrino, C. Annoni, A. Contini, F. Clerici, M. L. Gelmi *Amino Acids* **2012**, *43*, 1995-2003).

The coupling of unnatural Fmoc-protected scaffold (1-[(3R,4R)-1-(((9H-Fluoren-9-yl)methoxy)carbonyl)-4-(4-methylphenylsulfonamido)piperidin-3-yl]-pyrrolidine-2-(S)-carboxylic acid, 1.5 eq) was performed on the peptide growing chain linked to rink-amide resin using HOBT and HBTU (1.5 eq) and DIPEA (3 eq) and standing the mixture under shaking overnight. (S. Pellegrino, N. Tonali, E. Erba, J. Kaffy, M. Taverna, A. Contini, M. Taylor,D. Allsop, M..L. Gelmi, S. Ongeri *Chem. Sci.* **2017**, *8*, 1295-1302).

For compound **4** the acetylation was performed on resin using Ac_2O (10 eq) and DIPEA (10 eq), performing two couplings for 1h each one.

The final cleavage was performed using trifluoroacetic acid (TFA, 8 mL), triisopropylsilane (TIS, 400 µl), thioanisole (400 µl), water (200 µl) and phenol (0.6 g) for 4 h. After the cleavage, the peptides were precipitated and washed using ice-cold anhydrous ethyl ether. The peptides were purified by RP-HPLC using a gradient elution of 95–30% solvent A (solvent A: water/acetonitrile/trifluoroacetic acid 95 : 5 : 0.1; solvent B: water/acetonitrile/trifluoroacetic acid 5 : 95 : 0.1) over 20 min at a flow rate of 20 mL/min^{-1}. The purified peptides were freeze-dried and stored at 0 °C. HPLC analysis were carried out using a gradient elution of 2–60% solvent B (solvent A: water + 0.1%TFA; solvent B: acetonitrile/+ 0.1% TFA) over 20 min at a flowrate of 0.8 ml/min.

For compounds **3** and **4** a single dispersion of the NH chemical shifts (Tables 1 and 2) was observed indicating the presence of one predominant conformation for both compounds. ROESY experiments confirmed the presence of a hairpin structure as already reported for similar compounds (Pellegrino et al., 2017; Pellegrino et al., 2014; Tonali et al., 2018), as more specifically reported below on acetylated compound **4** in CD_3OH.

Compound [3]

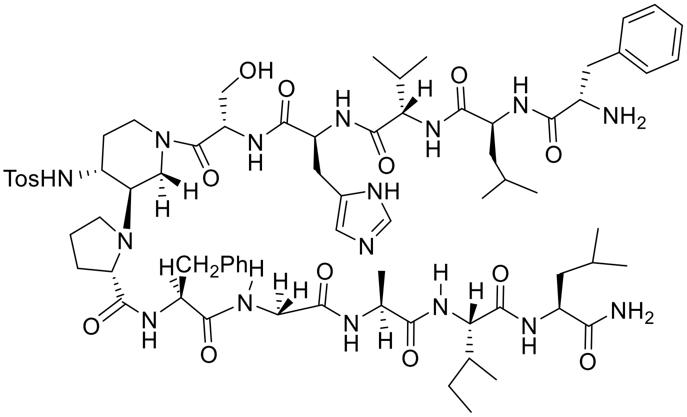

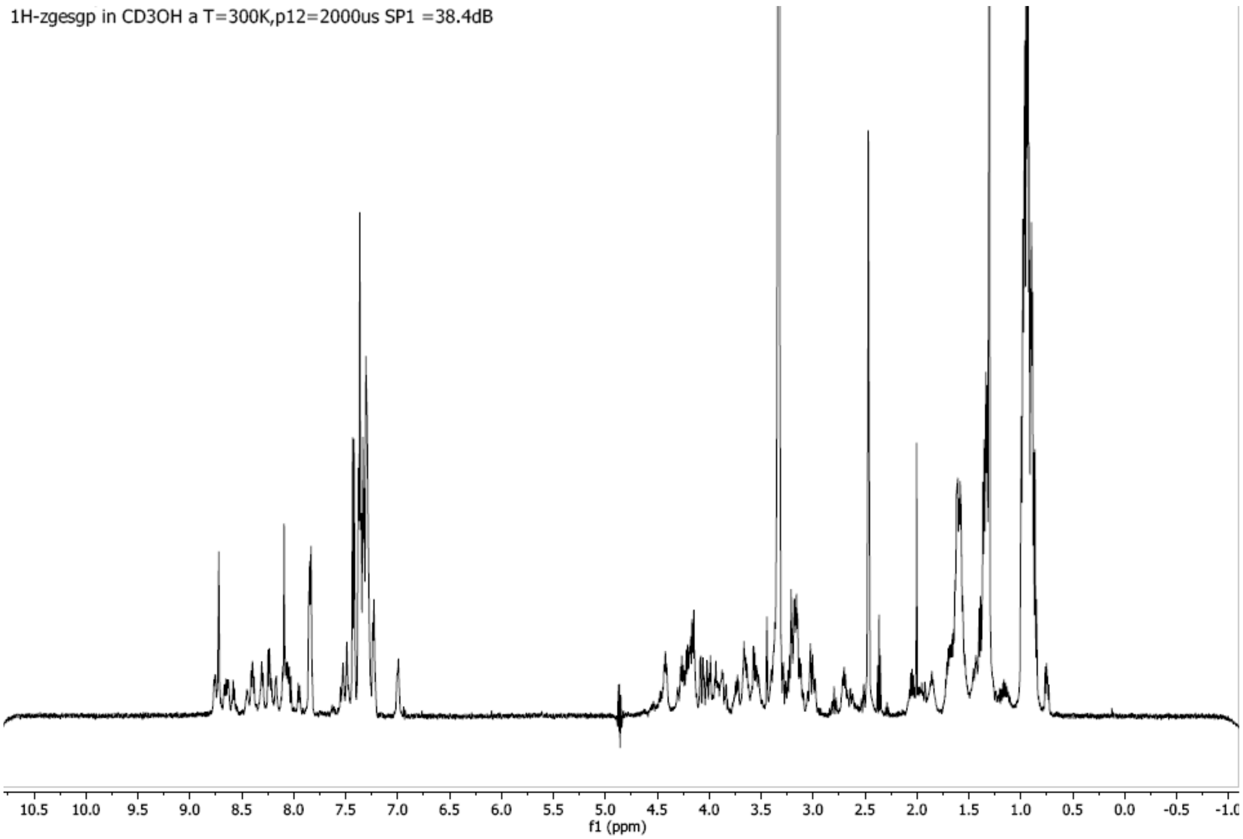

Figure S1. ^{1}H-NMR spectrum of compound **3** in CD_3OH at 300K (6.5 mM).

Residue	NH	α-H	β-H	Other	Roesy[a]
**Phe-1**	8.76	4.88 (overlapped)	3.38, 3.24 (overlapped)	Ar: 7.32-7.10 (overlapped)	
**Leu-2**	8.04	4.28	1.88 (overlapped)	0.97 (overlapped)	
**Val-3**	8.38	4.20	2.08	0.97 (overlapped)	
**His-4**	8.22	4.71 (overlapped)	3.17, (overlapped) 3.02	8.72, 7.31	NH: Ha Val
**Ser-5**	8.23	4.49 (overlapped)	4.17, 3.68	-	Hb:Hb Phe 7
**Scaffold**			H-2: 4.08, H-2': 3.60<br>H-3: 3.56; H-4: 2.65<br>H-5: 1.46, 1.24<br>H-6: 4.91, H-6': 3.26<br>NH: 7.53<br>Arom: 7.85*o*, 7.41*m*;<br>Me: 2.48		H2: Hb Phe7
**Pro-6**		4.77	H-5: 3.05 H-5': 2.70; H-4: 1.61, 1.46; H-3: 1.98		H5:H2' scaffold
**Phe-7**	8.65	4.75 (overlapped)	3.36, 3.22 (overlapped)	Ar: 7.32-7.10 (overlapped)	Hb: H4 scaffold
**Gly-8**	8.44	4.04, 3.87	-	-	
**Ala-9**	8.16	4.45	1.37	-	
**Ile-10**	8.23	4.16	2.08	1.37, 0.98 (overlapped)	
**Leu-11**	7.95	4.25	1.89 (overlapped)	0.97 (overlapped)	
**NH_2**	7.44, 6.99	-	-	-	

Table S1 ^{1}H-NMR chemical shifts for compound **3** (6.5 mM) in CD_3OH at 300K. [a]Only significant ROEs are reported.

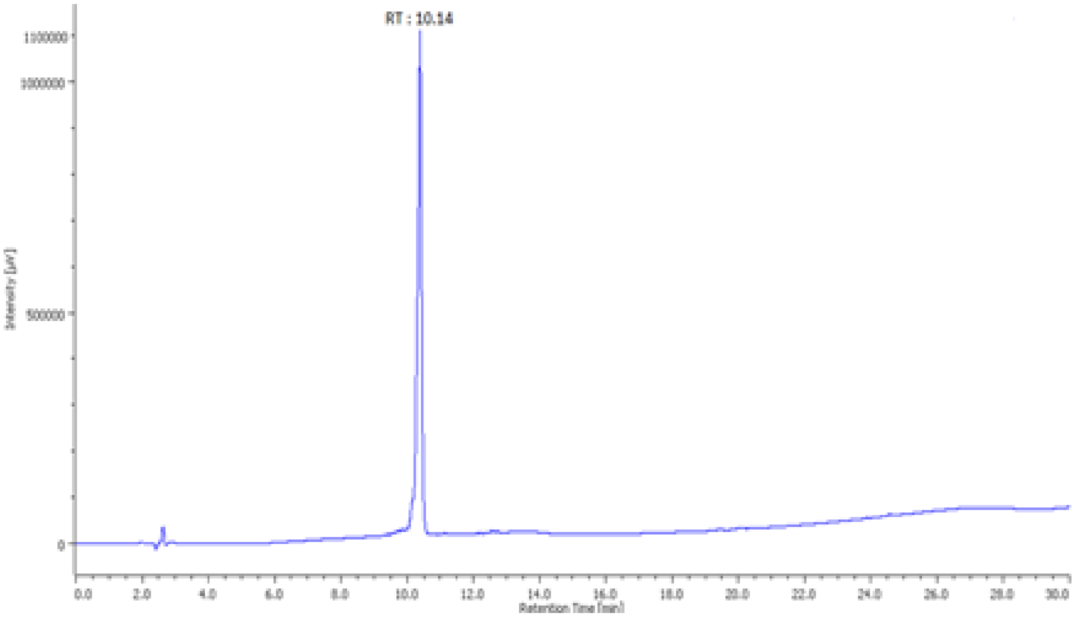

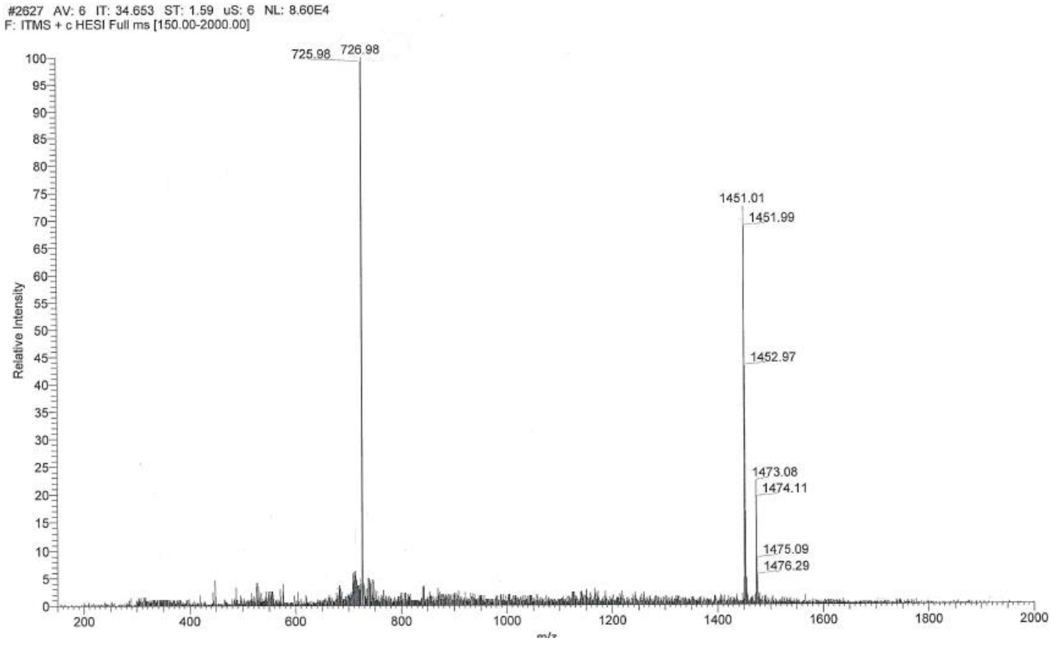

(MW+23) $_{calc}$ (Da) = 1473.12; (MW+1) $_{calc}$ (Da) = 1451.65; (MW+2)/2 $_{calc}$ (Da) = 725.53.

Figure S2 HPLC chromatogram and ESI-MS spectrum of compound **3**.

Compound [4]

^{1}H-NMR experiments were performed on acetylated compound **4** in CD_3OH (6.5 mM, Table S2). A good dispersion of the NH chemical shifts was observed indicating the presence of a predominant conformation of the peptide. On the other hand, several overlapping among the Ha and Hb signals was detected. ROESY experiments suggested the presence of a turn structure around the piperidine-pyrrolidine scaffold as already reported for similar compounds (Pellegrino, S., *Chem. Sci.* **2017**, *8*, 1295-1302; Pellegrino, S. *RSC Adv.* **2016**, *6*, 71529 – 71533; Pellegrino, S. *J. Org. Chem.* **2014**, *79,* 3094-3102). In particular, a significative Roe proximity was present between H2 scaffold and Hβ Phe7. Regarding the FGAIL arm, a series of sequential NH- Hα Overhauser effects was observed, suggesting an extended configuration of this part of the molecule. On the other hand, in the FLVHS sequence, the NH- Hα Roe effect was only present between His and Val, hampering the definition of a specific conformation of the peptide fragment. Unfortunately, due to the several overlapping of the signals it is not possible to unequivocally assign interstrand spatial proximities. The variable temperature experiments showed that NH Gly8 is characterized by a Δδ/ΔT of around 3.5 ppb/K, indicating its involvement in medium strength hydrogen bonds that probably stabilize the turn structure around the non natural scaffold.

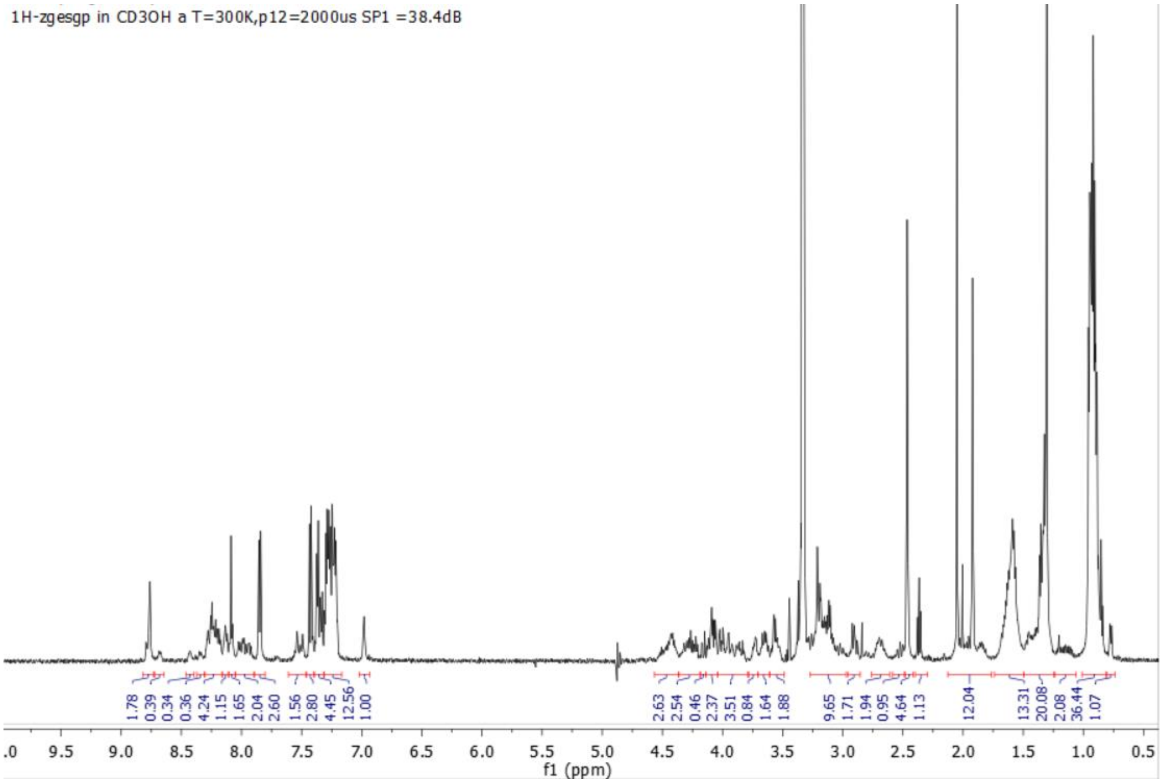

Figure S3. ^{1}H-NMR spectrum of compound **4** in CD_3OH at 300K (6.5 mM).

Residue	NH	α-H	β-H	Other	Roesy[a]
**-COCH₃**	-	2.08	-	-	
**Phe-1**	8.76	4.80 (overlapped)	3.40, 3.22 (overlapped)	Ar: 7.32-7.10 (overlapped)	
**Leu-2**	8.07	4.31	1.84 (overlapped)	0.97 (overlapped)	
**Val-3**	7.94	4.09	2.07 (overlapped)	0.97 (overlapped)	
**His-4**	8.33	4.89 (overlapped)	3.40, (overlapped) 3.02	8.75, 7.32	NH: Hα Val<br>Hβ: Hα Gly
**Ser-5**	7.99	4.85 (overlapped)	3.70, 3.65	-	
**Scaffold**	7.48		H-2: 4.08, H-2': 3.84<br>H-3: 3.57; H-4: 2.73<br>H-5: 2.00, 1.29<br>H-6: 4.54, H-6': 2.85<br>Arom: 7.85*o*, 7.42*m*;<br>Me: 2.47		H2': Hα Phe7<br>H2: Hβ Phe7
**Pro-6**		3.90	H-5: 3.02, H-5': 2.56; H-4: 1.58, 1.46; H-3: 2.06, 1.89		H5:H2' scaffold
**Phe-7**	8.66	4.80 (overlapped)	3.40, 3.22 (overlapped)	Ar: 7.32-7.10 (overlapped)	
**Gly-8**	8.44	4.04, 3.85	-	-	Hα: Hβ His
**Ala-9**	8.17	4.44	1.36	-	NH: Hα Gly
**Ile-10**	8.09	4.25	2.08 (overlapped)	1.90, 0.94 (overlapped)	NH: Hα Ala
**Leu-11**	8.12	4.41	1.84 (overlapped)	0.97 (overlapped)	NH: Hα Ile
**NH₂**	7.52, 6.97	-	-	-	

Table S2 ^{1}H-NMR chemical shifts for compound **4** (6.5 mM) in CD_3OH at 300K. [a]Only significant ROEs are reported.

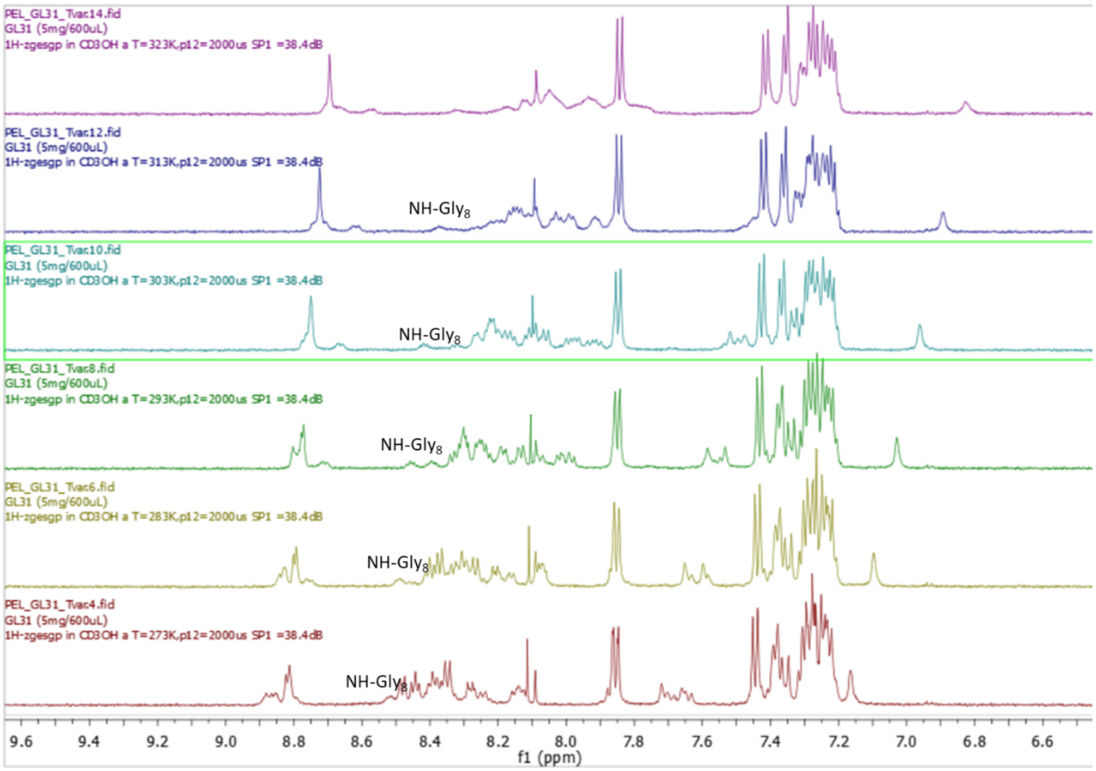

Figure S4 ^{1}H-NMR amide region spectra at different temperatures from 273K to 323K in CD_3OH for compound **4**

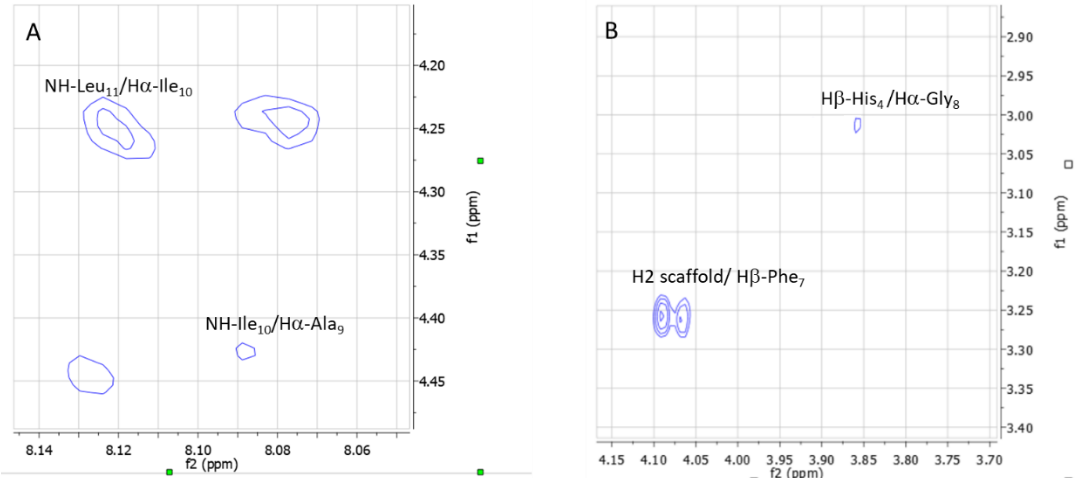

Figure S5 Significative Roe proximities observed for compound **4** (A: NH-Hα region; B: Hα-Hβ region)

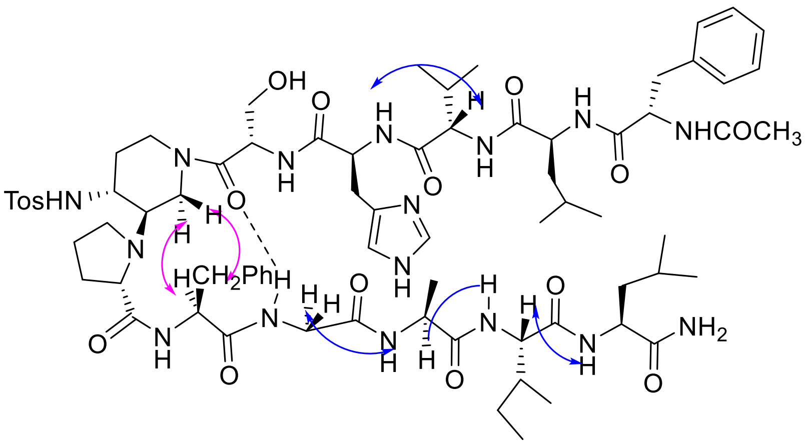

Figure S6 Structure of hairpin **4** showing the assigned ROEs: in blue the sequential $CH\alpha_i/NH_{i+1}$ ROEs, in pink other significant ROEs.

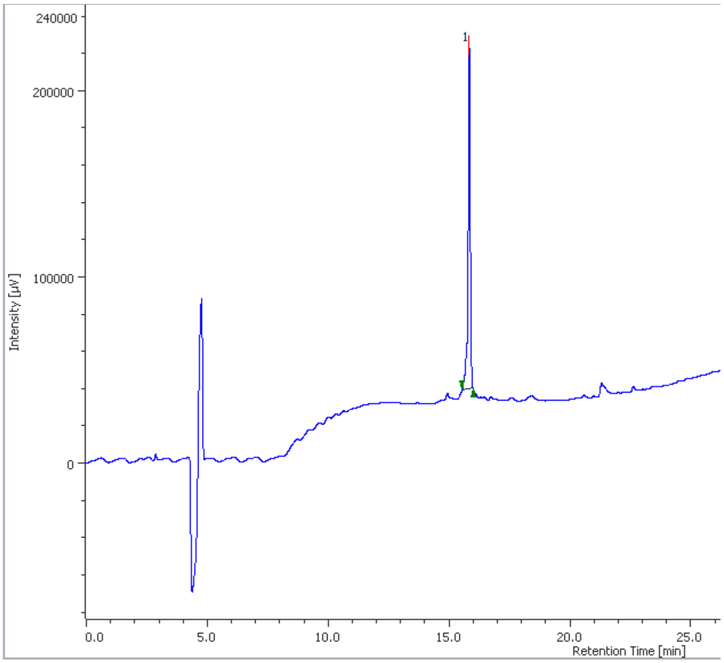

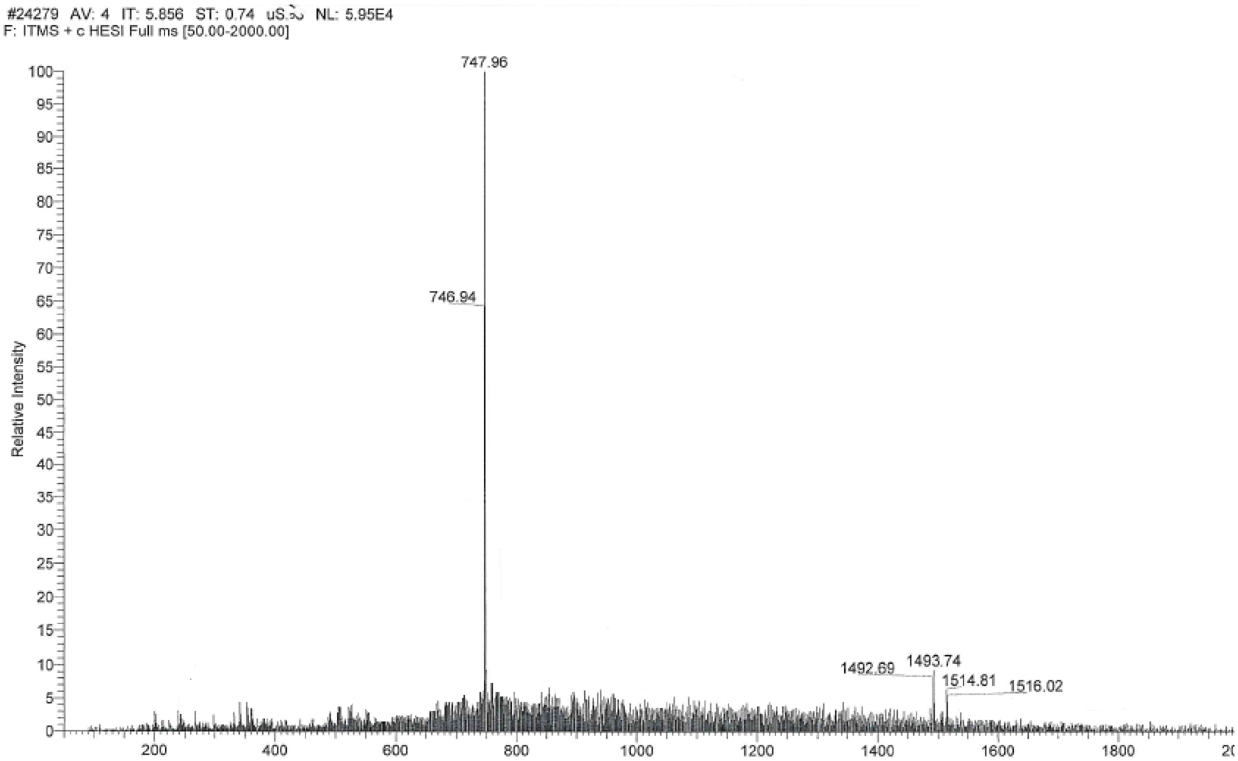

(MW+23) calc (Da) = 1515.88; (MW+1) calc (Da) = 1493.70; (MW+2)/2 calc (Da) = 746.78.

Figure S7 HPLC chromatogram and ESI-MS spectrum of compound **4**.

Compound [7]

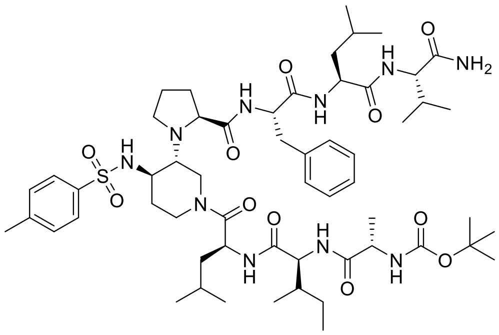

To a stirred solution of **19** (232 mg, 0.303 mmol, 1.0 eq.) in dry DMF (5 mL), cooled at 0°C, COMU (130 mg, 0.303 mmol, 1.0 eq) and oxyma (43 mg, 0.303 mmol, 1.0 eq.) were added. The reaction mixture was stirred for 30 min at 0°C, at that moment **12** (125 mg, 0.303 mmol, 1.0 eq.) and DIPEA (82 µL, 0.606 mmol, 2.0 eq.) were added. The solution was stirred at room temperature overnight under argon atmosphere and the volatile was removed under reduced pressure. The crude oil obtained was taken up with EtOAc, washed with saturated solution of $NaHCO_3$, water and

brine, dried over Na_2SO_4, filtered and concentrated under reduced pressure. The residue obtained was purified by column chromatography on silica gel, eluting with CH_2Cl_2/MeOH 95:5 to afford **7** (297 mg, 0.264 mmol, 87%) as a white solid.

Molecular weight = 1122.60 g mol^{-1}
Rf = 0.4 (CH_2Cl_2/MeOH 95:5)
HRMS: Calcd. for $[C_{57}H_{90}N_{10}O_{11}S + H]^+$: m/z 1123.6590, found: 1123.6576 g mol^{-1}
^{1}H NMR (CD_3OH, 600 MHz): two conformers δ 8.74 (0.5H, m); 8.69 (0.5H, d, *J* = 10 Hz); 8.60 (0.5H, d, *J* = 9.5 Hz); 8.46 (0.5H, m); 8.39 (0.5H, d, *J* = 7.6 Hz); 8.35 (0.5H, d, *J* = 8.2 Hz); 8.27 (0.5H, d, *J* = 8.5 Hz); 8.14 (0.5H, d, *J* = 9.0 Hz); 7.96 (0.5H, d, *J* = 9.2 Hz); 7.95-7.87 (1H, m); 7.83 (2H, m); 7.77 (1H, d, *J* = 7.9 Hz); 7.72 (0.5H, d, *J* = 9.8 Hz); 7.41 (2H, d, *J* = 8.9 Hz); 7.36 (2H, m); 7.26 (2H, m); 7.23-7.17 (1.5H, m); 7.15 (0.5H, bs); 6.97 (0.5H, d, *J* = 7.3 Hz); 6.91 (0.5H, d, *J* = 7.5 Hz); 4.86 (1H, bs); 4.78 (1H, bs); 4.57 (1H, bs); 4.44 (1H, d, *J* = 14.1 Hz); 4.28 (1H, d, *J* = 14.6 Hz); 4.25-4.00 (3.5H, m); 3.83-370 (1H, m); 3.68-3.50 (1H, m); 3.39-2.98 (4.5H, m); 2.80-2.66 (1H, m); 2.61 (0.5H, m); 2.52-2.41 (1H, m); 2.44 (3H, s); 2.22 (0.5H, m); 2.05 (1H, m); 1.93 (1H, m); 1.80-1.04 (16H, m); 1.43 (9H, s); 1.01-0.80 (21H, m); 0.74 (3H, d, *J* = 7.0 Hz) ppm
^{13}C NMR (CD_3OH, 150 MHz, 278K): two conformers δ 176.0; 174.7; 174.5; 173.4; 173.3; 172.0; 171.5; 171.3; 143.5; 139.6; 137.5; 129.7; 129.2; 128.1; 126.7; 126.4; 79.2; 63.4; 62.8; 60.3; 59.5; 58.8; 58.5; 57.6; 57.2; 54.3; 54.1; 53.8; 51.8; 50.3; 47.4; 46.7; 44.5; 44.2; 43.8; 41.0; 40.8; 40.7; 40.6; 40.4; 40.3; 39.8; 37.4; 36.9; 31.6; 30.9; 30.8; 27.4; 24.6; 24.5; 24.1; 21.0; 20.3; 18.0; 17.7; 17.0; 14.9; 10.1; ppm
IR: 3300; 2963; 1643; 1523; 1453 cm^{-1}
HPLC purity: XBridge C18 3.5 μm; H2O + 0:2 % form. ac./ACN, gradient 5–100 % in 20 min; R_t = 18.68 min, 100 %

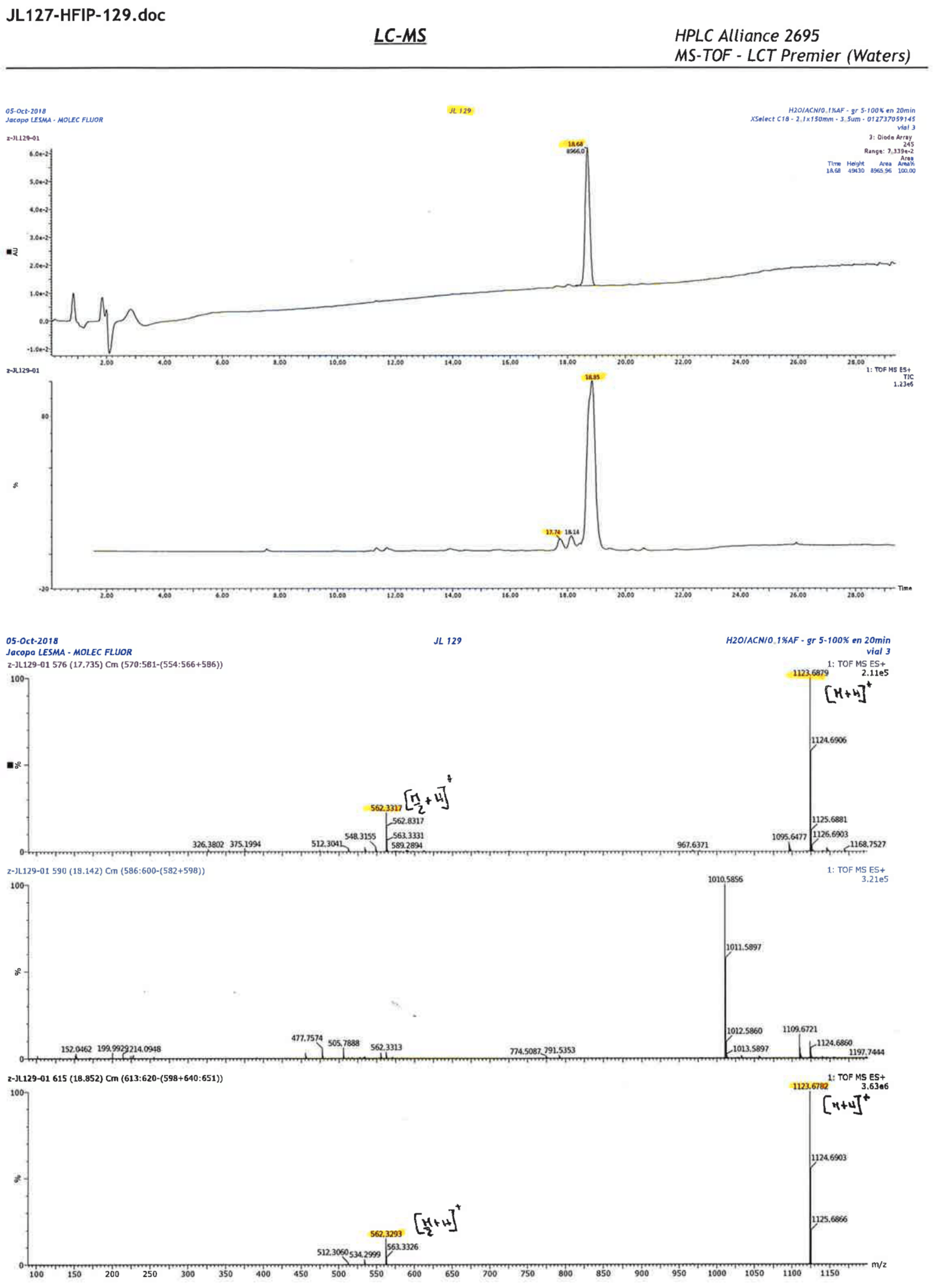

Figure S8 HPLC chromatogram and ESI-MS spectrum of compound **7**.

Compound [8]

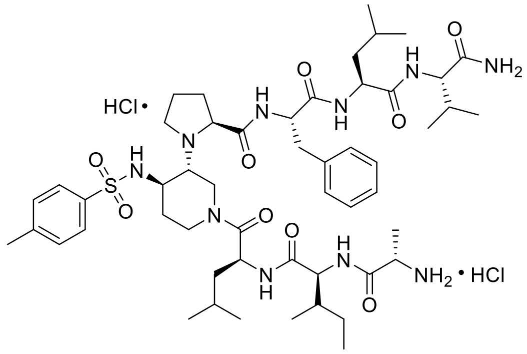

To a solution **7** (260 mg, 0.231 mmol, 1.0 eq.) in CH_2Cl_2 (15 mL) under argon atmosphere, HCl 4 M in dioxane (1.25 mL, 5 mmol, 20.0 eq.) was added at 0°C. A white precipitate immediately appeared. The mixture was stirred for 4 h at room temperature, decanted and the solvent was carefully removed with a Pasteur pipette. The solid obtained was washed twice with Et_2O, dried under reduced pressure to afford the hydrochloride salt **8** (247 mg, 0.225 mmol, 98%) as a white powder.

Molecular weight = 1096.27 g mol^{-1}; free amine 1022.60 g mol^{-1}
Rf = 0 (CH_2Cl_2/MeOH 95:5)
HRMS: Calcd. for $[C_{52}H_{82}N_{10}O_9S + H]^+$: m/z 1023.6065, found: 1023.6061 g mol^{-1}
IR: 3251; 2963; 1647; 1546; 1456 cm^{-1}
HPLC purity: XBridge C18 3.5 µm; H2O + 0:2 % form. ac./ACN, gradient 5–100 % in 20 min; R_t = 13.16 min, 92 %

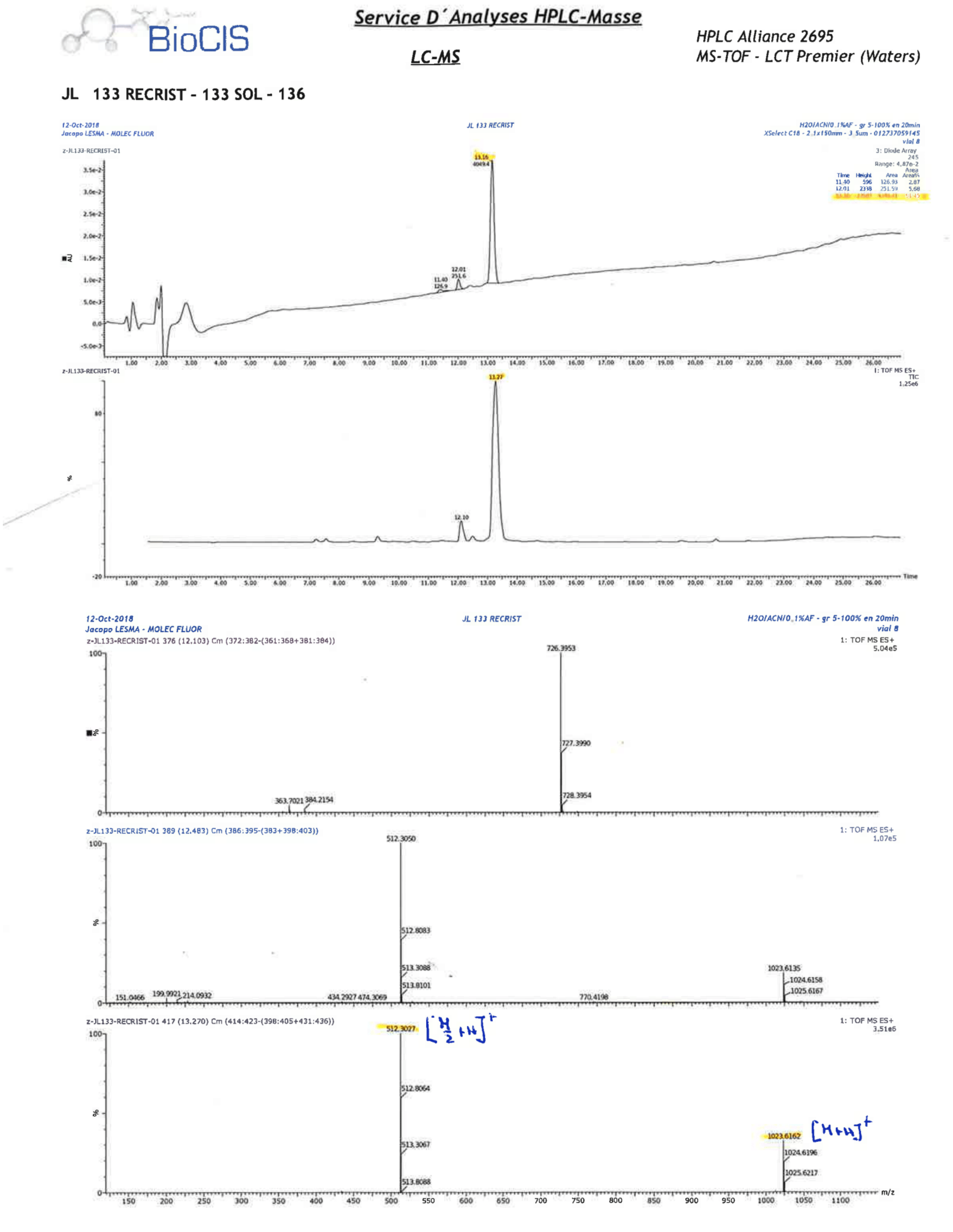

Figure S9 HPLC chromatogram and ESI-MS spectrum of compound **8**.

In the case of the free N-terminal compound **8**, it was not possible to perform a complete assignment of the two major conformers in equilibrium, even by lowering the temperature at 278 K. Increasing the temperature at 313 K, we could approach an approximately complete coalescence of the two conformers, allowing to the attribution of an average conformation. The complete ^{1}H and ^{13}C assignment of the average conformer of **8** was performed (Table S3). The good dispersion of the NH chemical shifts indicated the presence of a single and major conformation whose turn structure was confirmed by ROESY experiments. Several sequential $CH\alpha_i/NH_{i+1}$ ROEs, large $^{3}J_{NH-H\alpha}$ coupling constants and positive ^{1}H CSD values (Table S4) for most of the amino acids (less remarkable for the more dynamic *N*-terminal Ala and *C*-terminal Val), confirmed the extended conformation of the two peptide arms. Diagnostic inter-strand ROEs were observed between the HN of Leu-3 and the H^{α} and H^{β} of Phe-6 and were in favor of a β-hairpin architecture (Fig. S13 and S14). Unfortunately, the characteristic ROEs of the β-turn inducer (Pip4/Pro5) observed for similar compounds (Pellegrino, S., *Chem. Sci.* **2017**, *8*, 1295-1302; Pellegrino, S. *RSC Adv.* **2016**, *6*, 71529 – 71533; Pellegrino, S. *J. Org. Chem.* **2014**, *79*, 3094-3102) and for compound **9** (see below) were not all detected and only the diagnostic ROE $H\alpha_{Pro5}/H2_{Pip4}$ could be observed. In order to study the eventually presence of intramolecular hydrogen-bond, we analyzed the temperature dependence of amide proton chemical shifts, as it can provide information about the hydrogen bond network. No one of the amide protons showed a low temperature coefficient (Table S5). However, Ile-2, Leu-3 and Leu-7 values fell within intermediate values, suggesting their partial involvement in hydrogen-bond. Taking together all these experimental results, it is possible to conclude that two different hairpin architectures, reflecting the same equilibrium of compound **9** (see below), are possible for compound **8**. At 313 K, we can assert that the average conformation of **8** adopts a partial β-hairpin conformation, with the N-terminal Ala residue not in contact with the C-terminal acetyl amide group, that would have allowed to form a more extended β-hairpin.

**Residue**	**δ NH (ppm)**	**δ H^{α} (ppm) ^{3}J (Hz)**	**δ H^{β} (ppm)**	**δ other protons (ppm)**	**δ CO (ppm)**	**δ C^{α} (ppm)**	**δ C^{β} (ppm)**	**δ other carbons (ppm)**
**Ala-1**	8.16	3.98	1.47	/	169.7	48.9	16.5	/
**Ile-2**	8.28	4.24 (8.0 Hz)	1.81	γ_{CH3} 0.92<br>γ_{CH2} 1.18<br>δ_{CH3} 0.86	171.9	58.3	36.5	γ_{CH3} 14.4<br>γ_{CH2} 24.6<br>δ_{CH3} 9.9
**Leu-3**	8.37<br>8.52	4.46 (10.5 Hz)	1.64	γ_{CH} 1.66<br>δ_{CH3} 0.94/0.90	174.7	52.4	40.6	γ_{CH} 24.6<br>δ_{CH3} 20.1
**Pip.-4**	/	/	/	$H_{2/2'}$ 3.15/3.79<br>H_3 3.53<br>H_4 3.65<br>$H_{5/5'}$ 1.17/1.54<br>$H_{6/6'}$ 2.48/4.28	/	/	/	$C_{2/2'}$ 43.4<br>H_3 71.4<br>H_4 51.6<br>$H_{5/5'}$ 24.6<br>$H_{6/6'}$ 40.7
**Pro-5**	/	4.80	1.29/1.96	γ 1.63<br>δ 2.96/3.10	173.3	47.2	41.8	γ 40.6<br>δ 39.0
**Phe-6**	8.28<br>8.77	4.77 (11.3 Hz)	2.97/3.24	H_o 7.29<br>H_m 7.26	171.7	55.2	37.6	C_q 137.0<br>C_o 129.1

		4.77 (16.3 Hz)		H_p 7.18				C_m 128.2 C_p 126.7
**Leu-7**	8.29	4.48 (11.6 Hz)	1.64	γ_{CH} 1.66 δ_{CH3} 0.94/0.90	173.3	52.4	40.6	γ_{CH} 24.6 δ_{CH3} 20.1
**Val-8**	7.79	4.21 (11.8 Hz)	2.06	0.92/0.96	174.6	53.2	30.9	20.8
**Tosyl**	7.74	/	/	H_o 7.83 H_m 7.45 CH_3 2.45	/	/	/	C_{qs} 137.9 C_o 126.9 C_m 129.9 C_{qm} 144.4 CH_3 20.2

Table S3 ^{1}H-NMR and ^{13}C-NMR chemical shifts for the mean conformer of hairpin **8** (14.6 mM) in CD_3OH at 313K

Residue	δ NH random coil	δ NH experimental	NH CSD	δ H^α random coil	δ H^α experimental	H^α CSD
**Ala-1**	8.24	8.16	- 0.08	4.32	3.98	- 0.34
**Ile-2**	8.00	8.28	+ 0.28	4.17	4.24	+ 0.07
**Leu-3**	8.16	8.37<br>8.52	+ 0.21<br>+ 0.36	4.34	4.46<br>4.46	+ 0.12<br>+ 0.12
**Pro-5**	/	/	/	4.42	4.80	+ 0.38
**Phe-6**	8.30	8.28<br>8.77	- 0.02<br>+ 0.47	4.62	4.77<br>4.77	+ 0.15<br>+ 0.15
**Leu-7**	8.16	8.29	+ 0.13	4.34	4.48	+ 0.14
**Val-8**	8.03	7.79	- 0.24	4.12	4.21	+ 0.09
**Residue**	**δ C^α random coil**	**δ C^α experimental**	**C^α CSD**	**δ C=O random coil**	**δ C=O experimental**	**C=O CSD**
**Ala-1**	52.5	48.9	-3.6	177.8	169.7	-8.1
**Ile-2**	61.1	58.3	-2.8	176.4	171.9	-4.5
**Leu-3**	55.1	52.4	-2.7	177.6	174.7	-2.9
**Pro-5**	63.3	47.2	-16.1	177.3	173.3	-4.0
**Phe-6**	57.7	55.2	-2.5	175.8	171.7	-4.1
**Leu-7**	55.1	52.4	-2.7	177.6	173.3	-4.3
**Val-8**	62.2	53.2	-9.0	176.3	174.6	-1.7

Table S4 Chemical shift deviations (CSD) of HN, Hα, Cα and CO for compound **8**

Residue	δ HN (ppm 313K)	δ HN (ppm 278K)	Δδ/ΔT (ppb K^{-1})
**Ala1**	8.16	/	/
**Ile2**	8.28	8.50	-6.29
**Leu3**	8.37<br>8.52	8.58<br>8.75	-6.00<br>-6.57
**Pro5**	/	/	/
**Phe6**	8.28<br>8.77	8.61<br>9.07	-9.43<br>-8.57
**Leu7**	8.29	8.47	-5.14
**Val8**	7.79	8.18	-11.14
**Tos**	7.75	7.94	-5.43

Table S5 Temperature coefficients of HN (Δδ/ΔT) for compound **8**

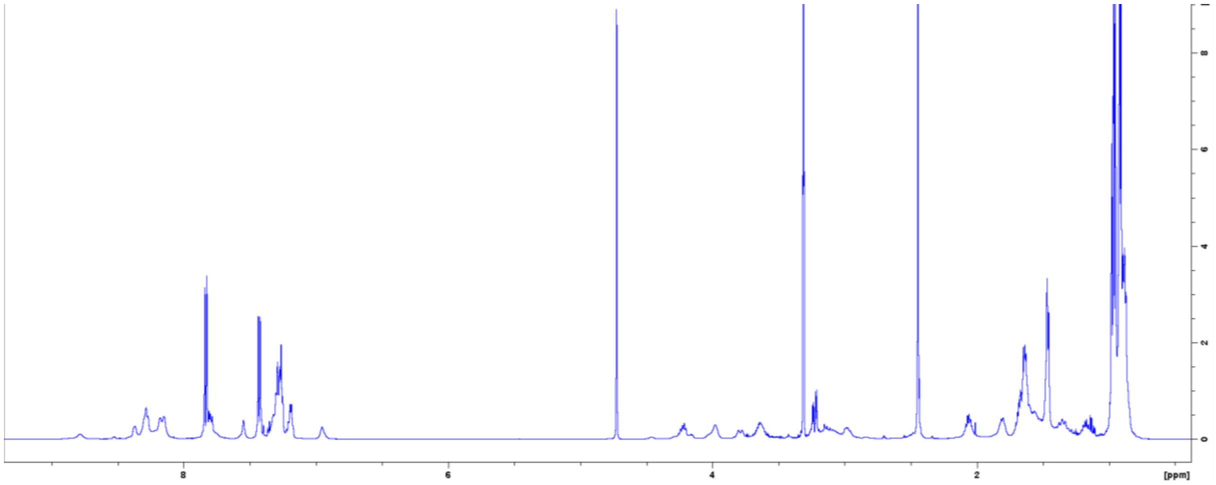

Figure S10 1D ^{1}H-NMR of compound **8** in CD_3OH at 313K

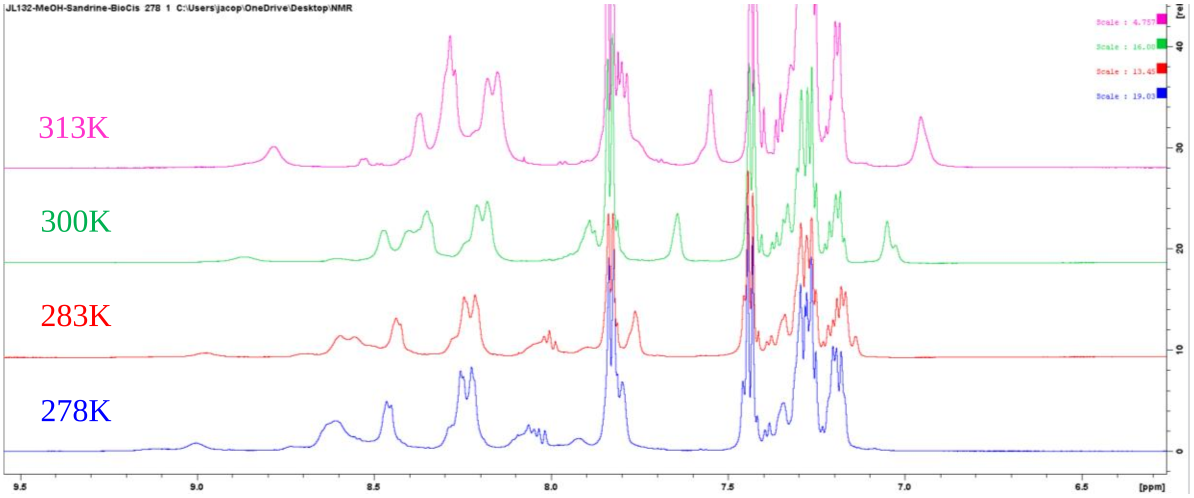

Figure S11 ^{1}H-NMR amide region spectra at different temperatures from 278K to 313K in CD_3OH for compound **8**

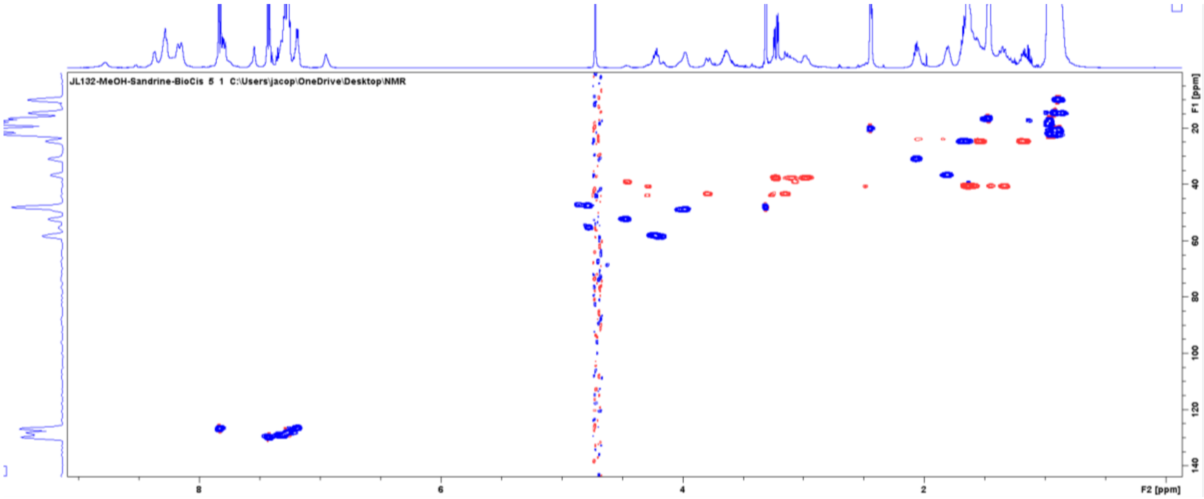

Figure S12 2D ^{13}C-^{1}H HSQC of compound **8** at 313K in CD_3OH

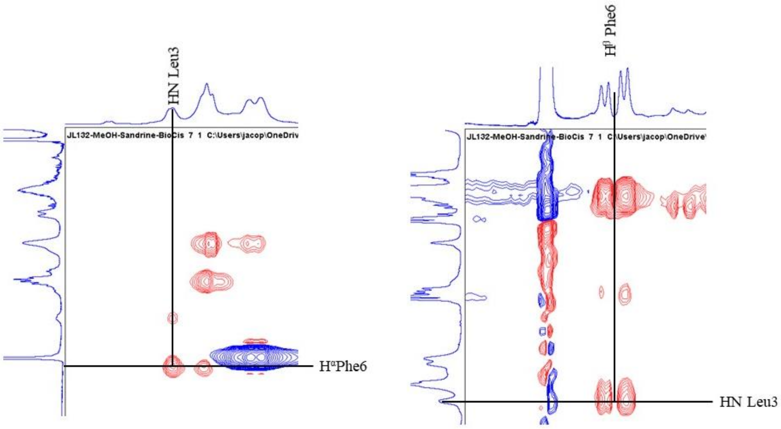

Figure S13 Expansion of the 2D ^{1}H-^{1}H ROESY of compound **8** showing the inter-strand ROEs

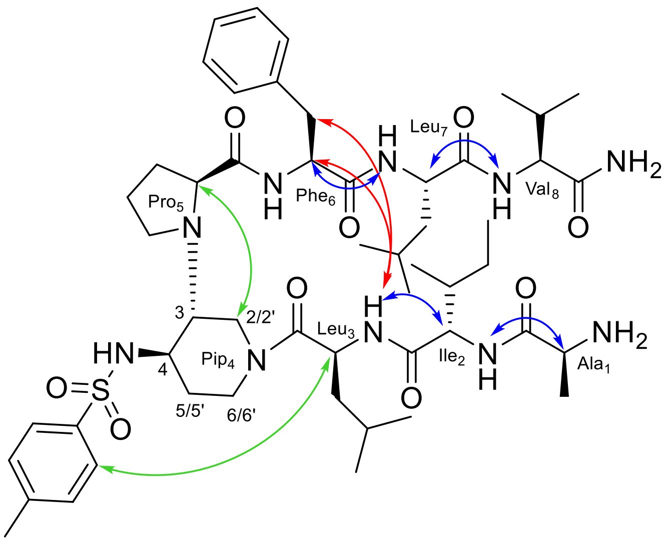

Figure S14 Structure of hairpin **8** showing the assigned ROEs: in blue the sequential CHα$_i$/NH$_{i+1}$ ROEs; in red the inter-strand ROEs, in black the β-turn inducer ROEs and in green other significant ROEs

Compound [9]

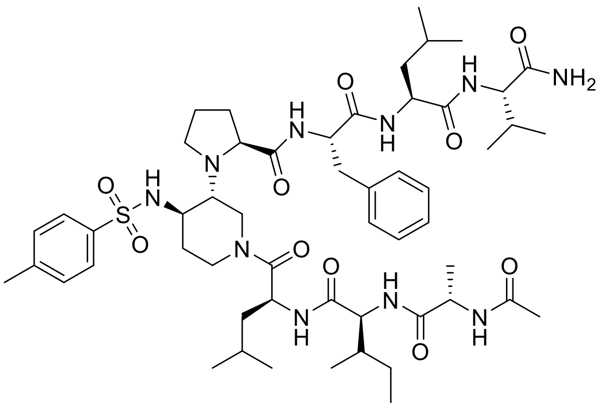

To a stirred solution of **8** (40 mg, 39 µmol, 1 eq.) in dry THF (2 mL) TEA (28 µL, 199 µmol, 5.0 eq.) and acetic anhydride (11 µL, 0.117 µmol, 3.0 eq.) were successively added. The reaction mixture was stirred at 40°C for 6 h. After concentration under reduced pressure, the residue obtained was purified by column chromatography on silica gel eluting with CH_2Cl_2 up to CH_2Cl_2/MeOH (90:10) to afford compound **9** (41.5 mg, 39 µmol, quant.).

Molecular weight = 1064.61 g mol^{-1}
Rf = 0.2 (CH_2Cl_2 /MeOH 95:5)
HRMS: Calcd. for [$C_{54}H_{84}N_{10}O_{10}S$ + H]$^+$: m/z 1065.6126, found: 1065.6184 g mol^{-1}
IR: 3273; 2959; 1640; 1519; 1454 cm^{-1}
HPLC purity: XBridge C18 3.5 µm; H2O + 0:2 % form. ac./ACN, gradient 5–100 % in 20 min; R$_t$ = 16.33 min, 100 %

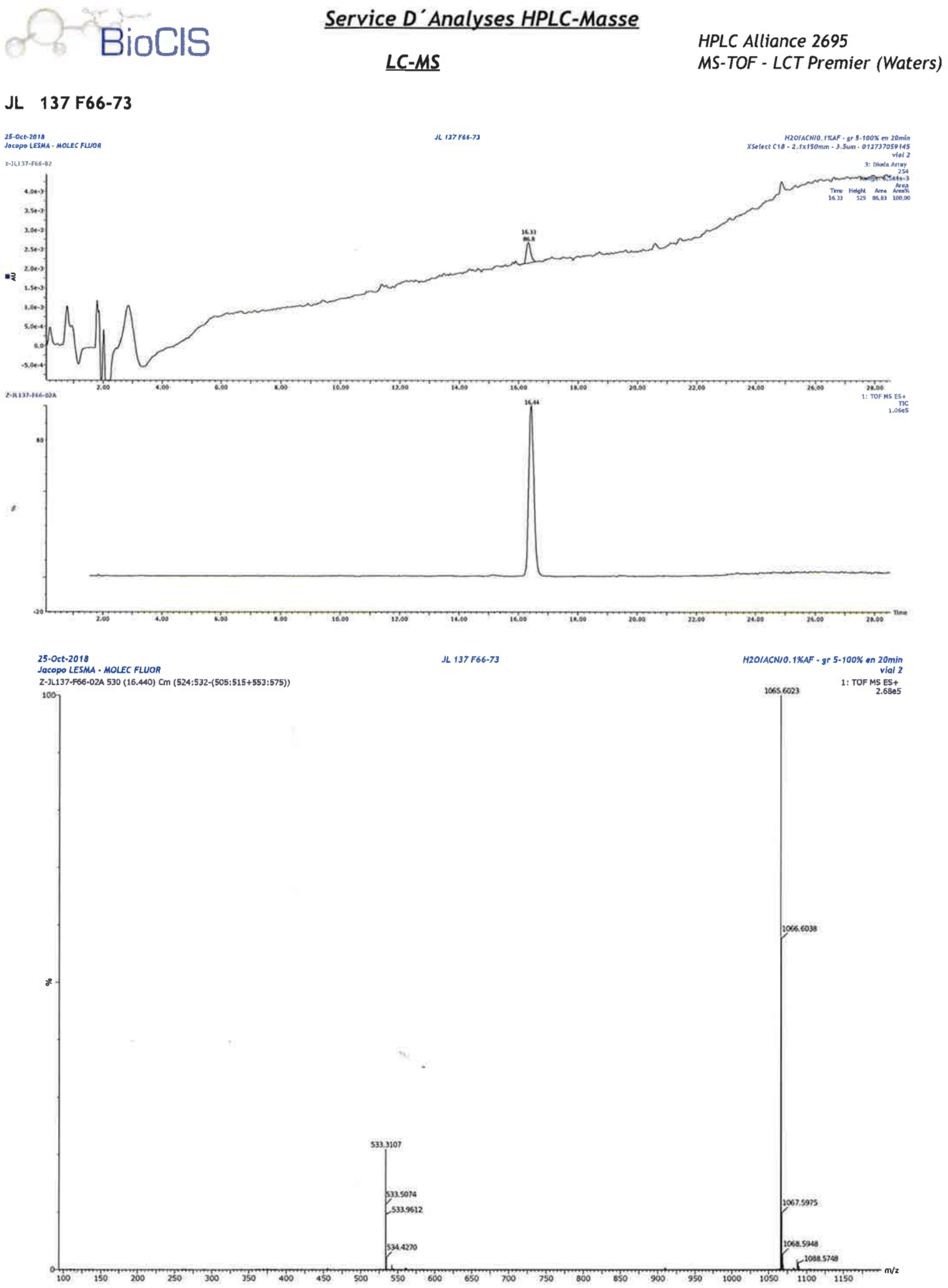

Figure S15 HPLC chromatogram and ESI-MS spectrum of compound **9**.

Complete assignment of the ^{1}H and ^{13}C signals was performed for the two conformers of compound **9** at 278 K (Tables S6 and S7). At this temperature, an equilibrium between two major

conformers (ratio 1:1, **9a**/**9b**) was observed. We hypothesized that we were faced with two different β-hairpin structures, characterized by a different alignment of the two peptide arms. First, we tried to determine the secondary structures of both conformers, by taking structural information coming from through-bond (J coupling) or through space (ROE) magnetization transfer. Large vicinal ^{3}JNH-H_α constants (>8.0 Hz) were observed in both cases, suggesting the presence of dihedral angles typical of peptide segments in extended conformation. Several sequential $CH\alpha_i/NH_{i+1}$ ROEs, indicating β-conformations, were found for both **9a** and **9b** conformers. The extended conformation of the two peptide arms was definitively confirmed by the positive difference between experimental Hα and HN chemical shift values (CSDs) and "random ones" (Tables S8 and S9). Pro-5 in both conformers was characterized by a negative Hα CSD value, thus confirming the folding in a β-hairpin conformation, induced by the piperidine-pyrrolidine β-turn mimic. The very slight differences in values of ^{3}JNH-Hα and of CSD let consider that the peptide arms are similarly extended in both conformers **9a** and **9b**.

The temperature dependence (Δδ/ΔT) of amide proton chemical shifts were not determined for some NH because of overlapping (Fig. S17, Tables S10 and S11). However, the NH of Phe-6 was clearly engaged in hydrogen-bond in both **9a** and **9b** (-2.9 and -3.7 ppb K^{-1}) while of Leu-7 was partially involved in hydrogen-bond (-4.28 ppb K^{-1}) only in **9b**. These observations let hypothesize the presence of a stable β-turn stabilized by a hydrogen bond between Phe-6 (NH) and Leu-3 (CO) in both conformers and a more compact turn structure for **9b** than **9a** due to a possible another hydrogen bond involving the NH of Leu-7.

ROESY experiments confirmed the presence of the β-turn in both conformers. Spatial proximities were found between the Hα of Pro-5 and the H3 and H2 diastereotopic protons of the piperidine ring and between the H3 of Pip-4 with the HN of Phe-6. These ROEs showed that a β-turn conformation occurs in both conformers and that the dihedral angle between the two rings of the turn mimetic remains unchanged, thus rejecting the possible hypothesis of an equilibrium due to the free rotation of the C_{3pip}-N_{Pro} single bond. We also hypothesized that the equilibrium could be ruled by a different chair conformation of the piperidine ring, which might affect the hairpin architecture. This hypothesis has been excluded because in both conformers the same ROEs pattern between the diastereotopic protons have been found (Tables S12 and S13 Fig. S19, S20 and S21), confirming the trans and axial configuration of H3 and H4. The large ^{3}J value (25.8 Hz for **9a** and 26.8 Hz for **9b**) of H3 and H4, reflecting a large dihedral angle between the two protons, corroborates that in both conformers the Pro-5 and the Tosyl group are in equatorial position.

We could detect β-hairpin diagnostic ROEs only for **9b** isomer (Fig. S22 and S23). We observed spatial proximity between the two peptide arms and in particular between the Hα of Ile-2 and the aromatic ring of Phe-6 and the CH_3 of Leu-7, between the Hα of Leu-7 and the CH_3 of Ile-2 and, finally, between the side chains of Ile-2 and Leu-7. This difference let us hypothesize a dynamic equilibrium between two different β-hairpin architectures, with only one of them allowing the spatial proximity between the peptide arms.

Residue	δ NH (ppm)	δ H^{α} (ppm) ^{3}J (Hz)	δ H^{β} (ppm)	δ other protons (ppm)	δ CO (ppm)	δ C^{α} (ppm)	δ C^{β} (ppm)	δ other carbons (ppm)
**Ala-1**	8.33	4.33<br>(6.9 Hz)	1.28	/	173.8	49.2	16.8	/
**Ile-2**	8.08	4.15<br>(10.7 Hz)	1.71	γ_{CH3} 0.83<br>γ_{CH2} 1.06<br>δ_{CH3} 0.73	172.1	57.4	36.9	γ_{CH3} 10.2<br>γ_{CH2} 24.6<br>δ_{CH3} 14.9
**Leu-3**	8.37	4.80<br>(9.1 Hz)	1.29/1.59	γ_{CH} 1.45<br>δ_{CH3} 0.87	170.7	46.7	40.6	γ_{CH} 24.6<br>δ_{CH3} 20.1
**Pip.-4**	/	/	/	$H_{2/2'}$ 2.75/4.45<br>H_3 2.22<br>H_4 3.63<br>$H_{5/5'}$ 1.24/1.66<br>$H_{6/6'}$ 3.03/3.79	/	/	/	$C_{2/2'}$ 39.6<br>H_3 59.3<br>H_4 53.9<br>$H_{5/5'}$ 32.7<br>$H_{6/6'}$ 44.2
**Pro-5**	/	3.34	1.30/1.93	γ 1.56<br>δ 2.61/3.09	176.0	63.4	30.8	γ 40.6<br>δ 44.5
**Phe-6**	8.59	4.76<br>(9.6 Hz)	3.17	H_o 7.36<br>H_m 7.26<br>H_p 7.20	173.4	54.1	37.3	C_q 137.5<br>C_o 129.2<br>C_m 128.1<br>C_p 126.4
**Leu-7**	8.74	4.54<br>(10.2 Hz)	1.58/1.66	γ_{CH} 1.60<br>δ_{CH3} 0.95	173.5	51.8	40.6	γ_{CH} 24.6<br>δ_{CH3} 20.1
**Val-8**	8.27	4.14<br>(8.2 Hz)	2.06	γ_{CH} 0.96<br>amide 7.14 or 7.18 and 7.76	174.7	58.7	30.7	18.47
**Tosyl**	7.69	/	/	H_o 7.82<br>H_m 7.41<br>CH_3 2.45	/	/	/	C_{qs} 139.7<br>C_o 126.6<br>C_m 129.8<br>C_{qm} 143.5<br>CH_3 20.2
**Acetyl**	/	/	/	CH_3 1.95	171.7	/	/	CH_3 21.1

Table S6 ^{1}H and ^{13}C NMR chemical shifts for the conformer **9a** (37.5 mM) in CD_3OH at 278K

Residue	δ NH (ppm)	δ H^{α} (ppm) ^{3}J (Hz)	δ H^{β} (ppm)	δ other protons (ppm)	δ CO (ppm)	δ C^{α} (ppm)	δ C^{β} (ppm)	δ other carbons (ppm)
**Ala-1**	8.31	4.39<br>(6.5 Hz)	1.28	/	173.8	46.1	16.8	/
**Ile-2**	8.09	4.15<br>(10.7 Hz)	1.77	γ_{CH3} 0.87<br>γ_{CH2} 1.12<br>δ_{CH3} 0.73	171.6	57.4	36.6	γ_{CH3} 10.2<br>γ_{CH2} 24.6<br>δ_{CH3} 14.9
**Leu-3**	8.34	4.86<br>(8.09 Hz)	1.46/1.53	γ_{CH} 1.67<br>δ_{CH3} 0.86	171.3	47.3	40.5	γ_{CH} 24.6<br>δ_{CH3} 20.8
**Pip.-4**	/	/	/	$H_{2/2'}$ 3.26/4.23<br>H_3 2.49<br>H_4 3.57<br>$H_{5/5'}$ 1.19/1.52	/	/	/	$C_{2/2'}$ 43.9<br>H_3 60.2<br>H_4 54.0<br>$H_{5/5'}$ 31.7

				$H_{6/6'}$ 2.48/4.28				$H_{6/6'}$ 40.9
**Pro-5**	/	3.34	1.30/1.93	γ 1.56<br>δ 2.61/3.09	176.0	62.6	30.8	γ 40.7<br>δ 44.5
**Phe-6**	8.67	4.91<br>(10.1 Hz)	3.12/3.21	H_o 7.36<br>H_m 7.26<br>H_p 7.20	172.8	54.1	37.3	C_q 137.5<br>C_o 129.2<br>C_m 128.1<br>C_p 126.4
**Leu-7**	8.50	4.54<br>(10.2 Hz)	1.66/1.58	γ_{CH} 1.60<br>δ_{CH3} 0.91/0.97	173.3	51.8	40.6	γ_{CH} 24.6<br>δ_{CH3} 20.1
**Val-8**	8.11	4.21<br>(9.1 Hz)	2.06	γ_{CH} 0.96<br>amide 7.14 or<br>7.18 and 7.76	173.3	58.7	30.8	18.47
**Tosyl**	7.87	/	/	H_o 7.82<br>H_m 7.41<br>CH_3 2.45	/	/	/	C_{qs} 139.7<br>C_o 126.6<br>C_m 129.8<br>C_{qm} 143.5<br>CH_3 20.2
**Acetyl**	/	/	/	CH_3 1.95	171.9	/	/	CH_3 21.1

Table S7 ^{1}H and ^{13}C NMR chemical shifts for the conformer **9b** (37.5 mM) in CD_3OH at 278K

**Residue**	**δ NH random coil**	**δ NH experimental**	**NH CSD**	**δ H^α random coil**	**δ H^α experimental**	**H^α CSD**
**Ala-1**	8.24	8.33	+ 0.09	4.32	4.33	- 0.01
**Ile-2**	8.00	8.08	+ 0.08	4.17	4.15	- 0.02
**Leu-3**	8.16	8.37	+ 0.21	4.34	4.80	+ 0.46
**Pro-5**	/	/	/	4.42	3.34	- 1.08
**Phe-6**	8.30	8.59	+ 0.29	4.62	4.76	+ 0.14
**Leu-7**	8.16	8.74	+ 0.58	4.34	4.54	+ 0.20
**Val-8**	8.03	8.27	+ 0.24	4.12	4.14	+ 0.02
**Residue**	**δ C^α random coil**	**δ C^α experimental**	**C^α CSD**	**δ C=O random coil**	**δ C=O experimental**	**C=O CSD**
**Ala-1**	52.5	49.2	-3.3	177.8	173.8	-4.0
**Ile-2**	61.1	57.4	-3.7	176.4	172.1	-4.3
**Leu-3**	55.1	46.7	-8.4	177.6	170.7	-6.9
**Pro-5**	63.3	30.8	-32.5	177.3	176.0	-1.3
**Phe-6**	57.7	37.3	-20.4	175.8	173.4	-2.4
**Leu-7**	55.1	40.6	-14.5	177.6	173.5	-4.1
**Val-8**	62.2	30.7	-31.5	176.3	174.7	-1.6

Table S8 Chemical shift deviations (CSD) of HN, H^α, C^α and CO, for conformer **9a**

**Residue**	**δ NH random coil**	**δ NH experimental**	**NH CSD**	**δ H^α random coil**	**δ H^α experimental**	**H^α CSD**
**Ala-1**	8.24	8.31	+ 0.07	4.32	4.39	+0.07
**Ile-2**	8.00	8.09	+ 0.09	4.17	4.15	- 0.02
**Leu-3**	8.16	8.34	+ 0.18	4.34	4.86	+ 0.52
**Pro-5**	/	/	/	4.42	3.75	- 0.67
**Phe-6**	8.30	8.67	+0.37	4.62	4.91	+ 0.29
**Leu-7**	8.16	8.50	+ 0.34	4.34	4.54	+ 0.20
**Val-8**	8.03	8.11	+ 0.08	4.12	4.21	+ 0.09
**Residue**	**δ C^α random coil**	**δ C^α experimental**	**C^α CSD**	**δ C=O random coil**	**δ C=O experimental**	**C=O CSD**
**Ala-1**	52.5	46.1	-6.4	177.8	173.8	-4.0
**Ile-2**	61.1	57.4	-3.7	176.4	171.6	-4.8

**Leu-3**	55.1	47.3	-7.8	177.6	171.3	-6.3
**Pro-5**	63.3	62.6	-0.7	177.3	176.0	-1.3
**Phe-6**	57.7	54.1	-3.6	175.8	172.8	-3.0
**Leu-7**	55.1	51.8	-3.3	177.6	173.3	-4.3
**Val-8**	62.2	58.7	-3.5	176.3	173.3	-3.0

Table S9 Chemical shift deviations (CSD) of HN, H^{α}, C^{α} and CO, for conformer **9b**

Residue	δ HN (ppm 313K)	δ HN (ppm 278K)	Δδ/ΔT
**Ala1**	Overlapped	8.33	/
**Ile2**	Overlapped	8.08	/
**Leu3**	Overlapped	8.37	/
**Pro5**	/	/	/
**Phe6**	8.49	8.59	-2.86
**Leu7**	8.50	8.74	-6.86
**Val8**	7.96	8.27	-8.86
**Tos**	Overlapped	7.69	/

Table S10 Temperature coefficients of HN (Δδ/ΔT) for compound **9a**

Residue	δ HN (ppm 313K)	δ HN (ppm 278K)	Δδ/ΔT
**Ala1**	Overlapped	8.31	/
**Ile2**	Overlapped	8.09	/
**Leu3**	Overlapped	8.34	/
**Pro5**	/	/	/
**Phe6**	8.54	8.67	-3.71
**Leu7**	8.35	8.50	-4.28
**Val8**	Overlapped	8.11	/
**Tos**	7.61	7.87	-7.42

Table S11 Temperature coefficients of HN (Δδ/ΔT) for compound **9b**

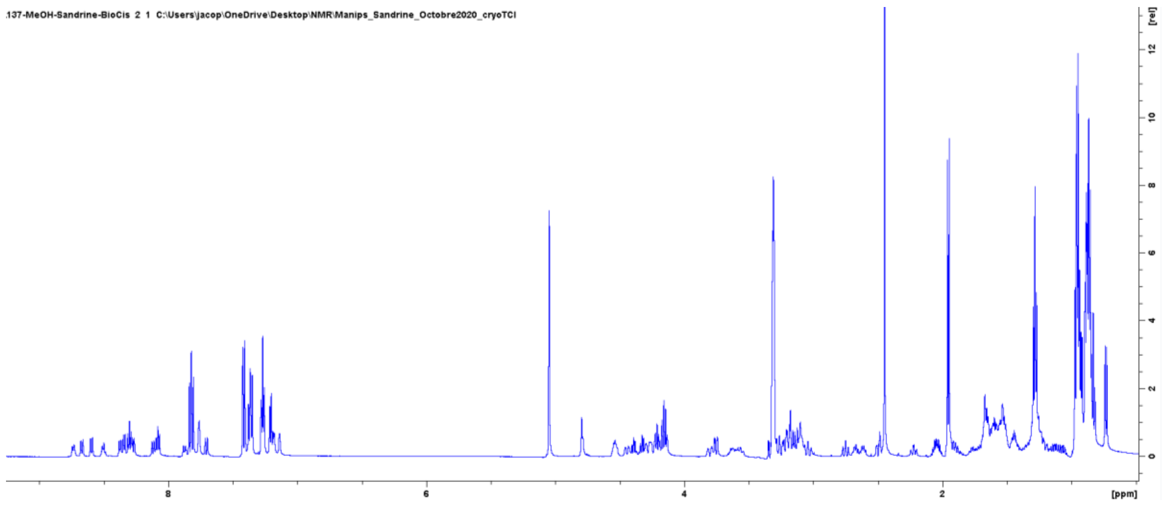

Figure S16 1D ^{1}H-NMR of compound **9** in CD_3OH at 278K

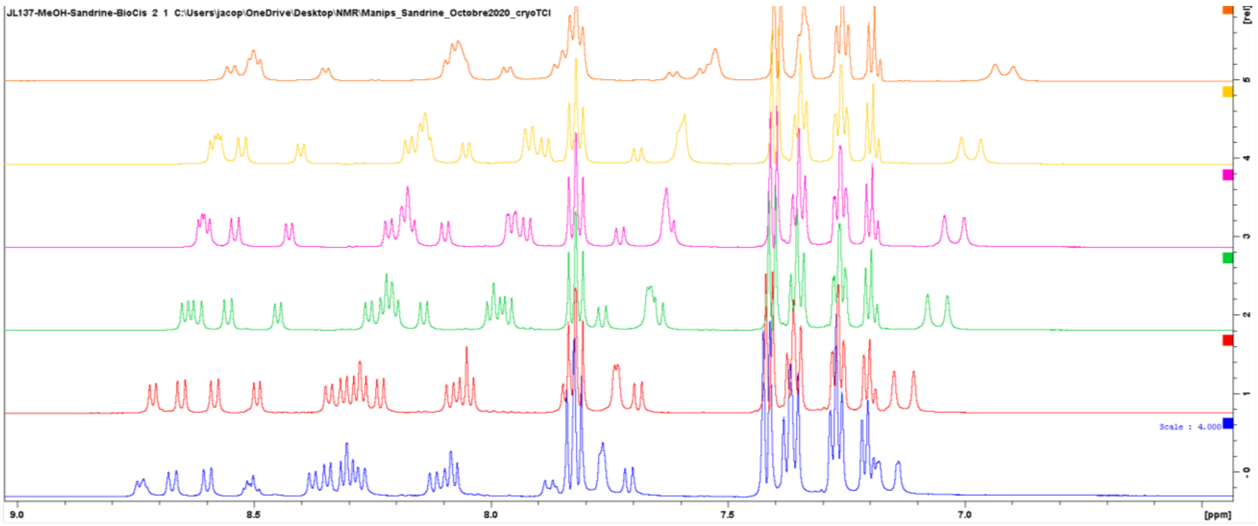

Figure S17 ^{1}H-NMR amide region spectra at different temperatures from 278K to 313K in CD_3OH for compound **9**

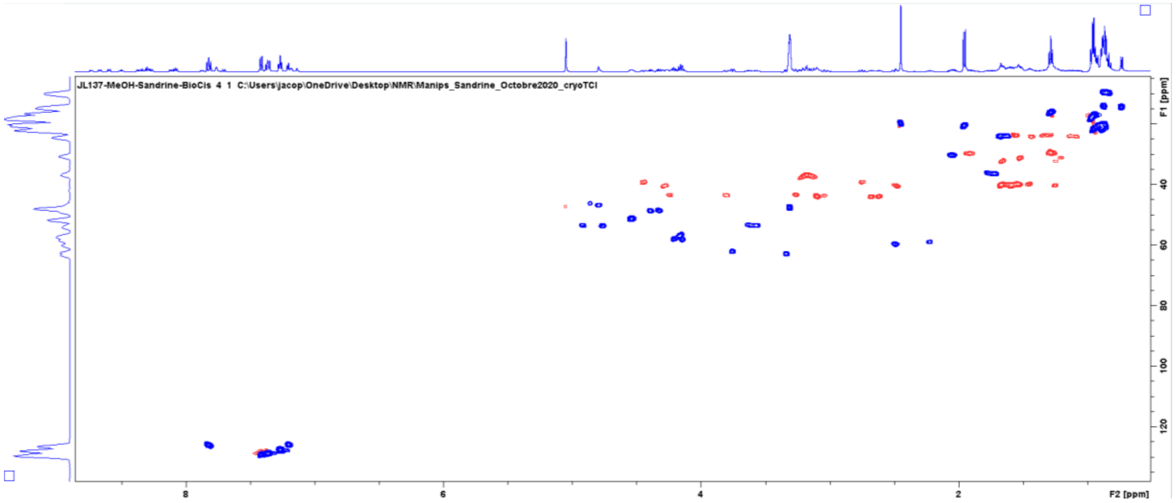

Figure S18 2D ^{13}C-^{1}H HSQC of compound **9** at 278K in CD_3OH

Proton (ppm)	δ CH_2 (2) (ppm)	δ CH_2 (5)(ppm)	δ CH_2 (6)(ppm)
**CH (3) 2.22**	4.45 (H^{eq})	1.24 (H^{ax})	3.79 (H^{eq})
**CH (4) 3.63**	2.75 (H^{ax})	1.66 (H^{eq})	3.03 (H^{ax})

Table S12 Piperidine diastereotopic protons with which H^3 or H^4 establish ROEs in conformers **9a**

Proton (ppm)	δ CH_2 (2) (ppm)	δ CH_2 (5)(ppm)	δ CH_2 (6)(ppm)
**CH (3) 2.49**	4.23 (H^{eq})	1.19 (H^{ax})	4.28 (H^{eq})
**CH (4) 3.57**	3.26 (H^{ax})	1.52 (H^{eq})	2.48 (H^{ax})

Table S13 Piperidine diastereotopic protons with which H^3 or H^4 establish ROEs in conformers **9b**

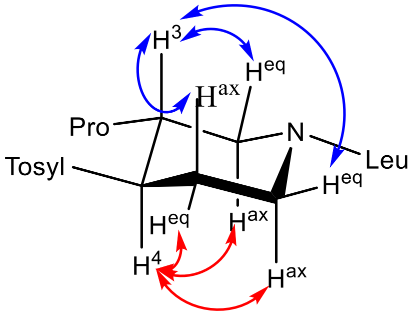

Figure S19 Structure of the piperidine ring showing the common ROE of **9a** and **9b**. In blue the ROEs of H^3 and in red the ROEs of H^4

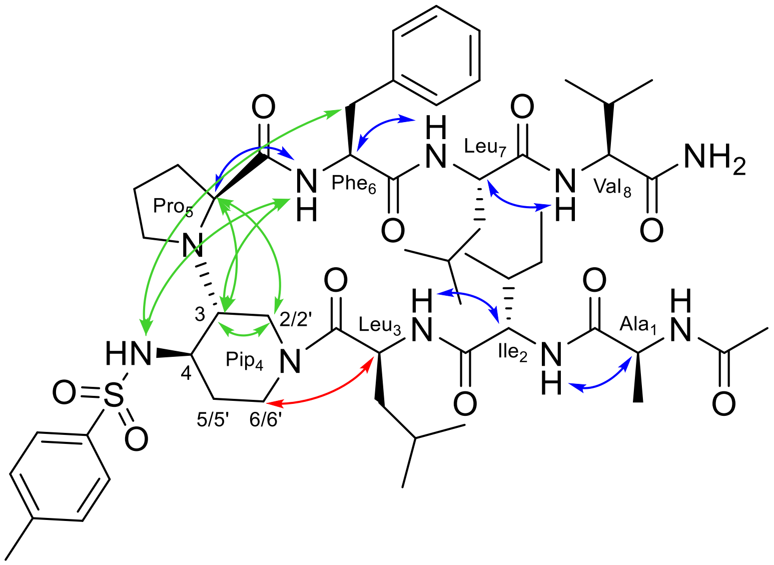

Figure S20 Structure of hairpin **9a** showing the assigned ROEs: in blue the sequential $CH\alpha_i/NH_{i+1}$ ROEs, in green the common ROEs with **9b** in red the differences.

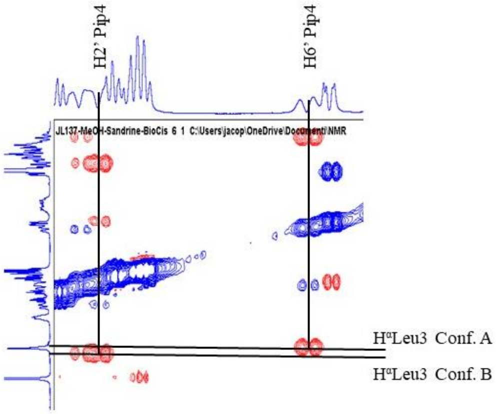

Figure S21 Expansion of the 2D ^1H-^1H ROESY of compound **9** showing the different ROEs between Leu3 and Pip4 for both conformers **9a** and **9b**.

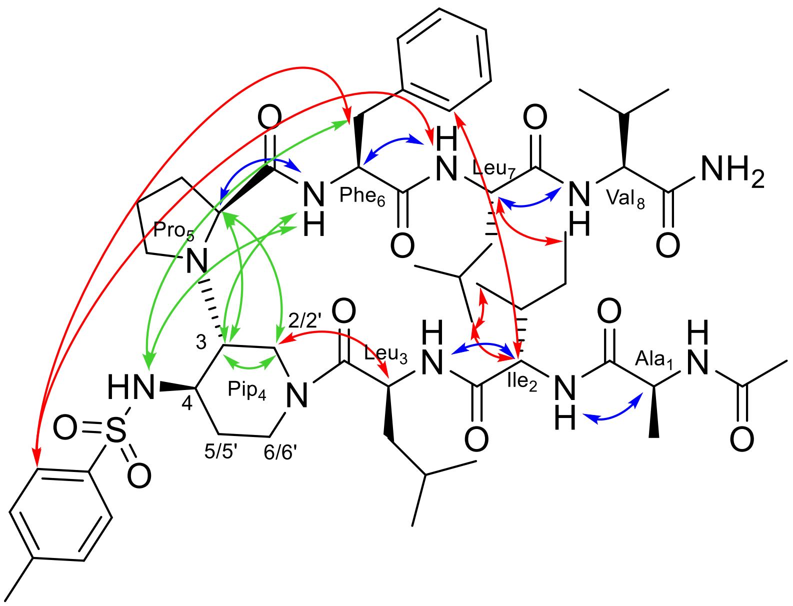

Figure S22 Structure of hairpin **9b** showing the assigned ROEs: in blue the sequential CHαi/NHi+1 ROEs, in green the common ROEs with **9a** in red the differences.

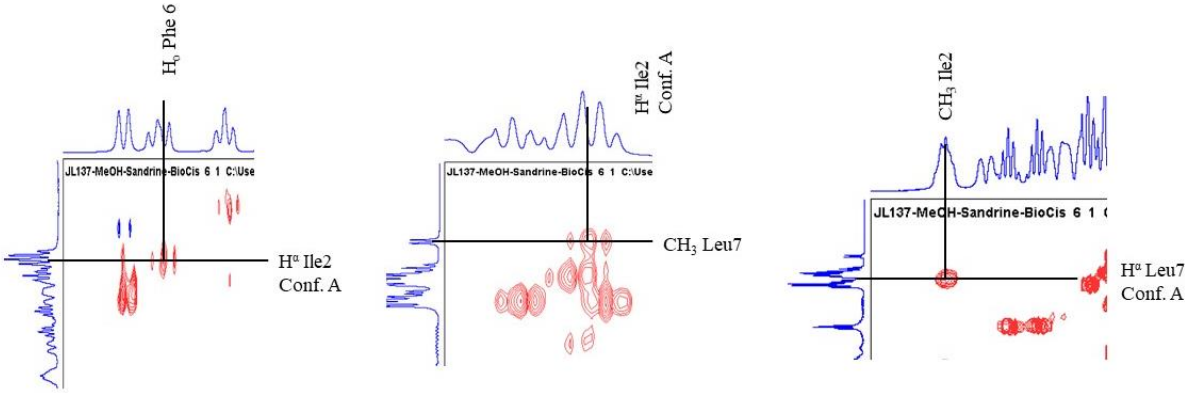

Figure S23 Expansion of the 2D ^{1}H-^{1}H ROESY of compound **9b** showing the inter-strand ROEs

Before analyzing the spatial proximity between the two peptide arms in each conformer, we first checked the stability of the piperidine-proline local structure during the dynamic simulations. As displayed in Fig. S24, the probability distributions of the three torsion angles H3-C3-C4-H4, Cα-N-C3-C2, and Cα-C-N-C2 are unimodal and centered around their initial values, indicating that the piperidine-proline local structure is maintained and no conformational transition of the torsion angle Cα-C-N-C2 between Leu-3 and Pip-4 occured in MD simulations, for both conformers **9a** and **9b**.

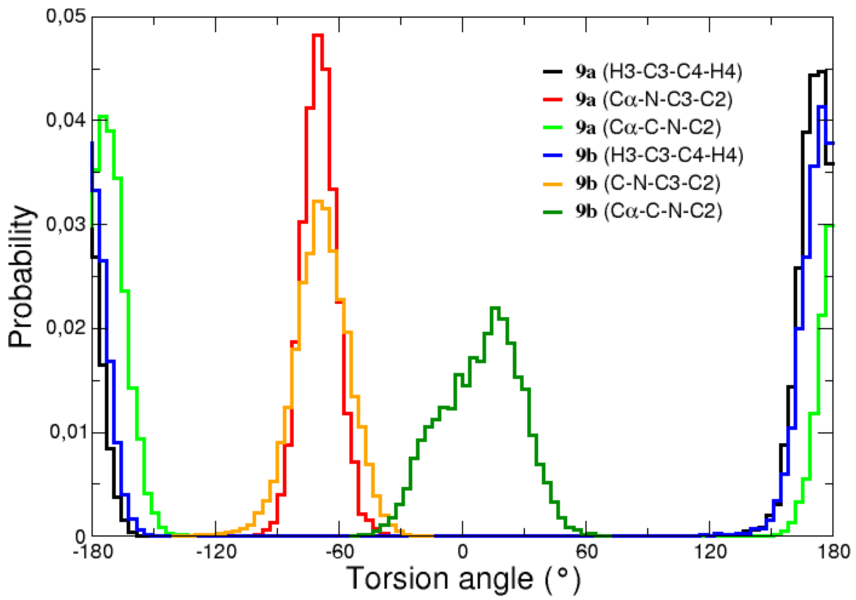

Figure S24 MD-derived probability distribution of the torsion angles H3-C3-C4-H4 of the piperidine cycle, (Pro-5) Cα-N-C3-C2 between the piperidine and proline rings, and (Leu-3) Cα-C-N-C2 connecting Leu-3 to piperidine.

We also calculated the $^{3}J_{HN\text{-}H\alpha}$ coupling constants of the two peptidic arms during simulations and compared them with NMR measurements (Table S14). Overall, the computed $^{3}J_{HN\text{-}H\alpha}$ coupling constants have lower values than NMR data, indicating that the two peptide arms are less extended in MD simulations than in NMR experiments. This is particularly striking for the **9b** peptide arms which exhibit local bent conformations at residues Ile-2 and Phe-6 as indicated by their low J-coupling values (4.9 and 3.3 Hz, respectively) whereas both NMR values are above 10 Hz. These discrepancies (which are also observed but in a lesser extent for conformer **9a**) might be due to imperfections of the GAFF Force Field used here for the non-purely peptide solute and/or the methanol solvent.

	**9a**		**9b**	
	NMR	MD	NMR	MD
**Ala-1**	6.9	5.5	6.5	5.7
**Ile-2**	10.7	5.7	10.7	4.9
**Leu-3**	9.1	8.2	8.1	8.0
**Phe-6**	9.6	5.8	10.1	3.3
**Leu-7**	10.2	5.7	10.2	5.7
**Val-8**	8.2	7.2	9.1	8.5

Table S14 Comparison between NMR-measured and MD-derived $^{3}J_{HN\text{-}H\alpha}$ coupling constants of the 6 amino acids of the two peptidic arms.

Compound [10]

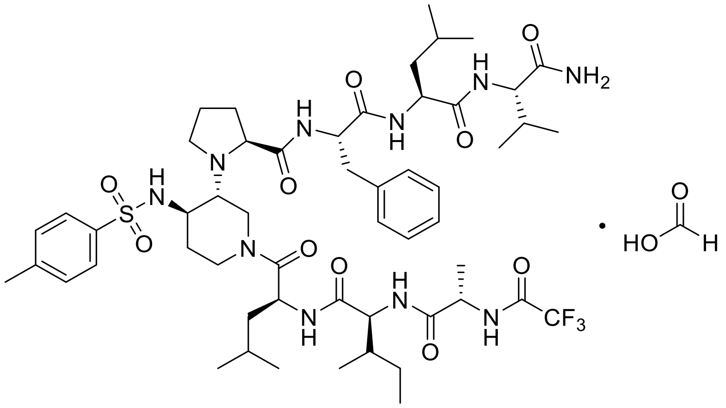

To a stirred solution of **8** (60 mg, 54 µmol, 1 eq.) in dry THF (2mL) at 0°C, TEA (31 µL, 222 µmol, 4.0 eq.) and trifluoroacetic anhydride (11 µL, 0.117 µmol, 3.0 eq.) were successively added. The reaction mixture was then stirred for 6 h at 0°C. After concentration under reduced pressure, the residue was purified by preparative HPLC eluting with H_2O + 0.2% formic acid/CH_3CN (gradient 50 to 100% in 20 min) on a Sunfire column (C18, 4.6 x150mm-5µm) to afford **10** (11 mg, 9.4 µmol, 5%) as a white powder.

Molecular weight = 1165.38 g mol^{-1}; free amine 1118.56 g mol^{-1}
Rf = 0.3 (CH_2Cl_2/MeOH 95:5)
HRMS: Calcd. for $[C_{54}H_{81}F_3N_{10}O_{10}S + H]^+$: m/z 1119.5888, found: 1119.5898 g mol^{-1}
^{1}H NMR (CD_3OH, 600 MHz, 278K): two conformers δ 9.46 (1H, m); 8.74 (0.5H, m); 8.69 (0.5H, d, *J* = 10.4 Hz); 8.60 (0.5H, d, *J* = 10.4 Hz); 8.45-8.35 (1.5H, m); 8.27-8.22 (1H, m); 8.11 (0.5H, d, *J* = 9.2 Hz); 7.91 (0.5, d, *J* = 10.0 Hz); 7.82 (2H, m); 7.77 (1H, m); 7.70 (0.5H, d, *J* = 9.8 Hz); 7.42 (2H, d, *J* = 8.4 Hz); 7.37 (2H, m); 7.27 (2H, m); 7.21 (1H, d, *J* = 7.6 Hz); 7.18 (0.5H, bs); 7.14 (0.5H, bs); 4.90 (0.5H, m); 4.87 (0.5H, m); 4.79 (0.5H, m); 4.76 (0.5H, m); 4.53 (0.5H, m); 4.49-4.39 (1.5H, m); 4.32-4.10 (3H, m); 3.83-3.73 (1H, m); 3.66-3.57 (1H, m); 3.34-3.01 (4.5H, m); 2.76 (0.5H, m); 2.69 (0.5H, m); 2.62 (0.5H, m); 2.53-2.46 (1H, m); 2.45 (3H, s); 2.23 (0.5H, m); 2.05 (1H, m); 1.93 (1H, m); 1.81-1.48 (8.5H, m); 1.47-1.06 (7.5, m); 1.01-0.81 (21H, m); 0.73(3H, d, *J* = 6.9 Hz) ppm
^{13}C NMR (CD_3OH, 150 MHz, 278K): two conformers δ 176.0; 174.7; 173.4; 173.2; 172.9; 172.3); 172.0; 171.4; 171.3; 157.4; 143.5; 139.6; 137.5; 129.7; 129.3; 128.1; 126.6; 126.4; 63.4; 60.3; 59.5; 58.8; 58.5; 57.6; 57.2; 54.1; 54.0; 53.8; 51.8; 49.6; 47.3; 46.7; 44.5; 44.0; 43.8; 41.0; 40.7; 40.6; 40.5; 40.3; 39.7; 37.4; 36.9; 36.6; 31.6; 30.8; 29.5; 24.6; 24.5; 24.1; 21.0; 20.3; 18.0; 17.5; 16.3; 14.9; 10.1 ppm
^{19}F NMR (CD_3OD, 188 MHz, 298K): two conformers δ -77.1; -77.0 ppm
IR: 3291; 2961; 1642; 1541; 1456 cm^{-1}
HPLC purity: XBridge C18 3.5 µm; H_2O + 0:2 % form. ac./ACN, gradient 5–100 % in 20 min; R_t = 13.45 min, 97 %

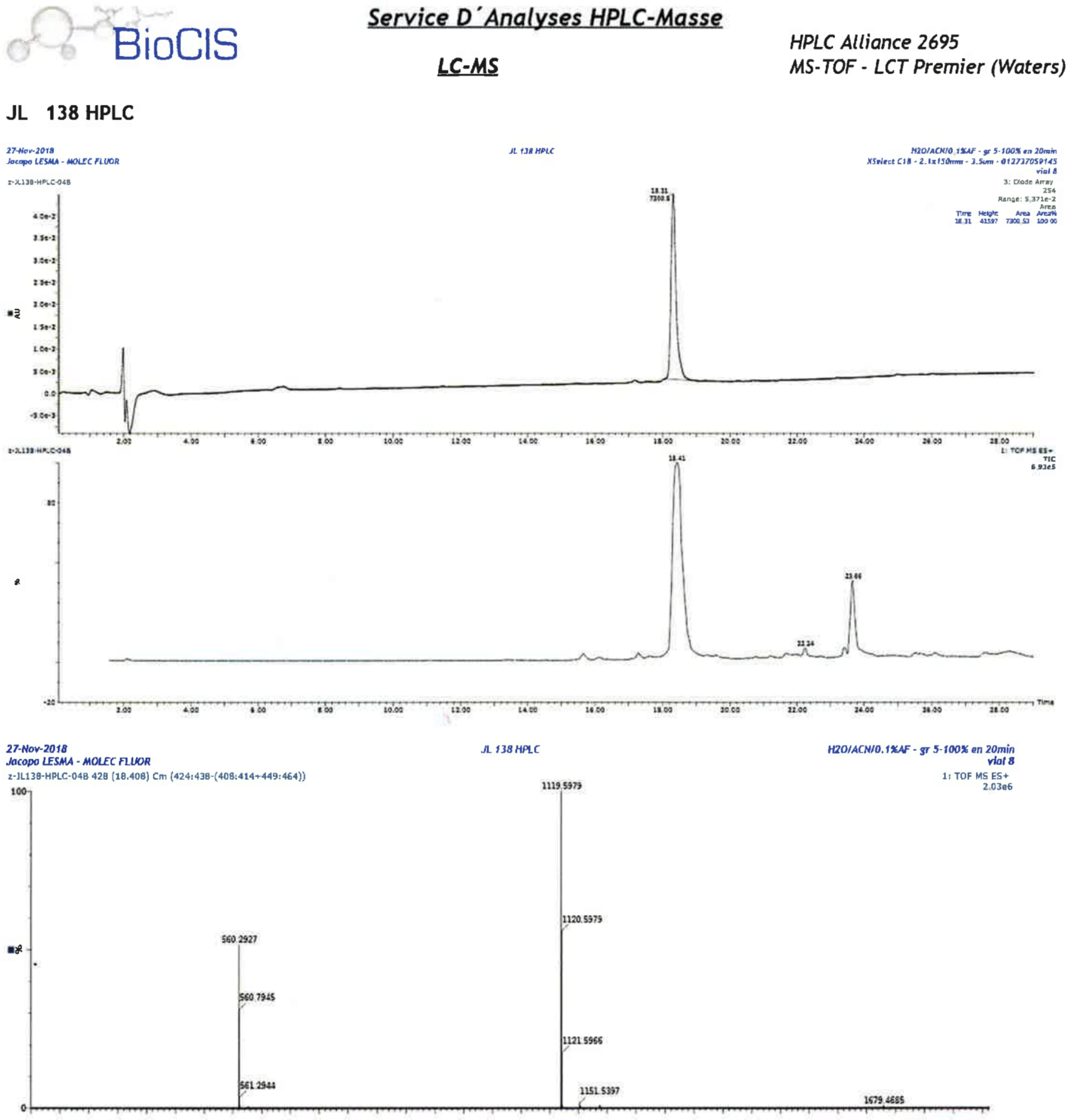

Figure S25 HPLC chromatogram and ESI-MS spectrum of compound **10**.

Compound [13]

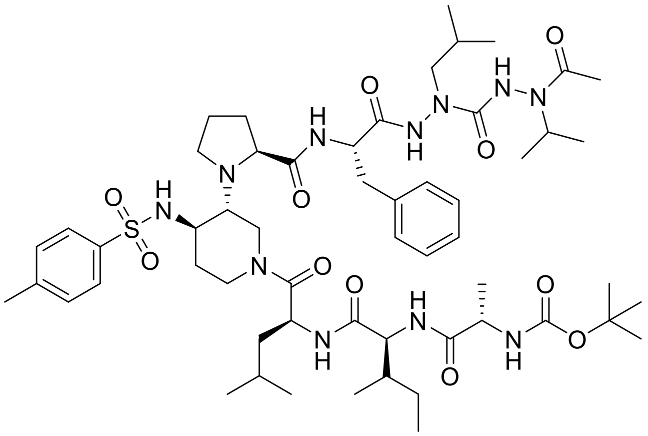

To a solution of compound **19** (0.19 g, 0.25 mmol, 1 eq.) in dry DMF were successively added under argon atmosphere HATU (0.19 g, 0.5 mmol, 2 eq.) and HOAt (0.07 g, 0.5 mmol, 2 eq.) were added at 0°C. A solution of compound **15** (0.10 g, 0.25 mmol, 1 eq.) and DIPEA (0.75 mL, 1.25 mmol, 5 eq.) in dry DMF was added dropwise to the previous mixture at 0°C. Then, the reaction was let 2 days at 60°C under argon atmosphere. After removal of the volatile under reduced pressure, the crude oil was taken up with EtOAc, washed with distilled water, saturated $NaHCO_3$ and brine, dried over $MgSO_4$, filtered and concentrated under reduced pressure. The crude residue obtained was purified by column chromatography on silica gel eluting with EtOAc as eluent to afford **13** as a white powder (0.16 g, 0.15 mmol, 59 %). The desired compound **13** was obtained together with a compound resulting from the intramolecular cyclization between the carboxylic group and the NH of the sulphonamide of **19** (Fig. S28).

Molecular weight = 1123.65 g mol^{-1}

R*f* = 0.45 (EtOAc)

HRMS: Calcd. for $[C_{56}H_{89}N_{11}O_{11}S+H]^+$: M/z 1124.6542, found: 1124.6511

^{1}**H NMR** (DMSO-*d6,* 400 MHz, 298 K): *δ* 10.44 (1H, bs); 8.78 (1H, bs); 8.40 (1H, bs); 8.04 (1H, bs); 7.74 (2H, m); 7.50 (1H, bs); 7.46 (1H, bs); 7.39 (2H, m); 7.26 (4H, m); 7.20 (1H, m); 7.00 (1H, bs); 4.66 (1H, bs); 4.54 (1H, m); 4.50 (1H, m); 4.48 (1H, m); 4.25 (2H, m); 4.16 (1H, m); 3.96 (1H, bs); 3.27 (1H, m); 3.69 (1H, m); 3.26 (1H, m); 3.16 (1H, m), 3.01 (1H, m), 3.00 (1H, m); 2.98 (1H, m); 2.97 (1H, m), 2.69 (1H, m); 2.54 (2H, m); 2.39 (3H, s); 1.96 (1H, m), 1.76 (1H, m); 1.73 (3H, s); 1.65 (1H, m); 1.46 (2H, m); 1.55 (1H, m); 1.38 (1H, m); 1.36 (9H, s); 1.26 (2H, m); 1.23 (2H, m); 1.12 (3H, m); 1.05 (1H, m); 1.02 (3H, d, *J* = 6.3 Hz); 0.95 (3H, d, *J* = 6.7 Hz); 0.90 (6H, d, *J* = 6.9 Hz); 0.84 (3H, m); 0.82 (3H, m); 0.78 (3H, m); 0.77 (3H, m) ppm

^{13}**C NMR** (DMSO-*d6*, 100 MHz, 298 K): *δ* 175.0; 172.7; 172.2; 172.0; 170.2; 170.8; 162.2, 142.8; 139.1; 138.1; 136.7; 129.6; 129.3; 128.1; 126.5; 78.0; 62.6; 56.4; 53.0; 49.7; 46.8; 46.4; 44.7; 43.2, 43.0; 40.5; 39.5; 38.1; 36.9; 36.8; 30.4; 28.9; 28.1; 24.1; 23.1; 21.5; 21.0; 20.9; 20.8; 19.5; 19.1; 19.0; 17.7; 15.2; 10.9 ppm

Melting point = 164-168°C

IR: 3277; 2962; 1639; 1518, 1444; 1251; 1159 cm^{-1}

HPLC purity: XBridge C18 3.5 μm; H_2O + 0.1 % form. ac./ACN, gradient 5–100 % in 20 min; R_t = 20.08 min, 92 %

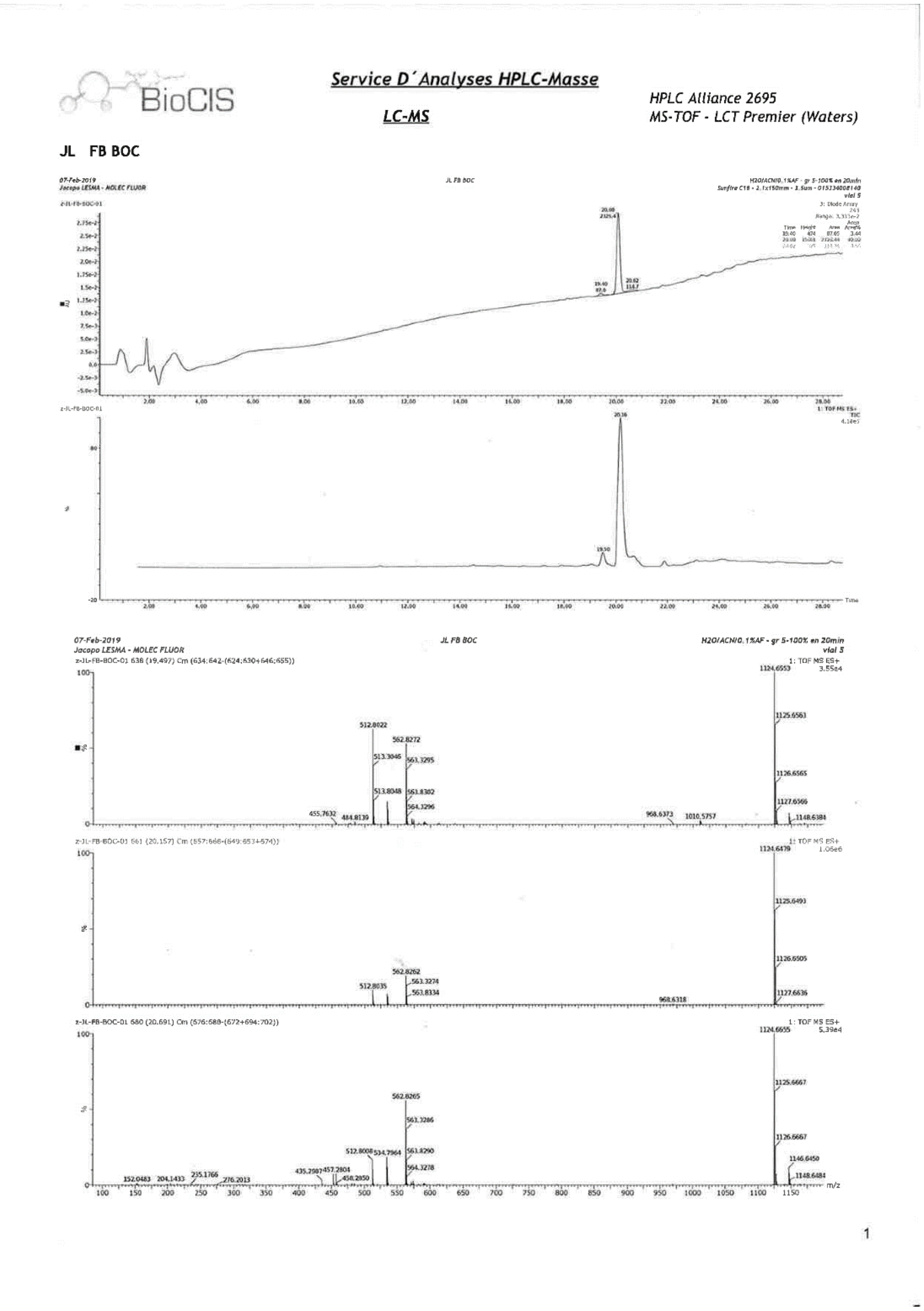

Figure S26 HPLC chromatogram and ESI-MS spectrum of compound **13**.

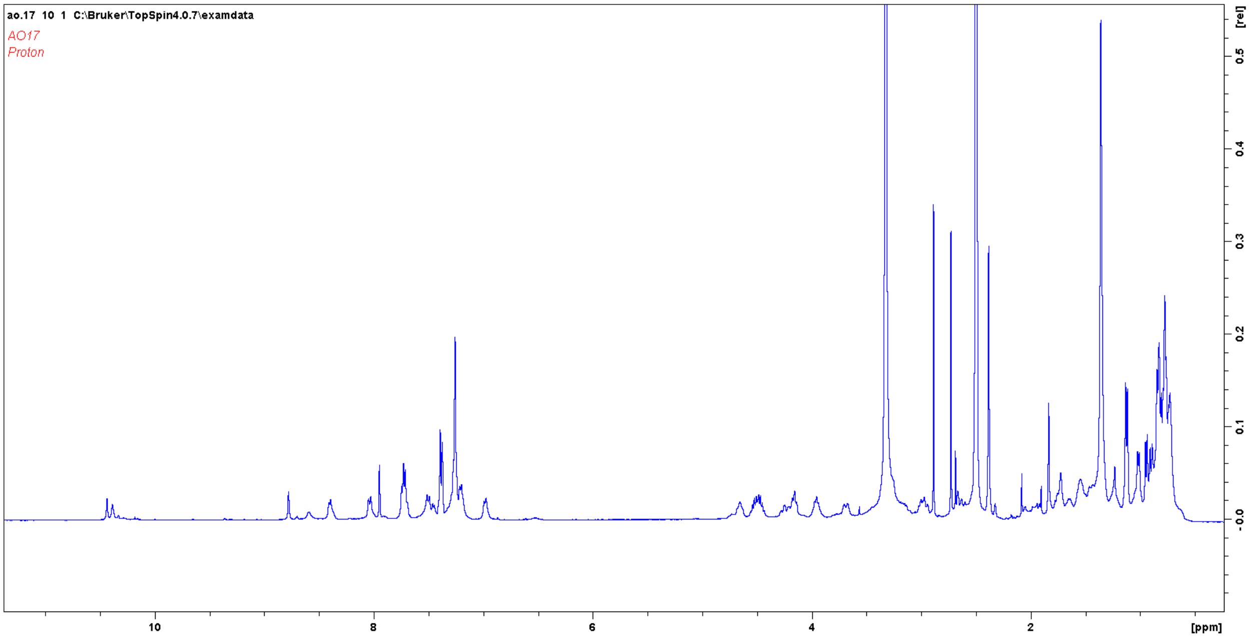

Figure S27 1D ^{1}H-NMR of compound **13** in CD_3OH at 278K

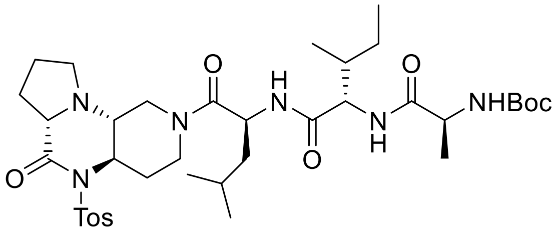

Figure S28 Structure of the cyclized by-product obtained from the side-reaction of cyclisation of compound **19**.

Compound [14]

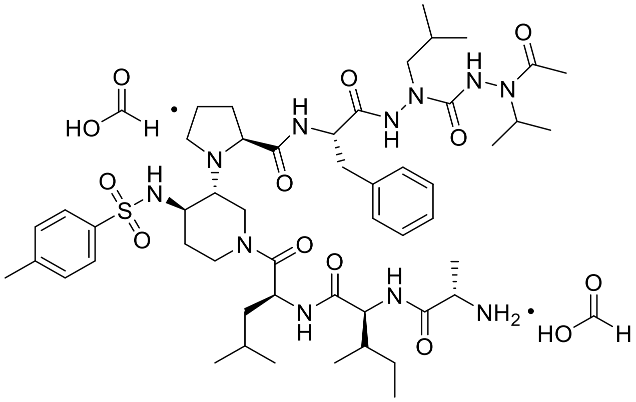

To a solution **13** (33 mg, 0.027 mmol, 1.0 eq.) in CH_2Cl_2 (1 mL) was added under argon atmosphere at 0°C HCl 4 M in dioxane (135 µL, 0.538 mmol, 20.0 eq.). The reaction mixture was stirred for 6 h at room temperature and to the suspension formed was added Et_2O. After decantation, the volatile was carefully removed with a Pasteur pipette. The solid obtained was purified by preparative HPLC eluting with H_2O + 0.1% formic acid/CH_3CN (gradient 20 to 80% in 15 min), on an Xselect column (4.6 x 150mm-5µm) to afford **14** (22 mg, 0.021 mmol, 27%) as a white powder.

Molecular weight = 1095.55 g mol^{-1}; free amine = 1023.59 g mol^{-1}
Rf = 0 (EtOAc)
HRMS: Calcd. for $[C_{51}H_{81}N_{11}O_9S+H]^+$: m/z 1024.6018, found: 1024.6018
HPLC purity: XBridge C18 3.5 µm; H_2O + 0.1 % form. ac./ACN, gradient 5–100 % in 20 min; R_t = 12.38 min, 100 %

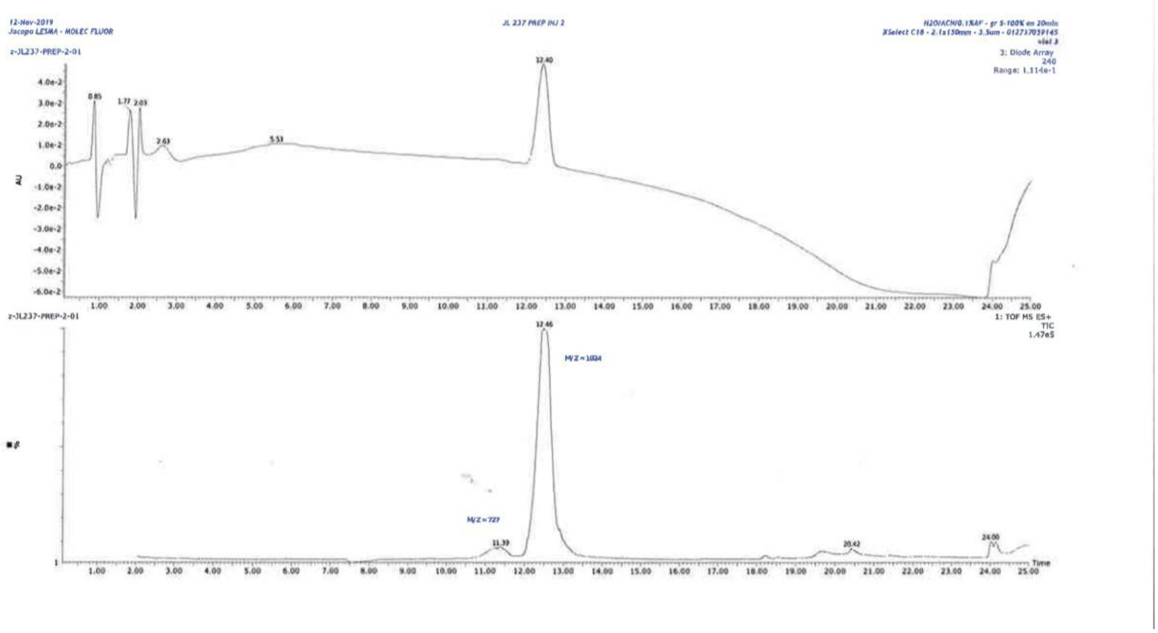

Figure S29 HPLC chromatogram and ESI-MS spectrum of compound **14**.

The ^{1}H and ^{13}C assignment of compound **14** was tricky even performed on a spectrometer operating at 800 MHz and equipped with a TCI cryoprobe. A dispersion of NH chemical shifts and poor resolution of the signals indicated the presence of different conformers whatever the temperature from 278K to 313K. Some signals and $^{3}J_{NH\text{-}H\alpha}$ coupling constants of the natural amino acids could not be determined with certainty (Table S15). The temperature dependence of amide proton chemical shifts of Ile-2 was intermediate (-4.5 ppb K^{-1}, Table S16), suggesting its partial involvement in hydrogen-bond. Several sequential $CH\alpha_i/NH_{i+1}$ ROEs and large and positive ^{1}H CSD values (Table S17, SI) for Ile, Leu and Phe confirmed the extended conformation of the natural amino acids in the two arms (Fig. S30). No typical spatial proximities were found between Pro-5 and Pip-4 and between the Pip-4 and Phe-6 to confirm the turn structure. However, both ROE between the Hα of Leu-3 and the diastereotopic protons H2 and H6 of the Pip-4 were visible, indicating an equilibrium between an "open" hairpin structure and a structure where the arms are closer (see discussion for conformers 9a and 9b). Numerous intra-strands ROEs were identified, in particular in the diaza-tripeptide arm (Fig. S31). A very weak inter-strand ROE was observed and attributed to Hδ PHE and Hδ LEU or Hγ ILEU which chemical shifts are identical (0,91 ppm, Fig. S31). Overall, **14** seems to adopt more flexible β-hairpin structure than the peptide analogues **8** and **9**.

Residue	δ NH (ppm)	δ H_α (ppm)	δ H_β (ppm)	δ other protons (ppm)	δ CO (ppm)	δ C_α (ppm)	δ C_β (ppm)	other carbons (ppm)
**Ala-1**	8.22<br>8.25<br>8.28	3.96	1.45	/	169.6	48.7	16.5	
**Ile-2**	8.44	4.22	1.79	γ_{CH3}0.91<br>γ_{CH2} 1.17,1.55<br>δ_{CH3} 0.89	171.9	57.85	36.5	γ_{CH3} 14.4<br>γ_{CH2} 24.4<br>δ_{CH3} 9.9
**Leu-3**	8.55	4.76	1.61	γ_{CH} 1.29<br>δ_{CH3} 0.91	/	47.3	40.0	γ_{CH} 40.2<br>δ_{CH3} 14.5
**Pip-4**	/	/	/	$H_{2/2'}$ 4.30/2.53<br>H_3 3.42<br>H_4 3.63<br>$H_{5/5'}$ 1.32/1.50<br>$H_{6/6'}$ 3.82/3.15	/	/	/	$C_{2/2'}$ 40.4<br>H_3 63.3<br>H_4 51.1<br>$H_{5/5'}$ 29.91<br>$H_{6/6'}$ 43.31
**Pro-5**	/	4.40	2.70/2.18	γ 1.94<br>δ 3.20/3.08	/	54.7	30.5	γ 26.95<br>δ 48.0
**Phe-6**	/	4.56	3.21	Ho 7.35<br>Hm 7.31<br>Hp 7.28	/	48	36.6	Cq 135.4<br>Co 128.5<br>Cm 129.2<br>Cp 127.0
**Aza-Leu-7**	10.65	/	2.48,<br>3.37	γ_{CH} 1.13<br>δ_{CH3} 0.72	/	/	55.1	γ_{CH} 24.4<br>δ_{CH3} 19.6

**Aza-Val-8**	/		4,65	δ 1.05	174.1		48.1	18.2
**Tosyl**	7.88	/	/	H_o 7.83,<br>H_m 7.44<br>CH_3 2.44	/	/	/	Cqs 144.0<br>Cqc 137.6<br>Co 126.8<br>Cm 129.7<br>CH_3 20.0
**CH_3 (Ac)**	/	/	/	CH_3 1.99	169.6	/	/	CH_3 20.1

Table S15 ^{1}H-NMR and ^{13}C-NMR chemical shifts for the mean conformer of hairpin **14** (10 mM) in CD_3OH at 278K

Residue	δ HN (ppm 278K)	δ HN (ppm 313K)	Δδ/ΔT (ppb K^{-1})
**Ile-2 NH**	8.44	8.28	-4.5
**Leu-3 NH**	8.55	8.28	-7.7

Table S16 Temperature coefficients for the NH of the major conformer of **14** in CD_3OH between 278K and 313K

Residue	δ NH random coil	δ NH experimental	NH CSD	δ H^{α} random coil	δ H^{α} experimental	H^{α} CSD
**Ala-1**	8.24	8.22/8.25/8.28	/	4.32	3.96	-0.36
**Ile-2**	8.00	8.44	+ 0.44	4.17	4.22	+ 0.05
**Leu-3**	8.16	8.55	+ 0.39	4.34	4.76	+ 0.42
**Pro-5**	/	/	/	4.42	4.40	-0.02
**Phe-6**	8.30	8.81	+0.51	4.62	4.56	- 0.06
**Residue**	**δ C^{α} random coil**	**δ C^{α} experimental**	**C^{α} CSD**	**δ C=O random coil**	**δ C=O experimental**	**C=O CSD**
**Ala-1**	52.5	48.7	-3.8	177.8	169.6	-8.2
**Ile-2**	61.1	57.8	-3.3	176.4	171.9	-4.5
**Leu-3**	55.1	47.3	-7.8	177.6		
**Pro-5**	63.3	54.7	-8.6	177.3	/	/
**Phe-6**	57.7	48	-9.7	175.8	/	/

Table S17 Chemical shift deviations (CSD) of HN, H^{α}, C^{α} and CO, for compound **14**

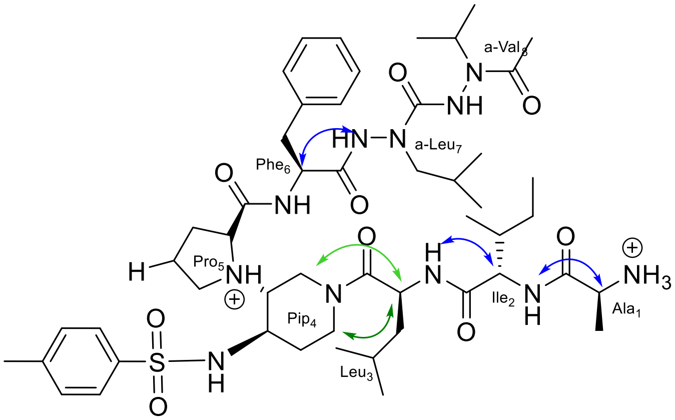

Figure S30 Structure of hairpin **14** showing the assigned ROEs: in blue the sequential CHα$_i$/NH$_{i+1}$ ROEs, and in green other significant ROEs (light green/weak ROE; dark green/ strong ROE).

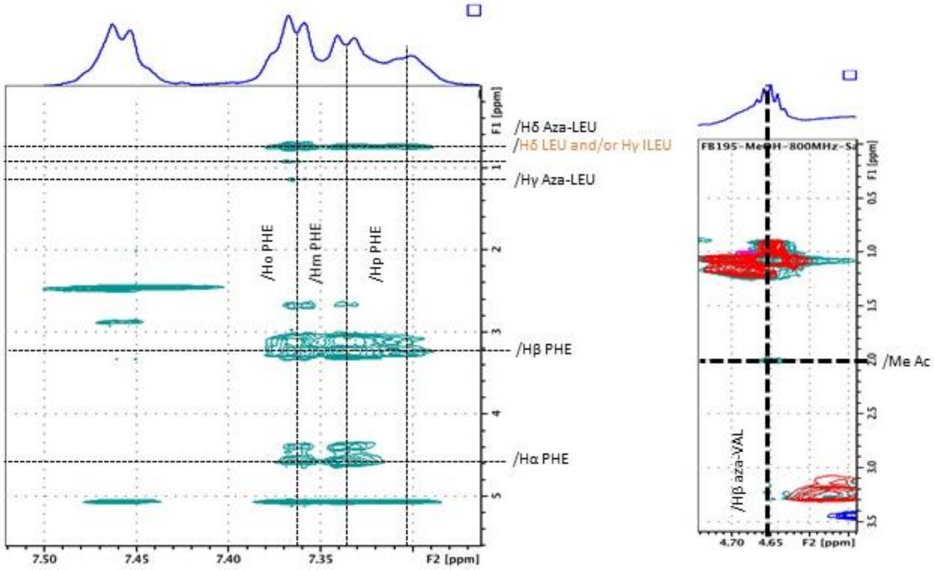

Figure S31 Expansion of the 2D ^{1}H-^{1}H ROESY of compound **14** showing the intra-strand ROEs involving the diaza-tripeptide arm and the weak inter-strand ROE (in light orange)

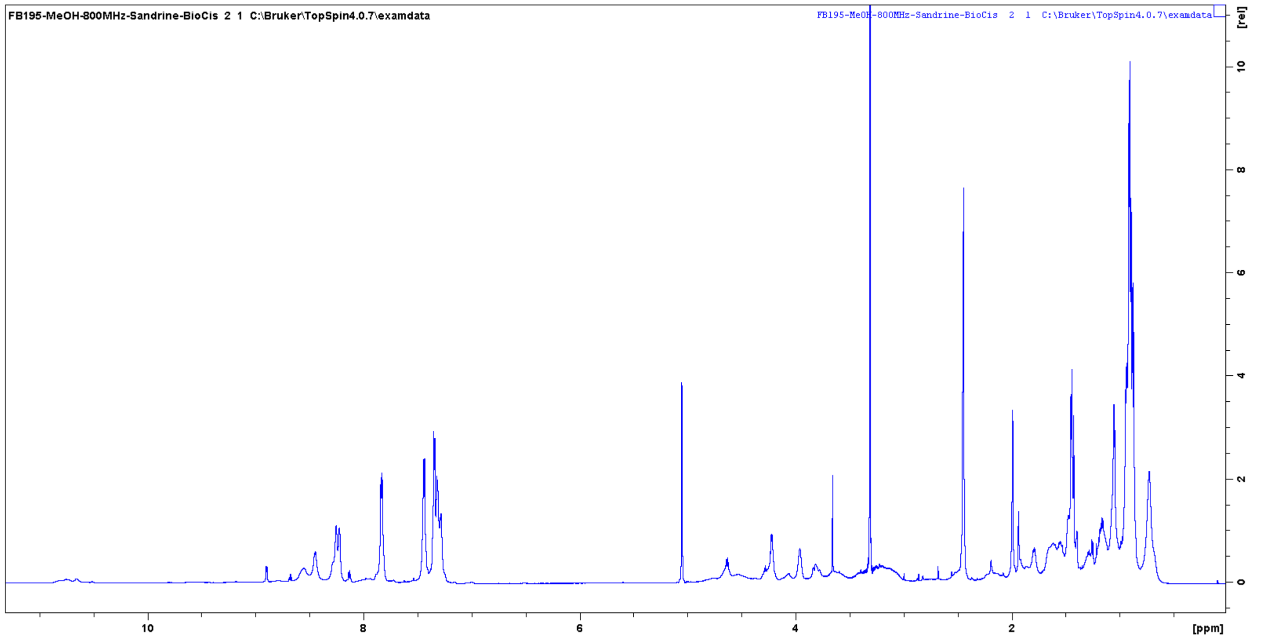

Figure S32 1D ^{1}H-NMR of compound **14** in CD_3OH at 278K

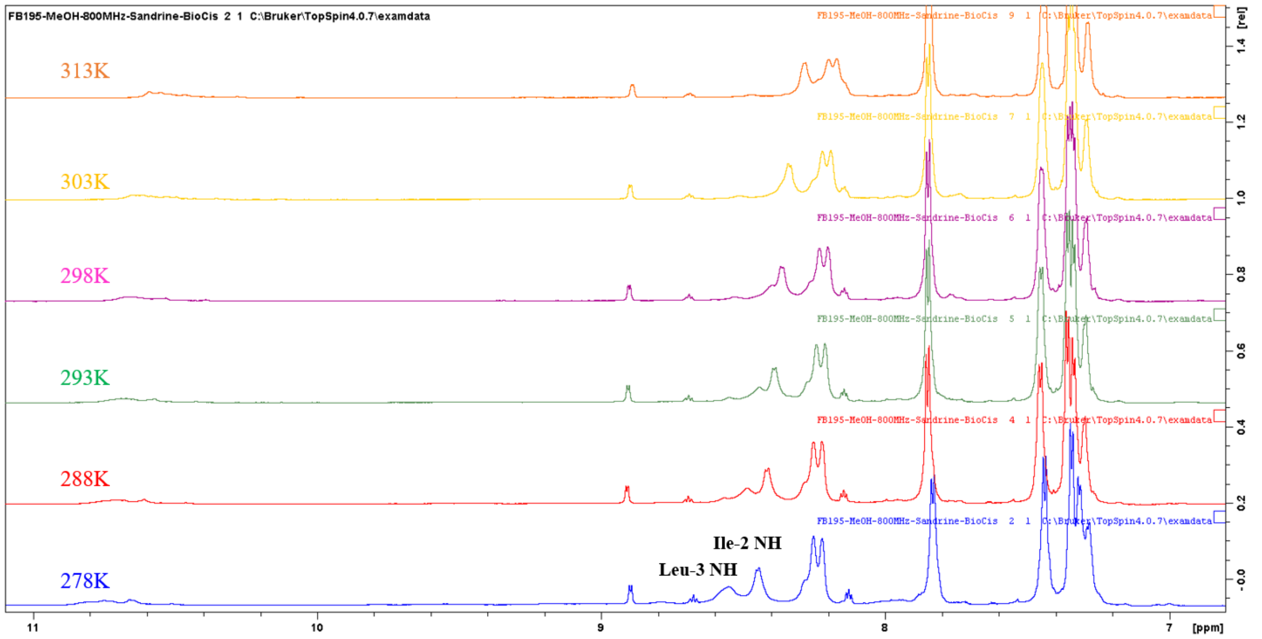

Figure S33 ^{1}H-NMR amide region spectra at different temperatures from 278K to 313K in CD_3OH for compound **14**

Synthesis and characterization of the intermediates:

Methyl((3*R*,4*R*)-1-((*tert*-butoxycarbonyl)-*L*-alanyl-*L*-isoleucyl-*L*-leucyl)-4-((4-methylphenyl)sulfonamido)piperidin-3-yl)-*L*-prolinate [11]

To a stirred solution of **18** (590 mg, 0.72 mmol, 1 eq.) in dry DMF (5 mL) piperidine (2 mL, 20%/vol. DMF) was added under argon atmosphere. The reaction was let under stirring 2 h and then the volatiles were removed under reduced pressure. The crude free amine obtained was kept aside without further purification before peptic coupling. To a solution of *L*-Boc-NH-Ala-OH (272 mg, 1.44 mmol, 2 eq.) in dry DMF (4 mL) were added successively HBTU (547 mg, 1.44 mmol, 2 eq.) and HOBt (196 mg, 1.44 mmol, 2 eq.) at 0°C. At this time, a solution of the previous free amine (438 mg, 0.72 mmol, 1 eq.) and DIPEA (0.50 mL, 2.88 mmol, 4 eq.) in DMF (4 mL) was added dropwise at 0°C on the previous one and the reaction was let stirring overnight. After removal of the solvent under reduced pressure, the crude oil obtained was taken up with EtOAc and successively washed with distilled water, 100% aqueous solution of $NaHCO_3$ and brine. The organic phase was dried over Na_2SO_4, filtered and the volatile was then removed under reduced pressure. The crude residue obtained was purified by column chromatography with silica gel eluting with c-Hex/EtOAc 4:6 to afford **11** as a white powder (414 mg, 0.53 mmol, 74%).

Molecular weight = 779.01 g mol^{-1}
Rf = 0.43 (c-Hex/EtOAc 4:6)
HRMS: Calcd. for [$C_{38}H_{62}N_6O_9S$ + H]$^+$: m/z 779.4361, found: 779.4364 Calcd. for [$C_{38}H_{62}N_6O_9S$ + Na]$^+$: M/z 801.4182, found: 801.4188
^{1}H NMR ($CDCl_3$, 400 MHz): δ 7.79 (2H, d, *J* = 7.9 Hz); 7.30 (2H, d, *J* = 7.8 Hz); 7.32 (1H, bs); 6.72 (1H, bs); 6.69 (1H, bs); 5.05 (1H, bs); 4.92 (1H, m); 4.47 (1H, m); 4.28 (1H, m); 4.16 (1H, m); 3.76 (3H, s); 3.81 (1H, m); 3.55 (1H, m); 3.54 (1H, m); 2.97 (1H, m); 2.85 (1H, m); 2.56 (1H, m); 2.46 (1H, m); 2.42 (3H, s); 2.31 (1H, m); 2.28 (1H, m); 2.05 (1H, m), 1.88 (1H, m); 1.86 (1H, m); 1.62 (1H, m); 1.55 (2H, m); 1.51 (3H, m); 1.42 (9H, s); 1.41 (1H, m), 1.32 (3H, d, *J* = 6.7 Hz); 1.09 (1H, m); 0.93 (3H, m); 0.88 (3H, m); 0.86 (3H, m); 0.84 (3H, m) ppm
^{13}C NMR ($CDCl_3$, 100 MHz): δ 175.5; 172.6; 172.3; 170.4; 143.4; 137.2; 129.7; 127.2; 80.7; 58.0; 53.5; 52.8; 52.6; 50.5; 47.5; 44.0; 43.7; 43.1; 42.8; 40.7; 37.2; 33.4; 30.0; 28.4; 24.8; 24.6; 21.7; 17.6; 15.6; 11.5 ppm
Melting point = 118–120°C
IR: 3284; 2961; 1721, 1632; 1519, 1447; 1213; 1162 cm^{-1}

(*S*)-1-(((*S*)-1-(((*S*)-1-amino-3-methyl-1-oxobutan-2-yl)amino)-4-methyl-1-oxopentan-2-yl)amino)-1-oxo-3-phenylpropan-2-aminium chloride [12]

To a stirred solution of Boc-NH-Phe-Leu-Val-$CONH_2$ (200 mg, 0.42 mmol, 1.0 eq.) in dioxane (10 mL) under argon atmosphere, at 0°C, HCl 4M in dioxane (6.3 mL, 25.2 mmol, 60.0 eq.) was added. The reaction was let stirring 4 h at room temperature. After removal of the volatile under

reduced pressure, the hydrochloride salt **12** was yielded as a white solid (174 mg, 0.42 mmol, quant.)

Molecular weight = 412.22 g mol^{-1}; free amine 376.50 g mol^{-1}
Rf = 0 (c-Hex/EtOAc 1:1)
HRMS: Calcd. for $[C_{20}H_{32}N_4O_3 + H]^+$: m/z 377.2508, found: 377.2545
^{1}H NMR (DMSO-*d6*, 400 MHz): δ 8.80 (1H, d, *J* = 8.4 Hz); 8.21 (2H, bs); 7.99 (1H, bs); 7.44 (1H, bs); 7.32 (5H, bs); 7.07 (1H, bs); 4.48 (1H, m), 4.19-4.09 (2H, m); 3.20 (1H, dd, *J* = 14.1, 5.4 Hz); 2.98 (1H, dd, *J* = 14.1, 7.7 Hz); 2.00 (1H, m); 1.69 (1H, m); 1.52 (2H, m), 0.95-0.88 (12H, m) ppm
^{13}C NMR (DMSO-*d6*, 100 MHz): δ 173.6; 172.1; 168.5; 135.7; 130.6,129.3,128.0; 58.3; 53.9; 52.3; 41.9; 37.6; 31.4; 25.0; 24.0, 22.6; 20.2, 19.0 ppm
Melting point = 80-83°C
IR: 3272; 2954; 1675-1637; 1547 cm^{-1}

N'-(*L*-phenylalanyl)-*N*'-acetyl-*N*-isobutyl-*N*'-isopropylmethanedihydrazide [15]

To a solution of **25** (94 mg, 0.184 mmol, 1.0 eq.) in CH_2Cl_2 and MeOH under argon atmosphere, Pd/C (19 mg, 20 % mass) and triethyl silane (294 µL, 1.84 mmol, 10 eq.) were added. The reaction mixture was stirred for 20 minutes at room temperature and filtered on a Celite pad. After concentration of the filtrate under reduced pressure, **15** (70 mg, 0.184 mmol, quant.) was afforded as a white solid.

Molecular weight = 377.48 g mol^{-1}
Rf = 0 (c-Hex/EtOAc 3:7)
HRMS: Calcd. for $[C_{19}H_{31}N_5O_3 + H]^+$: m/z 378.2505, found: 378.2501
^{1}H NMR (CD_3OD, 400 MHz): δ 7.23-7.31 (5H, m); 4.63 (1H, m); 3.59 (1H, m); 3.28 (1H, m), 3.41 (1H, m); 2.98 (2H, m); 1.96 (3H, s); 1.23 (1H, m); 1.07 (6H, m); 0.73 (6H, m) ppm
^{13}C NMR (CD_3OD, 100 MHz): δ 176.0; 175.1; 158.9; 130.1; 129.6; 127.7; 138.2; 56.4; 56.1; 48.8; 42.6; 26.8; 21.0; 19.64; 19.2 ppm

Melting point = 244-246°C
IR: 3318; 2928; 1691; 1495;1351; 1213; 1167 cm^{-1}

S)-Methyl-1-((3R,4R)-4-(4-methylphenylsulfonamido)piperidin-3-yl)pyrrolidine-2-carboxylate [16]

To a solution of compound piperidine pyrrolidine β-turn inducer (1 g, 2.12 mmol, 1 eq.) in toluene (30 mL) was added Pd/C 10% (0.45 g, 4.24 mmol, 2 eq.). The mixture was kept under stirring at 30 °C overnight under hydrogen atmosphere. After filtration through a pad of Celite with MeOH,

the solvent was evaporated under reduced pressure to yield a colorless oil with a quantitative yield (0.81 g, 2.12 mmol).

Molecular weight = 381.17 g mol-1
Rf = 0.20 (DCM/MeOH 20:1)

HRMS: Calcd. for $[C_{18}H_{27}N_3O_4S + H]^+$: M/z 382.1801, found: 382.1800
^{1}H NMR (CDCl3, 300 MHz, 298 K): δ 7.76 (2H, d, J = 7.6 Hz); 7.26 (2H, d, J= 7.6 Hz); 6.79 (1H, bs); 3.74 (3H, s); 3.45 (2H, m); 2.97 (2H, m); 2.74 (2H, m); 2.49 (6H, m); 2.24 (1H, m); 1.99 (1H, m); 1.77 (2H, m); 1.39 (3H, m) ppm
^{13}C NMR (CDCl3, 75 MHz, 298 K): *δ* 176.3; 143.0; 37.5; 129.9; 127.4; 62.3; 59.9; 54.2; 52.6; 45.1; 44.9; 44.3; 35.3; 30.0; 24.6; 21.7 ppm
IR: 3206; 2951, 2849; 1727; 1669; 1438; 1209, 1161 cm^{-1}

Methyl((3*R*,4*R*)-1-((((9H-fluoren-9-yl)methoxy)carbonyl)-*L*-leucyl)-4-((4-methylphenyl)sulfonamido)piperidin-3-yl)-*L*-prolinate [17]

To a solution of *L*-Fmoc-NH-Leu-OH (757 mg, 2.14 mmol, 2 eq.) in dry DMF under argon atmosphere HATU (814 mg, 2.14 mmol, 2 eq.) and HOAt (292 mg, 2.14 mmol, 2 eq.) were added at 0°C. The mixture was let 40 min at 0°C under stirring. Meanwhile a solution of compound **16** (408 mg, 1.07 mmol, 1 eq.) and collidine (0.85 mL, 6.43 mmol, 6 eq.) in dry DMF was prepared and added dropwise at 0°C on the previous mixture. The reaction was let stirring overnight at room temperature. After removal of the volatile under reduced pressure, the crude oil was taken up with EtOAc and washed with distilled water, 100% aqueous solution of $NaHCO_3$ and brine, dried over $MgSO_4$, filtered and concentrated under reduced pressure. The crude residue obtained was purified by column chromatography on silica gel eluting with c-Hex/EtOAc 6:4 to afford **17** as a white powder (645 mg, 0.9 mmol, 84%).

Molecular weight = 716.32 g mol^{-1}
Rf = 0.4 (c-Hex /EtOAc 6:4)
HRMS: Calcd. for $[C_{39}H_{48}N_4O_7S + H]^+$: m/z 717.3322, found: 717.3317
^{1}H NMR ($CDCl_3$, 400 MHz): *δ* 7.84-7.72 (4H, m); 7.56 (2H, m); 7.39 (2H, m); 7.31 (2H, m); 7.28 (2H, m); 7.01 (1H, bs); 5.46 (1H, bs); 4.65 (1H, m); 4.34 (2H, m); 4.19 (1H, m); 4.13 (2H, m); 3.78 (3H, s), 3.76 (1H, m); 3.60 (1H, m); 3.05 (2H, m); 2.95 (1H, m); 2.48 (3H, s); 2.47 (2H, m); 2.43 (2H, m); 2.09 (1H, m); 1.92 (1H, m); 1.64 (1H, m); 1.53 (2H, m); 1.49 (2H, m); 0.96 (3H, d, *J* = 6.2 Hz); 0.86 (3H, d, *J* = 6.5 Hz) ppm
^{13}C NMR ($CDCl_3$, 100 MHz): *δ* 175.8; 171.2; 156.4; 143.9; 143.3; 141.4; 129.6; 127.7; 127.2; 127.1; 125.1; 119.9; 67.0; 60.4; 52.8; 52,6; 52.5; 49.4; 47.2; 44.0; 42.6; 42.4; 41.2; 41.0; 29.8; 24.6; 21.8; 21.5 ppm
Melting point = 90-92°C
IR: 3201; 2954; 1721, 1635; 1448; 1212; 1162 cm^{-1}

Methyl((3*R*,4*R*)-1-((((9H-fluoren-9-yl)methoxy)carbonyl)-*L*-isoleucyl-*L*-leucyl)-4-((4-methylphenyl)sulfonamido)piperidin-3-yl)-*L*-prolinate [18]

To a stirred solution of **17** (640 mg, 0.894 mmol, 1 eq.) in dry DMF (10 mL) piperidine (2 mL, 20% vol in DMF) was added. The reaction was let under stirring 2 h and then the volatile were removed under reduced pressure. The crude free amine was kept aside without further purification before peptidic coupling. Meanwhile, to a solution of *L*-Fmoc-NH-Ile-OH (632 mg, 1.79 mmol, 2 eq.) in dry DMF at 0°C HBTU (678 mg, 1.79 mmol, 2 eq.) and HOBt (243 mg, 1.79 mmol, 2 eq.) were added. At this time, a solution of the previous free amine (442 mg, 0.894 mmol, 1 eq.) in dry DMF and DIPEA (0.63 mL, 3.58 mmol, 4 eq.) was added at 0°C. The reaction was stirred overnight under argon atmosphere at room temperature. After concentration under reduced pressure, the remaining crude oil was taken up with EtOAc, and washed successively with distilled water, 100% aqueous solution of $NaHCO_3$ and brine. The organic layer was dried over Na_2SO_4, filtered and the volatile was removed under reduced pressure. The crude residue obtained was purified by column chromatography on silica gel eluting with c-Hex/EtOAc 6:4 to afford **18** as a white powder. (590 mg, 0.702 mmol, 79%).

Molecular weight = 829.41 g mol^{-1}
Rf = 0.2 (c-Hex/EtOAc 6:4)
HRMS: Calcd. for $[C_{45}H_{59}N_5O_8S + H]^+$: m/z 830.4163, found: 830.4171
^{1}H NMR ($CDCl_3$, 300 MHz): δ 7.84-7.81 (4H, m); 7.60 (2H, m); 7.39 (2H, m); 7.34-7.24 (4H, m); 6.99 (1H, bs); 6.47 (1H, bs); 5.38 (1H, bs); 4.93 (1H, m); 4.65 (1H, m); 4.43 (2H, m); 4.25 (2H, m); 4.06 (1H, m); 3.78 (1H, m); 3.58 (3H, s); 3.55 (1H, m); 3.10-2.80 (3H, m); 2.42 (2H, m); 2.60-2.35 (6H, m); 1.83 (2H, m); 1.54 (4H, m); 1.43 (3H, m); 0.97 (3H, s); 0.89 (9H, m) ppm
^{13}C NMR ($CDCl_3$, 300 MHz): δ 170.8; 165.4; 161.4; 156.3; 141.4; 141.3; 129.7; 127.8; 127.4; 127.2; 125.2; 120.1; 67.2; 61.5; 59.9; 53.4; 52.7; 52.6; 48.3; 47.4; 42.9; 41.2; 40.7; 37.8; 25.1; 25.0; 24.6; 21.6; 15.6; 14.3; 11.7 ppm
Melting point = 104–106°C
IR: 3285; 2959; 1722, 1629; 1523, 1449; 1213; 1163 cm^{-1}

((3R,4R)-1-((tert-butoxycarbonyl)-L-alanyl-L-isoleucyl-L-leucyl)-4-((4-methylphenyl)sulfonamido)piperidin-3-yl)-L-proline [19]

To a solution of **11** (260 mg, 0.33 mmol, 1 eq.) in MeOH (10 mL) NaOH 2M (0.84 mL, 1.65 mmol, 5 eq.) was added. After stirring for 3 h at 60°C, the volatile was removed under reduced pressure and the obtained oil was dissolved in distilled water, acidified until pH 4 with 10% aqueous solution of $KHSO_4$. The precipitate which was formed at this time was dissolved in CH_2Cl_2. The organic layer was separated and let aside. Meanwhile, the aqueous phase was extracted several times with CH_2Cl_2. The combined organic phase was dried over Na_2SO_4, filtered

and the volatile was removed under vacuum to afford **19** as a white powder (239 mg, 0.31 mmol, 94%).

Molecular weight = 764.97 g mol^{-1}
Rf = 0.3 (EtOAc/MeOH 9:1)
HRMS: Calcd. for $[C_{37}H_{60}N_6O_9S + H]^+$: m/z 765.4221, found: 765.4222; Calcd. for $[C_{37}H_{60}N_6O_9S + Na]^+$: M/z 787.4048, found: 787.4045
^{1}H NMR (DMSO-*d6*, 400 MHz): δ 12.55 (1H, bs); 8.02 (1H, bs); 7.70 (2H, m); 7.52 (1H, bs); 7.41 (2H, m); 7.00 (1H, bs); 4.70 (1H, m); 4.18 (1H, m); 4.17 (1H, m); 3.97 (1H, m); 3.72 (1H, m); 3.41 (1H, m); 3.06 (1H, m); 3.00 (1H, m); 2.73 (1H, m); 2.39 (3H, s); 2.41 (1H, m); 2.26 (1H, m); 2.15 (1H, m); 2.01 (1H, m); 1.85 (1H, m); 1.77 (1H, m); 1.66 (1H, m); 1.56 (1H, m); 1.50 (1H, m); 1.46 (1H, m); 1.37 (9H, s); 1.35 (1H, m); 1.34 (1H, m); 1.29 (1H, m); 1.28 (1H, m); 1.13 (3H, d, *J* = 6.9 Hz); 1.03 (1H, m); 0.86 (3H, m); 0.83 (3H, m); 0.78 (3H, m); 0.77 (3H, m) ppm
^{13}C NMR (DMSO-*d6*, 100 MHz): δ 176.1; 171.8; 169.9; 169.3; 154.3: 142.3; 136.5; 129.7; 126.4; 77.4; 61.4; 58.0; 56.0; 52.2; 49.5; 46.0; 44.1; 43.2; 40.2; 39.5; 36.6; 32.3; 29.1; 27.7; 23.9; 23.7; 23.3; 22.6; 21.6; 20.6; 17.4; 14.9; 10.6 ppm
Melting point = 144-148°C
IR: 3292; 2962; 1636; 1521, 1450; 1225;1160 cm^{-1}

***Tert*-butyl 2-isopropylhydrazine-1-carboxylate [20]**

To a stirred solution of *tert*-butylcarbazate (2 g, 15.1 mmol, 1 eq.) in dry THF (20 mL) under argon atmosphere, acetone (3.3 mL, 45.3 mmol, 3 eq.) and acetic acid (1.05 mL, 13.6 mmol, 0.9 eq.) were added. After 3 h, the volatiles were removed under reduced pressure and the remaining oil was taken up with dry THF (100 mL). To this solution, under argon atmosphere, $NaBH_3CN$ (1.56 g, 22.7 mmol, 1.5 eq.) was added with some seeds of bromocresol green. At that time, a solution of 4-toluene-sulfonic acid (2.86 g, 16.6 mmol, 1.1 eq.) in dry THF (4 mL) was added dropwise and the mixture became yellow. After 1 h the volatiles were removed under reduced pressure. The remaining powder was dissolved in a mixture of EtOAc and brine and then taken up again with CH_2Cl_2. The organic layer was washed with a mixture of 10% aqueous solution of NaCl/$NaHCO_3$ 1/1, dried over Na_2SO_4, filtered, and concentrated under reduced pressure. The crude material obtained was then dissolved in MeOH (10 mL) and NaOH 1 M (18 mL, 18.1 mmol, 1.2 eq.) was added. After stirring for 1 h at room temperature, volatile was removed under reduced pressure and the crude oil obtained was taken up with EtOAc, washed with brine, dried over Na_2SO_4 and filtered. After concentration under reduced pressure, the residue obtained was purified by column chromatography on silica gel eluting with CH_2Cl_2/MeOH 98:2 to yield **20** (1.37 g, 7.86 mmol, 52%) as an oil that crystallized in the fridge.

Molecular weight = 174.24 g mol^{-1}
Rf = 0.5 (CH_2Cl_2/MeOH 98:2)

HRMS: Calcd. for [$C_8H_{18}N_2O_2$ + Na]$^+$: m/z 197.1266, found: 197.1266
^{1}H NMR ($CDCl_3$, 300 MHz): δ 5.31 (2H, bs); 3.28 (1H, m); 1.47 (9H, s); 1.12 (6H, d, J = 6.4 Hz) ppm
^{13}C NMR ($CDCl_3$, 75 MHz): δ 156.9; 80.3; 50.8; 28.3; 20.6 ppm
Melting point = 177-179°C
IR: 3197; 2988, 2938, 2901; 1737; 1340, 1320; 1199, 1149 cm^{-1}

***Tert*-butyl 2-acetyl-2-isopropylhydrazine-1-carboxylate [21]**

To a solution of **20** (1.37 g, 7.86 mmol, 1 eq.) in dry THF was added under argon atmosphere acetic anhydride (4.4 mL, 47.16 mmol, 6 eq.). The reaction was stirred overnight at 60°C. After removal of the volatile under reduced pressure, the crude residue was purified by column chromatography on silica gel eluting with CH_2Cl_2/MeOH 98:2 to yield **21** (1.63 g, 7.53 mmol, 96%) as a white powder.
Molecular weight = 216.28 g mol^{-1}
Rf = 0.4 (CH_2Cl_2/MeOH 98:2)

HRMS: Calcd. for [$C_{10}H_{20}N_2O_3$ + H]$^+$: m/z 217.1552, found: 217.1549; Calcd. for [$C_{10}H_{20}N_2O_3$ + Na]$^+$: M/z 239.1372, found: 239.1374
^{1}H NMR ($CDCl_3$, 400 MHz): δ 6.47 (1H**,** bs); 4.80 (1H, m); 2.05 (3H, s); 1.48 (9H, s); 1.09 (6H, d, J = 6.8 Hz) ppm
^{13}C NMR ($CDCl_3$, 100 MHz): δ 173.4; 155.2; 82.2; 47.6; 28.7; 21.6; 19.8 ppm
Melting point = 79-81°C
IR: 3255; 2979; 1738; 1411;1367; 1242; 1158 cm^{-1}

***N*-isopropylacetohydrazide hydrochloride [22]**

To a solution of **21** (0.58 g, 2.69 mmol, 1 eq.) in dioxane under argon atmosphere, HCl 4 M in dioxane (20 mL, 80.6 mmol, 30 eq) was added at 0°C. After stirring overnight at room temperature, a precipitate appeared. The solvent was removed with a Pasteur pipette, and the white solid obtained was washed carefully with diethyl ether twice, dried under reduced pressure to afford the hydrochloride salt **22** (0.411 g, 2.69 mmol, quant.) as a white powder.

Molecular weight = 152.62 g mol^{-1}; free amine = 116.16 g mol^{-1}
Rf = 0 (CH_2Cl_2/MeOH 98/2)

HRMS: Calcd. for [$C_5H_{12}N_2O$ + H]$^+$: m/z 117.1028, found: 117.1023

^{1}H NMR (CD_3OD, 400 MHz): δ 4.51 (1H, m); 2.24 (3H, s); 1.33 (6H, d, J = 6.8 Hz)

^{13}C NMR (CD_3OD, 100 MHz): δ 52.3; 20.3; 19.8 ppm
Melting point = 174-176°C
IR: 3359; 2993; 1657; 1371, 1157 cm^{-1}

***Tert*-butyl 2-(2-acetyl-2-isopropylhydrazine-1-carbonyl)-2-isobutylhydrazine-1-carboxylate [23]**

To a stirred solution of **22** (142 mg, 0.93 mmol, 1.2 eq.) in dry CH_2Cl_2 under argon atmosphere at 0°C, pyridine (0.34 mL, 4.22 mmol, 6 eq.) was added. After a period 10 min, 4-nitrophenyl chloroformate (184 mg, 0.92 mmol, 1.3 eq.) in dry CH_2Cl_2 (0.5 mL) was added dropwise. The reaction was stirred overnight at room temperature and the volatile were then removed under reduced pressure. To the crude residue, dissolved in dry DMF (2 mL), a solution of azaLeu (132 mg, 0.71 mmol, 1.0 eq.) in dry DMF (0.7 mL) and DMAP (129.2 mg, 1.05 mmol, 1.5 eq.) were added under argon atmosphere. After stirring overnight at 40°C and concentration under reduced pressure, the crude residue afforded was purified by column chromatography on silica gel eluting with EtOAc to yield **23** (170 mg, 0.52 mmol, 74%) as a white powder.

Molecular weight = 330.42 g mol^{-1}

R*f* = 0.4 (EtOAc)
HRMS: Calcd. for $[C_{15}H_{30}N_4O_4 + Na]^+$: M/z 353.2165, found: 353.2174
^{1}H NMR ($CDCl_3$, 400 MHz): δ 7.47 (1H, bs); 6.94 (1H, bs); 4.72 (1H, m); 2.09 (3H, s); 1.80 (1H, m); 1.43 (9H, s); 1.01 (6H, m); 0.84 (6H, m) ppm
^{13}C NMR ($CDCl_3$, 100 MHz): δ 173.2; 169.7; 156.7; 82.7; 55.8; 47.0; 27.9; 26.2; 21.0; 19.7; 18.8 ppm
Melting point = 79-81°C

***N*'-Acetyl-*N*-isobutyl-*N*'-ispropylmethanedihydrazide hydrochloride [24]**

To a solution **23** (305 mg, 0.93 mmol, 1 eq.) in dioxane under argon atmosphere HCl 4 M in dioxane (9.3 mL, 37.2 mmol, 40 eq) was added at 0°C. The mixture was stirred 3 h at room temperature. After removal of the volatile under reduced pressure, the hydrochloride salt **24** (248 mg, 0.93 mmol, quant.) was afforded as a white powder.

Molecular weight = 266.77 g mol^{-1}; free amine = 230:31 g mol^{-1}
Rf = 0 (EtOAc)
HRMS: Calcd. for $[C_{10}H_{22}N_4O_2 + H]^+$: m/z 231.1821, found: 231.1818; Calcd. for $[C_{10}H_{22}N_4O_2 + Na]^+$: m/z 253.1640, found: 253.1631
^{1}H NMR (CD_3OD, 400 MHz): δ 4.74 (1H, m); 3.70 (2H, m); 2.16 (3H, s); 2.06 (1H, m); 1.17 (6H, m); 1.00 (3H, m) 0.92 (3H, m) ppm
^{13}C NMR (CD_3OD, 100 MHz): δ 173.3, 170.2; 54.7; 49.0; 27.4; 20.7; 19.8; 19.5 ppm
Melting point = 72-74°C
IR: 3186; 2962; 1685; 1529;1468; 1282 cm^{-1}

Benzyl (*S*)-(1-(2-(2-acetyl-2-isopropylhydrazine-1-carbonyl)-2-isobutylhydrazinyl)-1-oxo-3-phenylpropan-2-yl)carbamate [25]

To a solution of *L*-Cbz-NH-Phe-OH (154 mg, 0.514 mmol, 1 eq.) in dry DMF, HBTU (399 mg, 1.03 mmol, 2 eq.) and HOBt (139 mg, 1.03 mmol, 2 eq.) were successively added at 0°C. The reaction mixture was stirred for 40 min at 0°C under argon atmosphere. At this moment, a solution of compound **24** (137 mg, 0.514 mmol, 1 eq.) and DIPEA (0.54 mL, 3.08 mmol, 6 eq.) in dry DMF was added dropwise. The reaction was let stirring for 2 days at room temperature under argon atmosphere. The volatile was removed under reduced pressure and the crude oil taken up with EtOAc, washed with distilled water, 100% aqueous solution of $NaHCO_3$ and brine, dried over Na_2SO_4, filtered and concentrated under vacuum. The crude residue obtained was purified by column chromatography on silica gel eluting with c-Hex/EtOAc 7:3 to afford **25** (110 mg, 0.215 mmol, 42%) as a white powder.

Molecular weight = 511.62 g mol^{-1}
Rf = 0.4 (c-Hex/EtOAc 3:7)
HRMS: Calcd. for $[C_{27}H_{37}N_5O_5 + Na]^+$: m/z 534.2692, found: 534.2688

^{1}H NMR (CD_3OD, 400 MHz): δ 7.37-7.29 (10H, m); 5.11 (2H, m); 4.70 (1H, m); 4.15 (1H, m); 3.34 (1H, m); 3.08 (1H, m); 2.91 (1H, m); 2.45 (1H, m); 1.76 (3H, s); 1.06 (1H, m); 1.03, 1.14 (6H, m); 0.68 (6H, m) ppm

^{13}C NMR (CD_3OD, 100 MHz): δ 176.0; 173.7; 158.7; 136.8; 136.8; 130.6; 128.9; 67.7; 56.8; 56.4; 48.7; 37.8; 27.1; 21.3; 19.9; 19.5 ppm
Melting point = 86-88°C
IR: 3312; 2926; 1692; 1495; 1351; 1213 cm^{-1}

(9H-fluoren-9-yl)methyl((*S*)-1-(((*S*)-1-amino-3-methyl-1-oxobutan-2-yl)amino-4-methyl-1-oxopentan-2-yl)carbamate

To a stirred solution of Fmoc-NH-Leu-OH (500 mg, 1.41 mmol, 1.0 eq.) in dry DMF (10 ml) were added successively under argon atmosphere at 0°C, HOBt (209 mg, 1.55 mmol, 1.1 eq.), HBTU (586 mg, 1.55 mmol, 1.1 eq.), DIPEA (0.74 mL, 4.23 mmol, 3.0 eq.) and *L*-Valinamide hydrochloride (215 mg, 1.41 mmol, 1.0 eq.). The reaction mixture was stirred overnight at room temperature. After filtration of the suspension formed, the solid obtained was dried to yield Fmoc-NH-Leu-Val-$CONH_2$ (620 mg, 1.37 mmol, 97%) as a white powder.

Molecular weight = 451.57 g mol^{-1}
Rf = 0.5 (EtOAc)
HRMS: Calcd. for $[C_{26}H_{33}N_3O_4 + H]^+$: m/z 452.2505, found: 452.2731

^{1}**H NMR** (DMSO-*d6*, 400 MHz): *δ* 8.00-7.25 (10H, m); 7.60 (1H, m); 7.07 (1H, bs); 4.34 (2H, m); 4.26 (1H, m); 4.18-4.07 (2H, m); 1.96 (1H, m); 1.62 (1H, m); 1.49 (2H, m); 0.98-0.84 (12H, m) ppm
^{13}**C NMR** (DMSO-*d6*, 100 MHz): *δ* 173.4; 144.6; 141.6; 128.5; 128.0; 126.1; 120.9; 66.3; 57.9; 54.3; 47.7; 41.6; 31.7; 25.1; 24.0, 22.3; 20.1; 18.8 ppm

***tert*-butyl ((*S*)-1-(((*S*)-1-(((*S*)-1-amino-3-methyl-1-oxobutan-2-yl)amino)-4-methyl-1-oxopentan-2-yl)amino)-1-oxo-3-phenylpropan-2-yl)carbamate**

To a solution of Fmoc-NH-Leu-Val-$CONH_2$ (600 mg, 1.33 mmol, 1.0 eq.) in dry DMF (10 mL) piperidine (12 mL, 20%v/DMF) was added. The solution was let under stirring for 3h and then the volatile was then removed under reduced pressure to afford the crude free amine which was used for the following coupling reaction without further purification. Precisely, to a solution of this crude free amine in DMF (10mL), under argon atmosphere and cooled to 0°C HOBt (360 mg, 2.66 mmol, 2.0 eq.), HBTU (1.0 g, 2.66 mmol, 2.0 eq.), DIPEA (0.93 mL, 5.32 mmol, 4.0 eq.) and *L*-Boc-NH-Phe-OH (706 mg, 2.66 mmol, 2.0 eq) were successively added. The reaction mixture was stirred at room temperature overnight. The volatile was removed under reduced pressure and after addition of EtOAc to the crude residue, Boc-NH-Phe-Leu-Val-$CONH_2$ precipitated as a white solid (550 mg, 1.16 mmol, 87%).
Molecular weight = 476.62 g mol^{-1}
Rf = 0.7 (EtOAc)
HRMS: Calcd. for $[C_{25}H_{40}N_4O_5 + H]^+$: m/z 477.3032, found: 477.3034; Calcd. for $[C_{25}H_{40}N_4O_5 + NH_4]^+$: M/z 494.2999, found: 494.3334
^{1}**H NMR** (DMSO-*d6*, 400 MHz): *δ* 8.07 (1H, d, *J* = 8.2 Hz); 7.68 (1H, d, *J* = 8.8 Hz); 7.38 (1H, bs); 7.30, 7.29 (4H, s); 7.21 (1H, m); 7.07 (1H, bs); 6.98 (1H, d, *J* = 8.81 Hz); 4.41 (1H, m); 4.24-4.11 (2H, m); 2.99 (1H, dd, *J* = 13.8, 3.8 Hz); 2.77 (1H, dd, *J* = 13.8, 10.6 Hz); 1.98 (1H, m); 1.68 (1H, m); 1.52 (2H, m), 1.33 (9H, s); 0.98-0.82 (12H, m) ppm
^{13}**C NMR** (DMSO-*d6*, 100 MHz): *δ* 173.6; 172.5; 139.2; 130.1, 128.9; 127.0; 79.1; 58.1; 56.6; 52.0; 41.7; 38.0; 31.6; 29.0; 25,0; 24.1, 22.5; 20.2, 18.8 ppm
IR: 3312; 2966; 1692-1676; 1632-1531 cm^{-1}

***Tert*-butyl 2-isobutylhydrazine-1-carboxylate [azaLeu]**

To a solution of *tert*-butylcarbazate (1 g, 7.6 mmol, 1 eq.) in dry THF (15 mL), under argon atmosphere isobutylraldehyde (1.0 mL, 11.4 mmol, 1.5 eq.) and acetic acid (0.524 mL, 6:81 mmol, 0.9 eq.) were successively added. After 5 h, the volatiles were removed under reduced pressure and the crude oil was dissolved in dry THF (15 mL). At that time, $NaBH_3CN$ (0.714 g, 11.4 mmol, 1.5 eq.) and acetic acid (0.866 mL, 15.1 mmol, 2 eq.) were added. The mixture was let under

stirring overnight. The volatile was removed under reduced pressure. The remaining powder was dissolved in a mixture of EtOAc and brine and then taken up again with EtOAc. The organic layer was washed with a mixture of 10% aqueous solution of NaCl/$NaHCO_3$ 1/1, dried over Na_2SO_4, filtered, and concentrated under reduced pressure. The crude residue obtained was then dissolved in MeOH (10 mL) and NaOH 1 M (9 mL, 9 mmol, 1.2 eq.) was added. The solution was let 1 h at room temperature under stirring. After removal of the volatile under reduced pressure, the obtained oil was taken up with EtOAc, washed with brine, dried over Na_2SO_4 and filtered. After concentration under reduced pressure, the residue obtained was purified by column chromatography on silica gel eluting with CH_2Cl_2 to afford **azaLeu** (0.95 g, 5.1 mmol, 67%) as a colorless oil.

Molecular weight = 188.15 g mol^{-1}

R*f* = 0.11 (CH_2Cl_2)
HRMS: Calcd. for $[C_9H_{20}N_2O_2 + H]^+$: m/z 189.1603, found: 189.1600
^{1}H NMR ($CDCl_3$, 300 MHz): δ 7.3 (1H, bs); 5.25 (1H, bs); 2.65 (2H, d, J = 6.8 Hz); 1.73 (1H, m); 1.44 (9H, s); 0.91 (6H, d, J = 6.7 Hz) ppm
^{13}C NMR ($CDCl_3$, 75 MHz): δ 157.3; 81.0; 60.4; 28.8; 27.1; 21.1 ppm
IR: 3312; 2920; 1719; 1259; 1157 cm^{-1}

Preparation of large unilamellar vesicles

The LUVs were composed of a mixture of DOPC/DOPS in a 7:3 molar ratio. Stock solutions of DOPC and DOPS in chloroform at concentrations of 20-30 mM were mixed in a glass tube. The solvent was evaporated with dry nitrogen gas yielding a lipid film that was subsequently kept in a vacuum desiccator for 1 h. Lipid films were hydrated during at least 30 minutes in the 10 mM Tris, 100 mM NaCl buffer at pH 7.4. The lipid suspensions were subjected to 10 freeze-thaw cycles, at temperatures of approximately -190°C and 50°C, respectively, and subsequently extruded 19 times through a mini-extruder (Avanti Alabaster, AL) equipped with polycarbonate membranes (at 200 nm cut-off). Calcein-containing vesicles were obtained by adding 70 mM calcein to the hydration buffer and free calcein were removed from the LUVs using size exclusion chromatography (Sephadex G50-fine), using the hydration buffer (10 mM Tris, 100 mM NaCl, pH 7.4) to as the mobile phase. The phospholipid content of lipid stock solutions and vesicle preparations was determined by assessing inorganic phosphate according to Rouser.

Fluorescence-Detected ThT Binding Assay (hIAPP)

hIAPP, purchased from Bachem, was dissolved in pure hexafluoro-isopropanol (HFIP) at a concentration of 1 mM and incubated for 1 hour at room temperature to dissolve any preformed aggregates. Next, HFIP was evaporated with dry nitrogen gas followed by vacuum desiccation for at least 3 hours. The resulting peptide film was then dissolved in DMSO to obtain stock solutions of hIAPP (0.2 mM) and stock solutions of compounds to test were dissolved in DMSO (10, 1 and

0.1 mM). The concentration of DMSO was kept constant at 3% (v/v) in the final volume of 200 µL. Thioflavin-T binding assays were used to measure the formation of fibrils in solution or in the presence of membranes over time. A plate reader (Fluostar Optima, BmgLabtech) and standard 96-wells flat-bottom black microtiter plates in combination with a 440 nm excitation and 480 nm emission filters were used. The ThT assay was started by adding 5 µL of a 0.2 mM hIAPP stock solution to a mixture of 10 µM ThT (obtained from Sigma) and 10 mM Tris/HCl, 100 mM NaCl at pH 7.4 containing 1 µL of stock solutions of compound to test. For the experiments in the presence of membranes, the fluorescence assays were started by adding 5 µL of a 0.2 mM hIAPP (5 µM peptide) to 195 µL of a mixture of 10 µM ThT, DOPC/DOPS (7:3) LUVs, with a peptide:lipid ratio of 1:20. The concentration of IAPP was held constant at 5 µM for all experiments and inhibitors were added to yield compound/IAPP ratios of 10/1, 1/1 and 0.1/1 (only at ratio 1/1 for the assays in membrane). The ThT assays were performed in triplicate and between 2 and 4 times on different days, with the same batch of peptide. The ability of compounds to inhibit IAPP aggregation was assessed considering the time of the half-aggregation (*t1/2*) and the intensity of the experimental fluorescence plateau (F), both values were obtained by fitting the obtained kinetic data to a Boltzmann sigmoidal curve using GraphPad Prism 5. The relative extension/reduction of *t1/2* is defined as the experimental *t1/2* in the presence of the tested compound relative to the one obtained without the compound and is evaluated as the following percentage: [*t1/2* (hIAPP + compound) − *t1/2* (hIAPP)] / *t1/2* (hIAPP) × 100. The relative extension/reduction of the experimental plateau is defined as the intensity of experimental fluorescence plateau observed with the tested compound relative to the value obtained without the compound and is evaluated as the following percentage: (FhIAPP + compound – FhIAPP) / FhIAPP × 100. The curves of the tested compounds are fitted to a Boltzmann sigmoidal model, normalized to the control experiment.

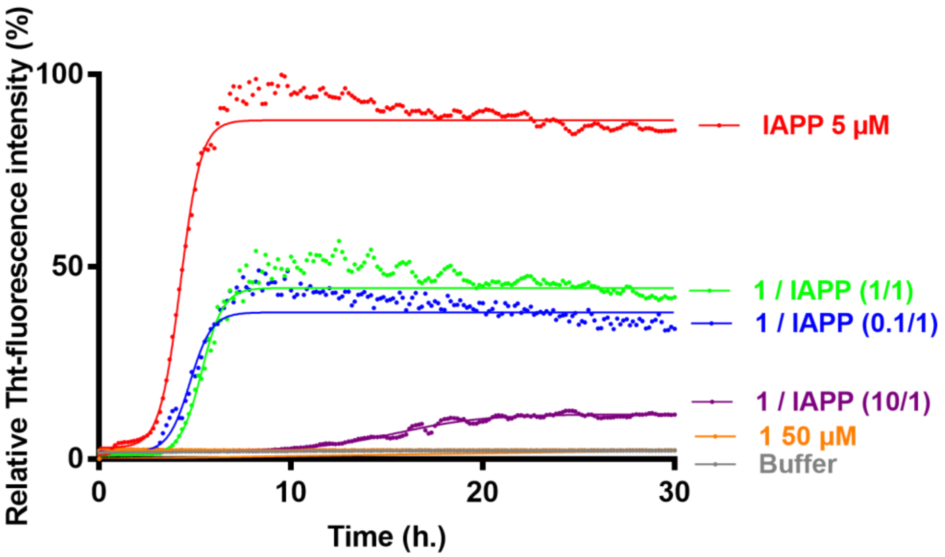

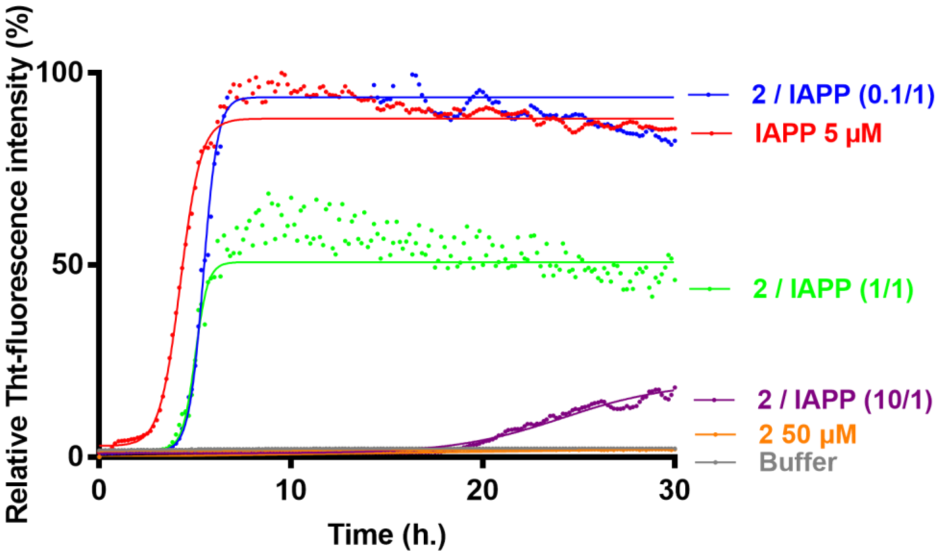

Relative Tht-fluorescence intensity (%)
100
50
0
0
10
20
30
Time (h.)
2 / IAPP (0.1/1)
IAPP 5 µM
2 / IAPP (1/1)
2 / IAPP (10/1)
2 50 µM
Buffer

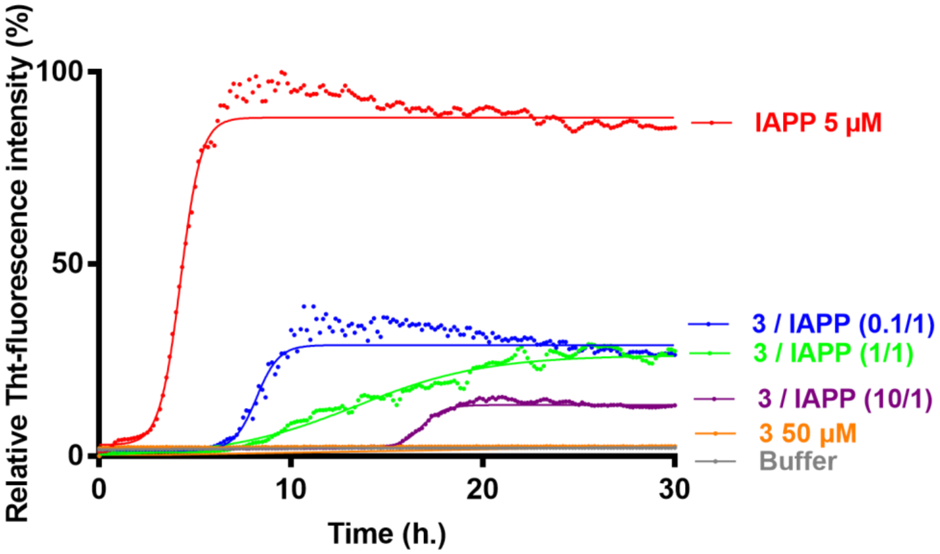

Relative Tht-fluorescence intensity (%)
100
50
0
0
10
20
30
Time (h.)
IAPP 5 µM
3 / IAPP (0.1/1)
3 / IAPP (1/1)
3 / IAPP (10/1)
3 50 µM
Buffer

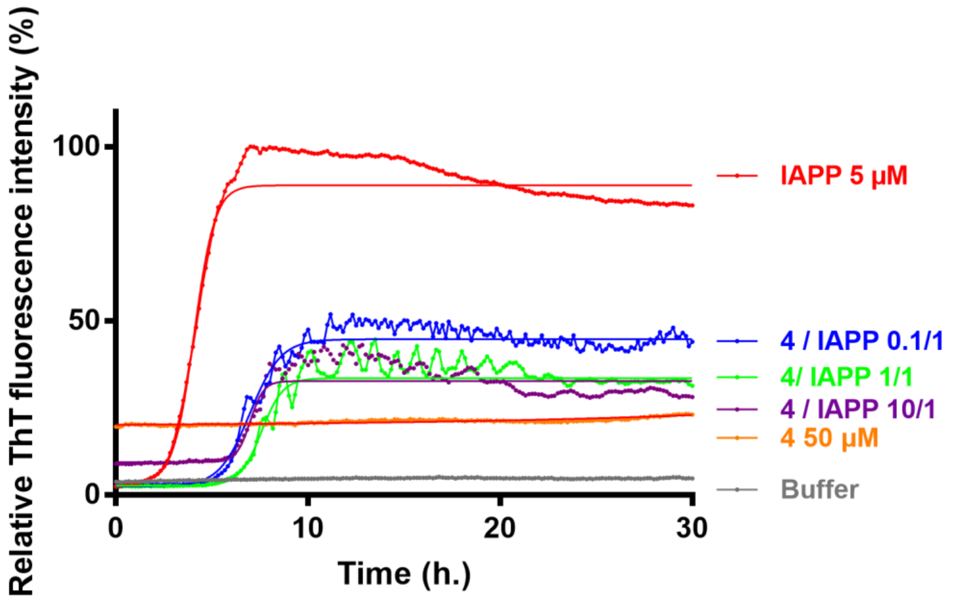

Relative ThT fluorescence intensity (%)
100
50
0
0
10
20
30
Time (h.)
IAPP 5 µM
4 / IAPP 0.1/1
4/ IAPP 1/1
4 / IAPP 10/1
4 50 µM
Buffer

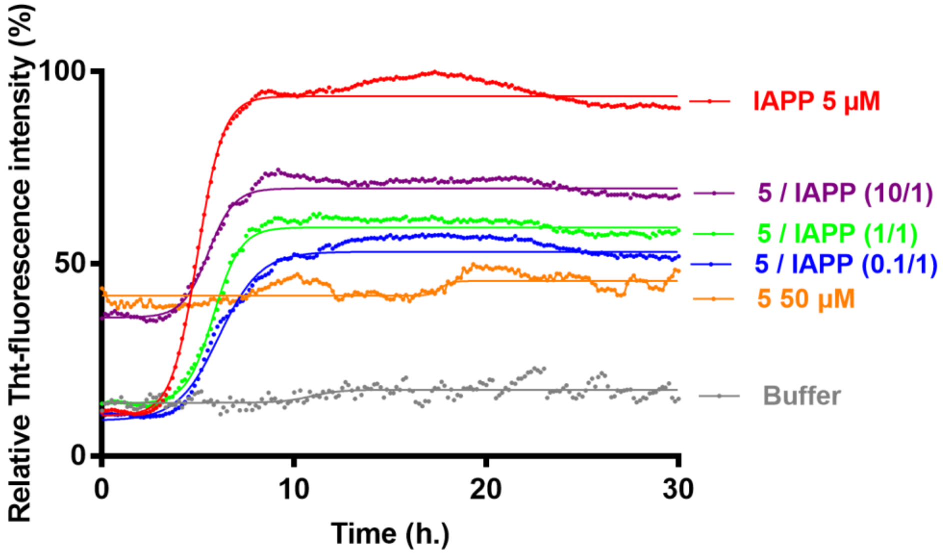

Relative Tht-fluorescence intensity (%)
100
50
0
0
10
20
30
Time (h.)
IAPP 5 µM
5 / IAPP (10/1)
5 / IAPP (1/1)
5 / IAPP (0.1/1)
5 50 µM
Buffer

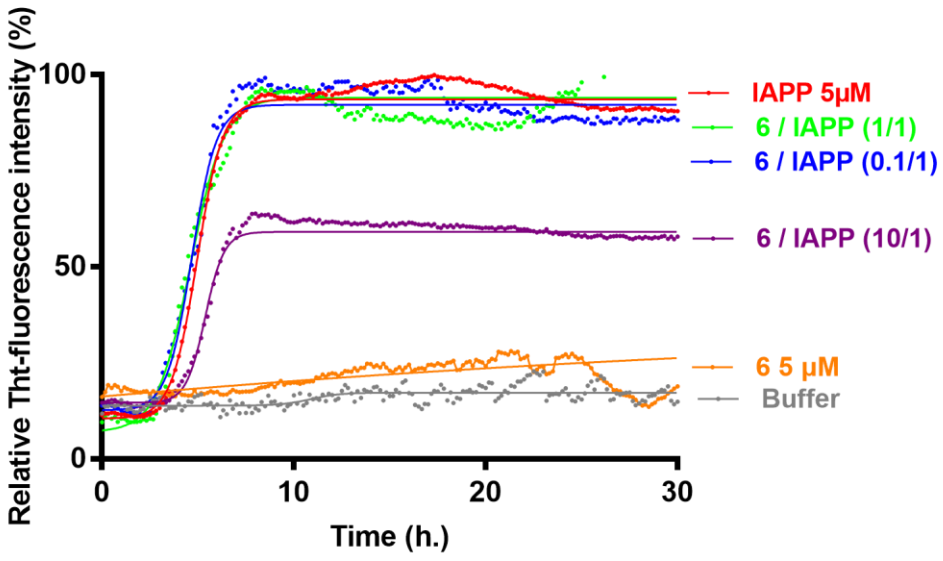

Relative Tht-fluorescence intensity (%)
100
50
0
0
10
20
30
Time (h.)
IAPP 5µM
6 / IAPP (1/1)
6 / IAPP (0.1/1)
6 / IAPP (10/1)
6 5 µM
Buffer

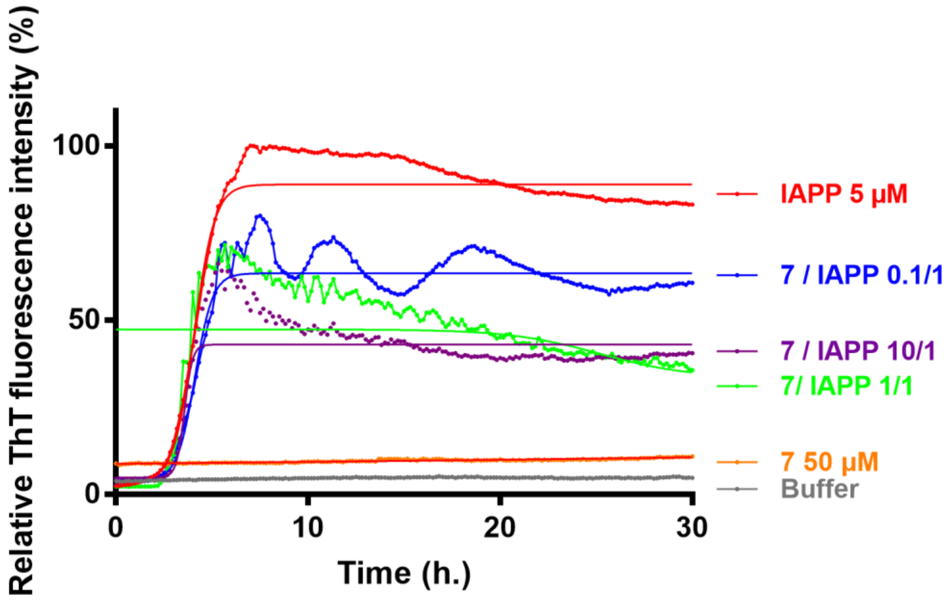

Relative ThT fluorescence intensity (%)
100
50
0
0
10
20
30
Time (h.)
IAPP 5 µM
7 / IAPP 0.1/1
7 / IAPP 10/1
7/ IAPP 1/1
7 50 µM
Buffer

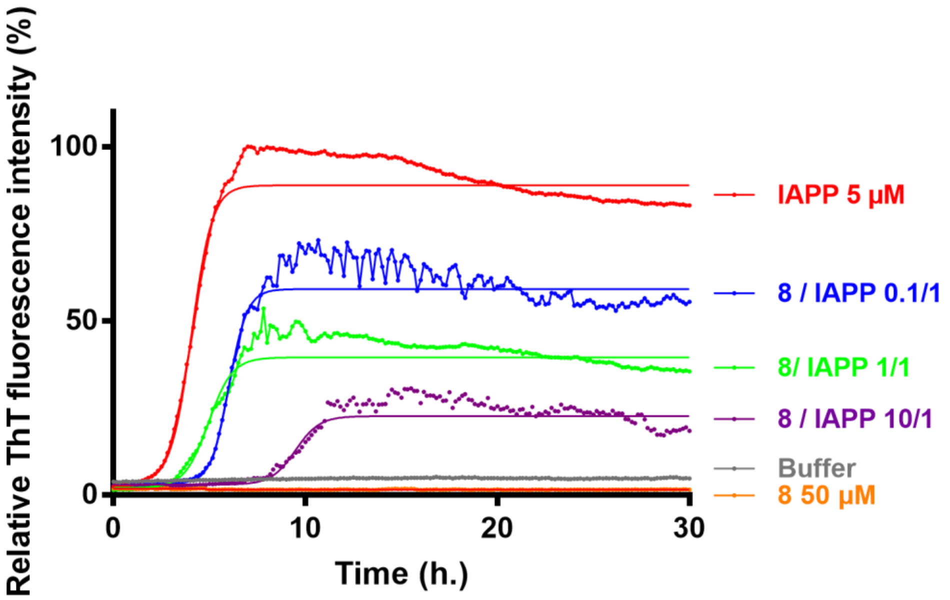

Relative ThT fluorescence intensity (%)
100
50
0
0
10
20
30
Time (h.)
IAPP 5 µM
8 / IAPP 0.1/1
8/ IAPP 1/1
8 / IAPP 10/1
Buffer
8 50 µM

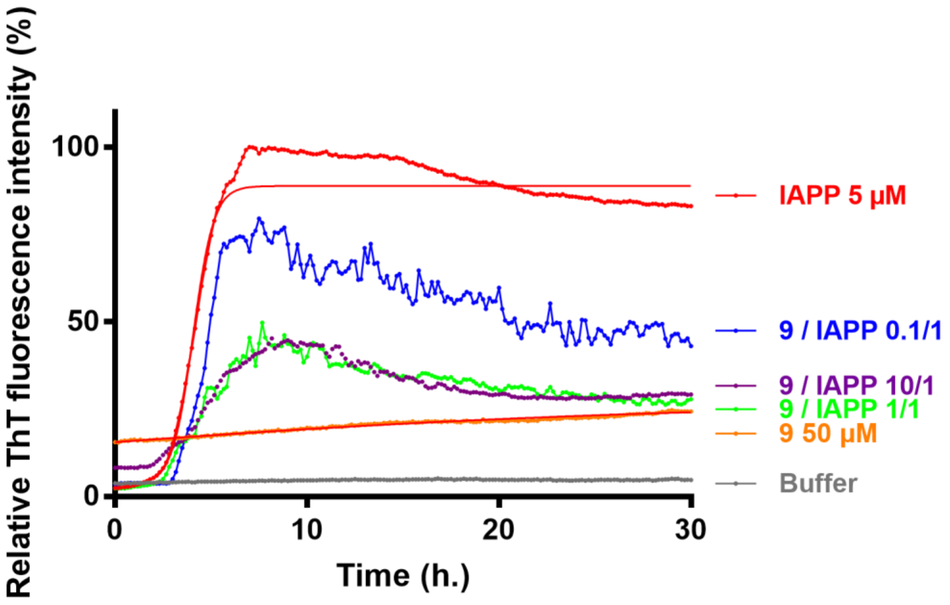

Relative ThT fluorescence intensity (%)
100
50
0
0
10
20
30
Time (h.)
IAPP 5 µM
9 / IAPP 0.1/1
9 / IAPP 10/1
9 / IAPP 1/1
9 50 µM
Buffer

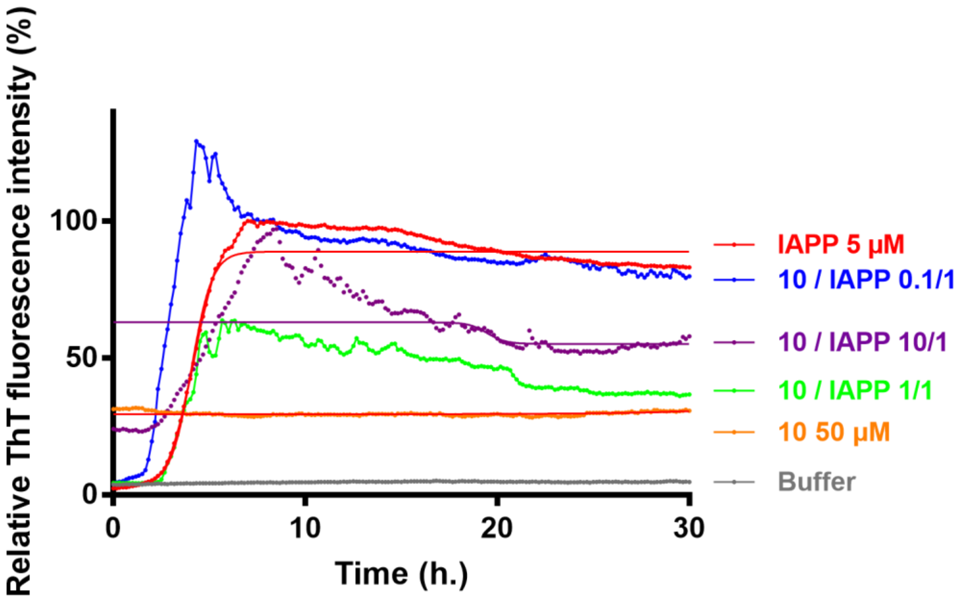

Relative ThT fluorescence intensity (%)
100
50
0
0
10
20
30
Time (h.)
IAPP 5 µM
10 / IAPP 0.1/1
10 / IAPP 10/1
10 / IAPP 1/1
10 50 µM
Buffer

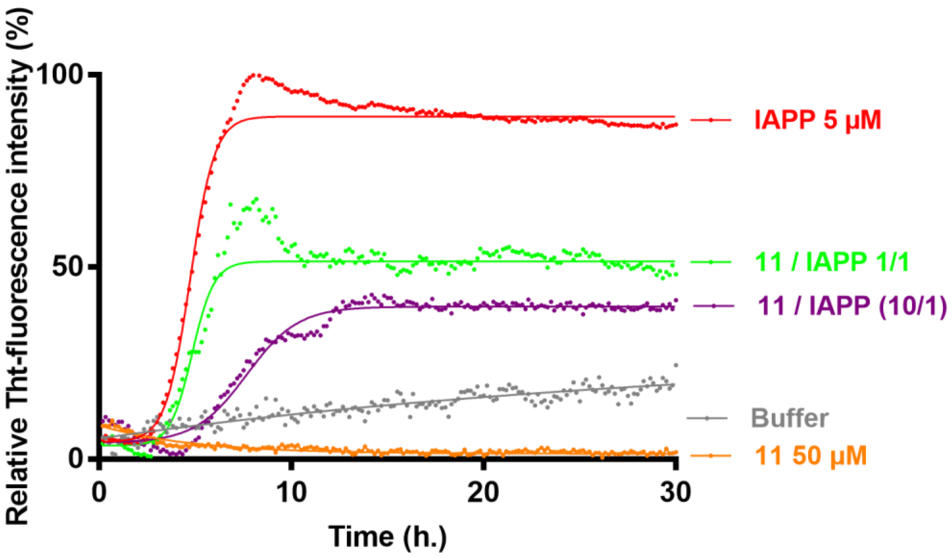

Relative Tht-fluorescence intensity (%)
100
50
0
0
10
20
30
Time (h.)
IAPP 5 µM
11 / IAPP 1/1
11 / IAPP (10/1)
Buffer
11 50 µM

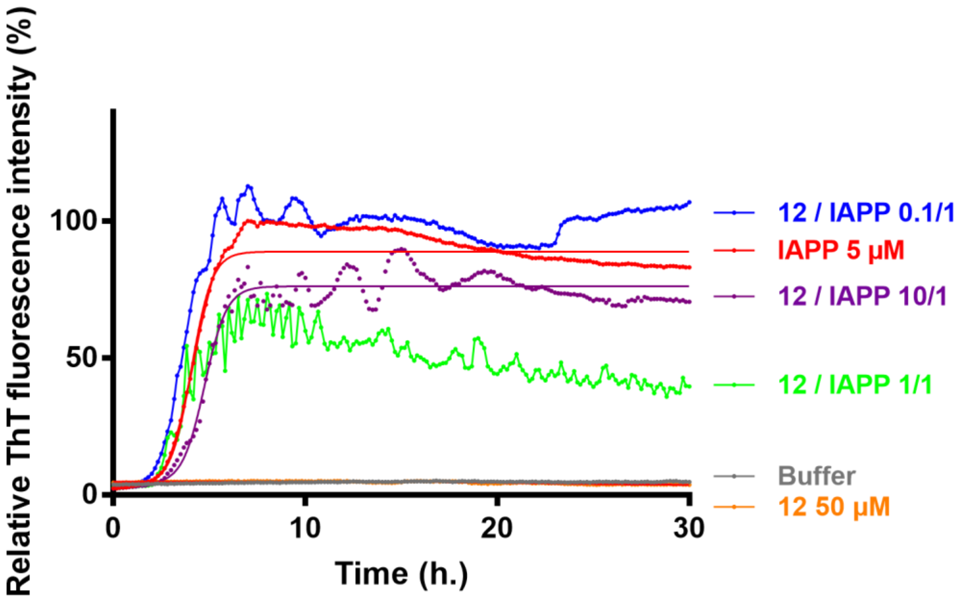
Relative ThT fluorescence intensity (%)
100
50
0
0
10
20
30
Time (h.)
12 / IAPP 0.1/1
IAPP 5 µM
12 / IAPP 10/1
12 / IAPP 1/1
Buffer
12 50 µM

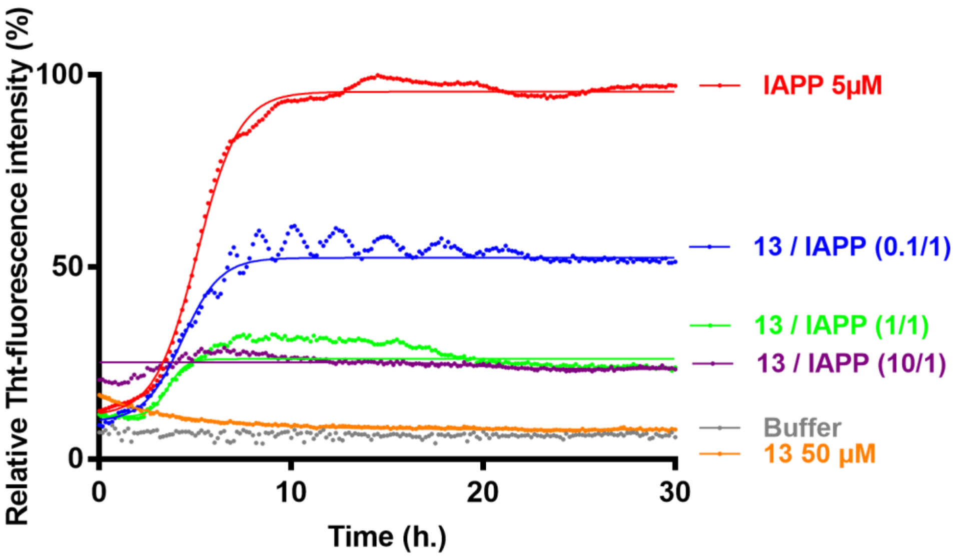
Relative Tht-fluorescence intensity (%)
100
50
0
0
10
20
30
Time (h.)
IAPP 5µM
13 / IAPP (0.1/1)
13 / IAPP (1/1)
13 / IAPP (10/1)
Buffer
13 50 µM

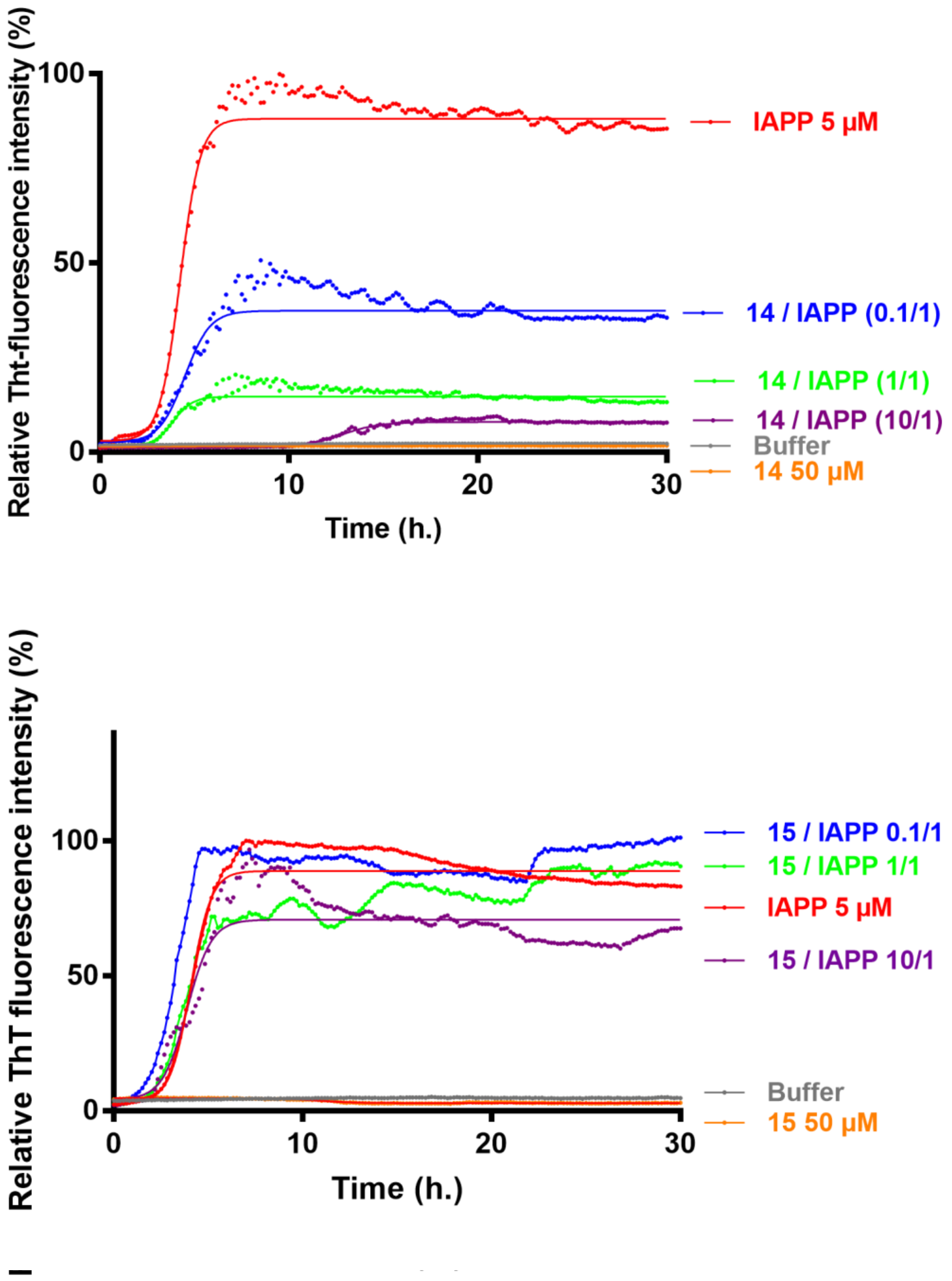

Figure S34. Representative curves of Tht fluorescence assays over time showing hIAPP aggregation (5 µM) in the absence (red curve) and in the presence of compounds **1-14** at compound/hIAPP ratios of 10/1 (purple curves), 1/1 (green curves) and 0.1/1 (blue curves). The control curves are represented in orange lines and buffer in grey.

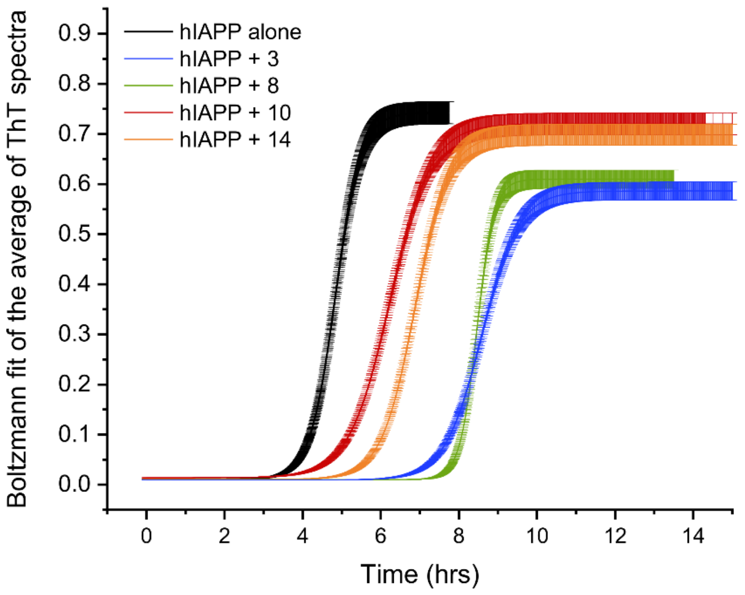

Figure S35. hIAPP fibrillation in the absence (black) and in the presence of the compounds **3** (blue), **8** (green), **10** (red) and **14** (orange) with a compound/hIAPP/lipid ratio of 1/1/20. The curves were all fitted using the Boltzmann equation.

Fluorescence-Detected ThT Binding Assay (Aβ_{1-42})

Aβ_{1-42} was purchased from Bachem and ThT was obtained from Sigma. The peptide was dissolved in an aqueous 1% ammonia solution to a concentration of 1 mM and then, just prior to use, was diluted to 0.2 mM with 10 mM Tris-HCl and 100 mM NaCl buffer (pH 7.4). Stock solutions of compounds to test were dissolved in DMSO with the final concentration kept constant at 0.5% (v/v). ThT fluorescence was measured to evaluate the development of Aβ_{1-42} fibrils over time using a fluorescence plate reader (Fluostar Optima, BMG labtech) with standard 96-well black microtiter plates (final volume in the wells of 200 µL). Experiments were started by adding the peptide (final Aβ_{1-42} concentration equal to 10 µM) into a mixture containing 40 µM ThT in 10 mM Tris-HCl and 100 mM NaCl buffer (pH 7.4) with and without the compounds at different concentrations (100, 10, 1 µM) at room temperature. The ThT fluorescence intensity of each sample (performed in triplicate) was recorded with 440/480 nm excitation/emission filters set for 42 h performing a double orbital shaking of 10 s before the first cycle. The fluorescence assays were performed between 2 and 4 times on different days, with the same batch of peptide. The ability of compounds to inhibit Aβ_{1-42} aggregation was assessed considering the time of the half-aggregation ($t_{1/2}$) and the intensity of the experimental fluorescence plateau (F), both values were obtained by fitting the obtained kinetic data to a Boltzmann sigmoidal curve using GraphPad Prism 5. The relative extension/reduction of $t_{1/2}$ is defined as the experimental $t_{1/2}$ in the presence of the tested compound relative to the one obtained without the compound and is evaluated as the following percentage: [$t_{1/2}$ (Aβ + compound) − $t_{1/2}$ (Aβ)] / $t_{1/2}$ (Aβ) × 100. The relative extension/reduction of the experimental plateau is defined as the intensity of experimental fluorescence plateau observed with the tested compound relative to the value obtained without the compound and is evaluated as the following percentage: (FAβ + compound – FAβ) / FAβ × 100.

Curves of the tested compounds are fitted to a Boltzmann sigmoidal model, normalized to the control experiment and represented below.

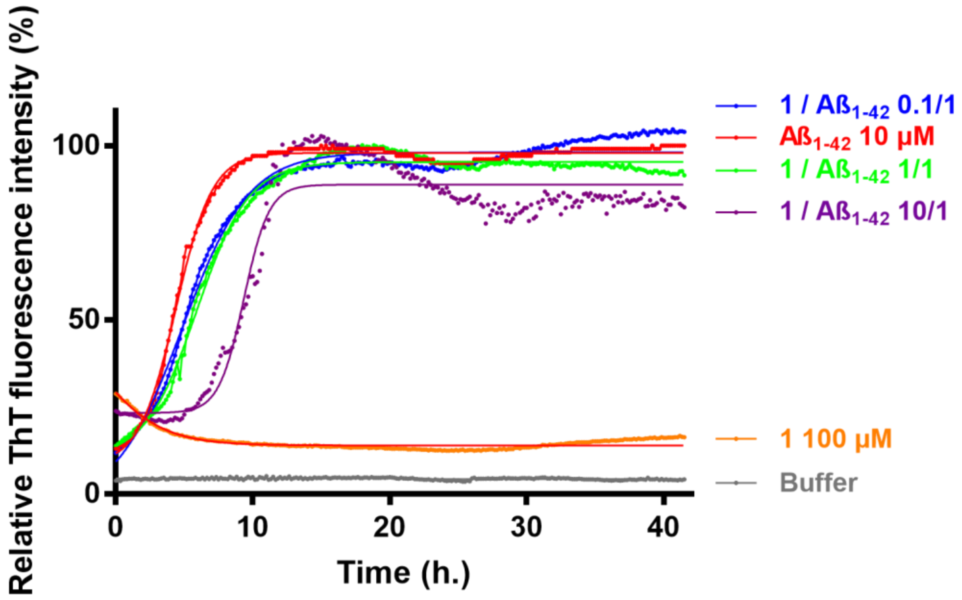

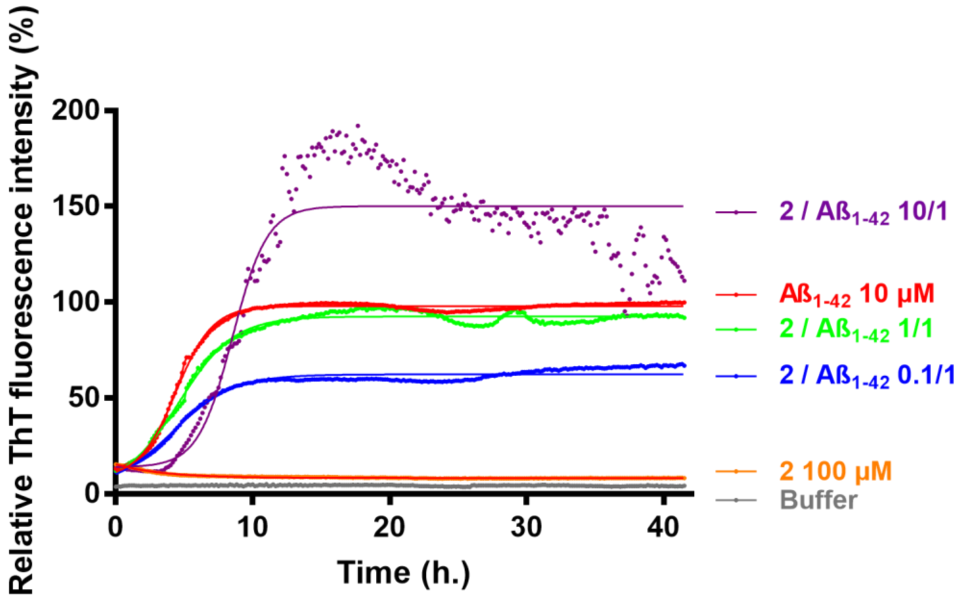

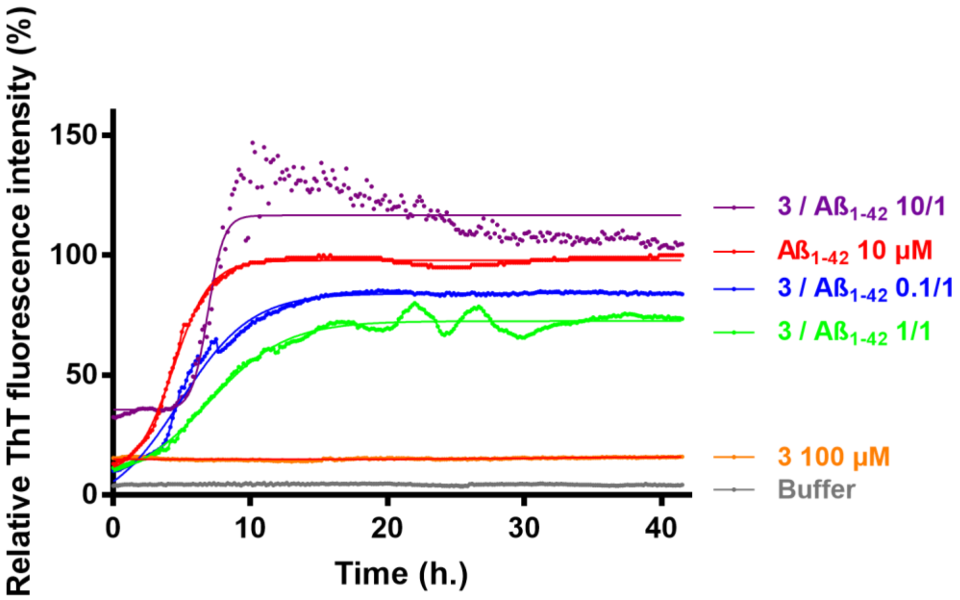
Relative ThT fluorescence intensity (%)
150
100
50
0
0
10
20
30
40
Time (h.)
3 / Aß1-42 10/1
Aß1-42 10 µM
3 / Aß1-42 0.1/1
3 / Aß1-42 1/1
3 100 µM
Buffer

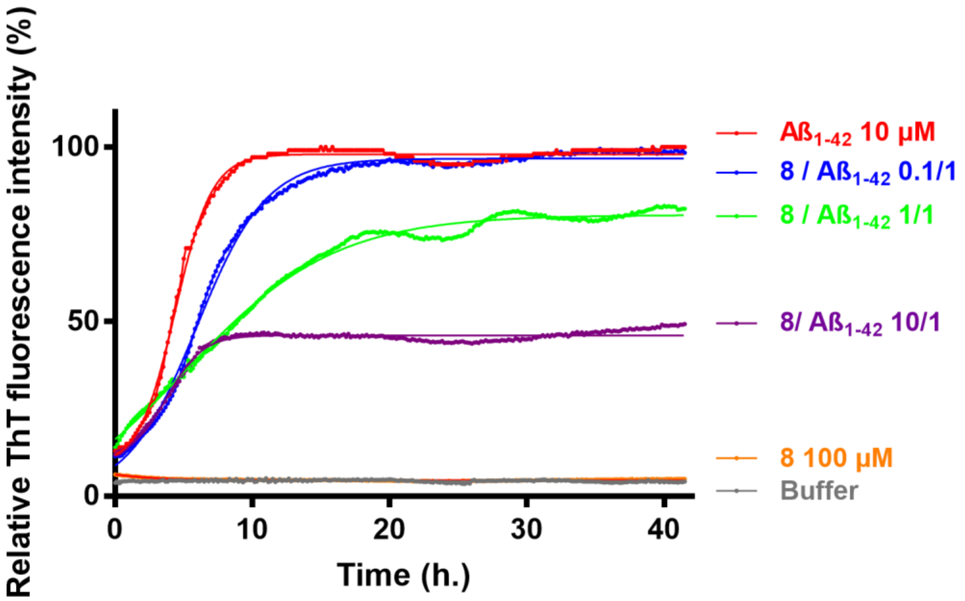
Relative ThT fluorescence intensity (%)
100
50
0
0
10
20
30
40
Time (h.)
Aß1-42 10 µM
8 / Aß1-42 0.1/1
8 / Aß1-42 1/1
8/ Aß1-42 10/1
8 100 µM
Buffer

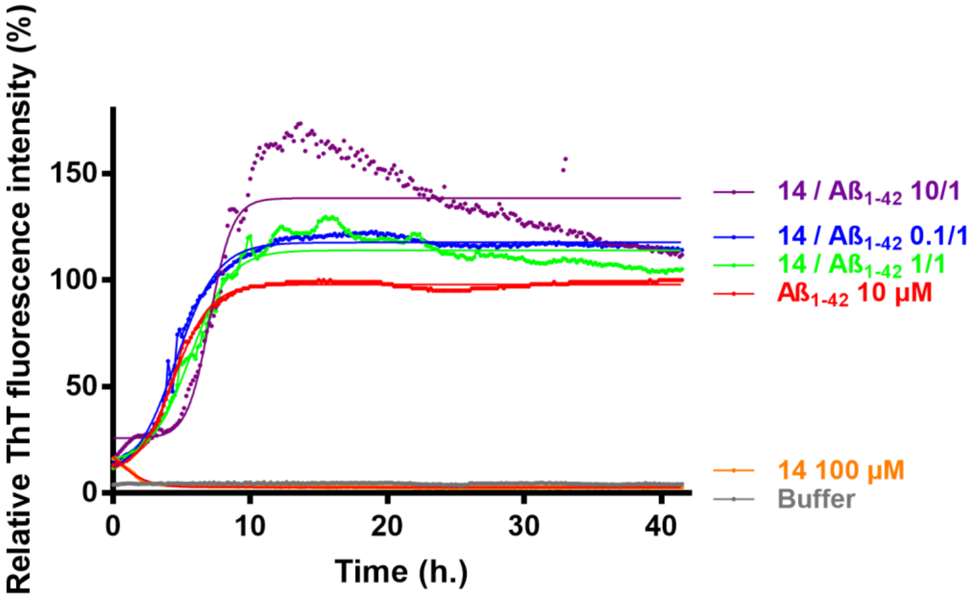

Figure S36. Representative curves of ThT fluorescence assays over time showing $A\beta_{1-42}$ (10 µM) aggregation in the absence (red curve) and in the presence of **1-3, 8** and **14** at compound/$A\beta_{1-42}$ ratios of 10/1 (purple curves), 1/1 (green curves) and 0.1/1 (blue curves). The control curves are represented in orange lines and buffer in grey.

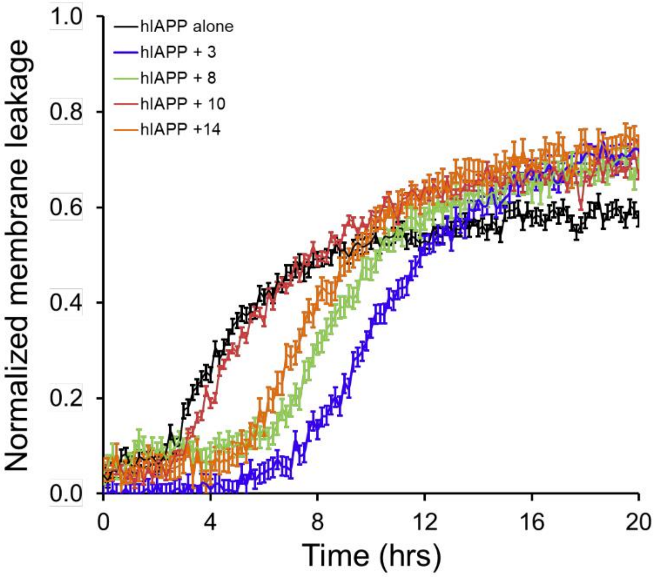

Figure S37. Representative curves of the kinetics of membrane disruption induced by 5 µM hIAPP in the absence (black) and in the presence of the compounds **3**, **8**, **10** and **14** (ratio 1/1) in 10 mM Tris-HCl, 100 mM NaCl buffer at pH 7.4.

Transmission Electron Microscopy

Samples were prepared under the same conditions as in the ThT-fluorescence assay. Aliquots of hIAPP (5 μM in 10 mM Tris-HCl, 100 mM NaCl, pH 7.4 in the presence and absence of hairpins **3**, **8**, **13** and **14** were adsorbed onto 300-mesh carbon grids for 2 min, washed and dried. The samples were negatively stained for 45 s. on 2 % uranyl acetate in water. After draining off the excess of staining solution and drying, the grids were observed using a JEOL 2100HC TEM operating at 200 kV with a LaB6 filament. Images were recorded in zero-loss mode with a Gif Tridiem energy-filtered-CCD camera equipped with a 2k x 2k pixel-sized chip (Gatan inc., Warrendale, PA). Acquisition was accomplished with the Digital Micrograph software (versions 1.83.842, Gatan inc., Warrendale, PA).

Membrane permeability assay

Leakage experiments were performed in standard 96-wells transparent microtiter plates using a plate reader (Spectrafluor, Tecan, Salzburg, Austria). Aliquots of 2.5 μL of molecules solution in DMSO at the desired concentration were added to 192.5 μL of 100 μM lipid vesicles in 10 mM Tris–HCl, 100 mM NaCl buffer at pH 7.4. The assay was then started by adding 5 μL of a 0.2 mM IAPP solution in DMSO or 2.5 μL DMSO only as control. Directly after addition of all components, the microtiter plate was shaken for 10 s. The plate was not shaken during the measurement. Fluorescence was measured from the top, every 5 min, using a 485 nm excitation filter and a 535 nm emission filter. The temperature during the measurement was 25 ± 3°C. The maximum leakage at the end of each measurement was determined by adding 2 μL of 10% Triton X-100 to a final concentration of 0.1% (v/v). The release of fluorescent dye was calculated according to the following equation:

$L(t) = (Ft – F0)/(F100 – F0)$

L(t) is the fraction of dye released (normalized membrane leakage) at time t, Ft is the measured fluorescence intensity at time t, and F0 and F100 are the fluorescence intensities at times t=0 and after addition of Triton X-100, respectively. All membrane leakage assays were performed three times, each in triplicate, on different days, using different IAPP stock solutions.
